# Integrating gene delivery and gene-editing technologies by adenoviral vector transfer of optimized CRISPR-Cas9 components

**DOI:** 10.1038/s41434-019-0119-y

**Published:** 2020-01-03

**Authors:** Ignazio Maggio, Hidde A. Zittersteijn, Qian Wang, Jin Liu, Josephine M. Janssen, Ivan Toral Ojeda, Silvère M. van der Maarel, Arjan C. Lankester, Rob C. Hoeben, Manuel A. F. V. Gonçalves

**Affiliations:** 1 Department of Pediatrics/Willem-Alexander Kinderziekenhuis (WAKZ), Leiden University Medical Center, Albinusdreef 2, 2300 RC Leiden, The Netherlands; 2 Department of Cell and Chemical Biology (CCB), Leiden University Medical Center, Einthovenweg 20, 2333 ZC Leiden, The Netherlands; 3 Department of Human Genetics, Leiden University Medical Centre, Einthovenweg 20, 2333 ZC Leiden, The Netherlands

**Keywords:** Genetic engineering, Genetic vectors

## Abstract

Enhancing the intracellular delivery and performance of RNA-guided CRISPR-Cas9 nucleases (RGNs) remains in demand. Here, we show that nuclear translocation of commonly used *Streptococcus pyogenes* Cas9 (SpCas9) proteins is suboptimal. Hence, we generated eCas9.4NLS by endowing the high-specificity eSpCas9(1.1) nuclease (eCas9.2NLS) with additional nuclear localization signals (NLSs). We demonstrate that eCas9.4NLS coupled to prototypic or optimized guide RNAs achieves efficient targeted DNA cleavage and probe the performance of SpCas9 proteins with different NLS compositions at target sequences embedded in heterochromatin versus euchromatin. Moreover, after adenoviral vector (AdV)-mediated transfer of SpCas9 expression units, unbiased quantitative immunofluorescence microscopy revealed 2.3-fold higher eCas9.4NLS nuclear enrichment levels than those observed for high-specificity eCas9.2NLS. This improved nuclear translocation yielded in turn robust gene editing after nonhomologous end joining repair of targeted double-stranded DNA breaks. In particular, AdV delivery of eCas9.4NLS into muscle progenitor cells resulted in significantly higher editing frequencies at defective *DMD* alleles causing Duchenne muscular dystrophy (DMD) than those achieved by AdVs encoding the parental, eCas9.2NLS, protein. In conclusion, this work provides a strong rationale for integrating viral vector and optimized gene-editing technologies to bring about enhanced RGN delivery and performance.

## Introduction

RNA-guided nucleases (RGNs) derived from prokaryotic adaptive immune systems are being repurposed for genome editing applications. Due to their ease of programmability, RGNs are revolutionizing our ability to manipulate the genome of higher eukaryote cells [1–4].

Among an increasing number of orthologs, the most commonly used RGNs are derived from the type II clustered, regularly interspaced, short palindromic repeats (CRISPR)-associated Cas9 (CRISPR-Cas9) system from *Streptococcus pyogenes* [5–7]. These RGNs work as a bipartite molecular scissor whose components are a Cas9 nuclease and a chimeric guide RNA (gRNA) [5–7]. The gRNA is an engineered single transcript consisting of a sequence-tailored CRISPR RNA (crRNA) linked to a transactivating crRNA (tracrRNA) moiety [1, 5, 6]. The 5′-end of the crRNA (spacer) directs specificity by binding to a DNA sequence through Watson–Crick base pairing. However, prior to crRNA hybridization to the target sequence, Cas9 needs to recognize a short protospacer adjacent motif (PAM), whose sequence is NGG in the case of *S. pyogenes* Cas9 (SpCas9). Hence, a 20-nucleotide (nt) sequence complementary to genomic DNA positioned next to a PAM defines a canonical target site for RGN complexes [1, 5–7].

After binding to the target site, the Cas9 nuclease undergoes conformational changes that lead to the activation of its two nuclease domains (i.e., RuvC and HNH) with subsequent generation of a double-stranded DNA break (DSB) [8, 9]. In somatic mammalian cells, DSBs are deleterious genomic lesions resolved by endogenous DNA repair pathways, most often through the nonhomologous end joining (NHEJ) pathway. The repair of RGN-induced DSBs by NHEJ can yield small insertions and deletions (indels) at predefined genomic positions. These indels can be exploited for functional knockout of genes and/or DNA motifs as well as restoration of out-of-frame coding sequences [1, 2].

Building on the extensive characterization of SpCas9 and gRNA structures [8–12], the efficiency and specificity of RGN-mediated genome editing are steadily improving by rationally designing individual CRISPR-Cas9 components or by directed protein evolution [4]. For instance, mutations in the PAM-interacting regions are the basis for SpCas9 variants with alternative PAM specificities [13, 14]. Furthermore, the amino acids substitutions N497A/R661A/Q695A/Q926A and N692A/M694A/Q695A/H698A have generated the high-specificity SpCas9-HF1 and HypaCas9 variants, respectively [15, 16]. These mutations are localized within REC3, a noncatalytic domain of SpCas9 involved in RNA–DNA heteroduplex recognition and in conformational activation of the HNH nuclease domain [15, 16]. Hence, by interfering with proofreading activity, the aforementioned alanine mutations in SpCas9-HF1 and HypaCas9 heighten the threshold for conformational HNH activation leading to increased specificity over wild-type SpCas9 [15, 16]. In another study, substitutions of residues interacting with the nontarget DNA strand (i.e., K848A/K1003A/R1060A) were shown to confer high specificity to the eSpCas9(1.1) variant [17]. In side-by-side comparisons, eSpCas9(1.1) tend to display higher on-target activities than those of SpCas9-HF1 [16, 18, 19].

Parallel efforts are also being devoted to improving the gRNA component. For instance, truncating the 5′-end of gRNAs by a few nts (Tru-gRNAs), can reduce off-target RGN cutting activity [20]. Furthermore, mutations disrupting a cryptic RNA polymerase III (Pol-III) terminator combined with the extension of the duplex located between the crRNA and tracrRNA moieties, have yielded optimized gRNA scaffolds which can improve RGN performance, presumably by increasing gRNA expression and stability in target cells [21, 22].

Despite constant efforts to optimize CRISPR-Cas9 components, further improvements on intracellular delivery and target DNA cleavage remain in demand for advancing genome editing in human cells [2, 23]. Here, to address these requirements, we assembled RGNs containing optimized SpCas9 and gRNA components. In particular, we investigated the effects on nuclear localization and targeted DSB frequencies of RGNs containing the high-specificity eSpCas9(1.1) nuclease with two or four nuclear localization signals (NLSs). NLSs are small peptides that mediate the nuclear import of cargo molecules to which they are either linked in artificial constructs or present in native proteins [24, 25]. Moreover, we tested the feasibility of coupling gRNAs harboring optimized scaffolds [22] to eSpCas9(1.1) with two or four NLSs. We report that endowing the high-specificity eSpCas9(1.1) nuclease [17], hereinafter named eCas9.2NLS, with two extra NLSs (eCas9.4NLS) improves protein nuclear compartmentalization, ultimately leading to enhanced targeted DSB formation. To further extend the delivery and hence applicability of these tools, we packaged the large expression units encoding SpCas9 variants in second-generation adenoviral vectors (AdVs). In contrast to other recombinant viral systems, e.g., lentiviral and adeno-associated viral vectors, AdVs have a strict episomal nature and large packaging capacity permitting high-level transitory expression of sizable RGN-encoding transgenes, both in vitro and in vivo [26–30].

## Results

### Construct designs for improving RGN performance in human cells

**Fig. 1:**
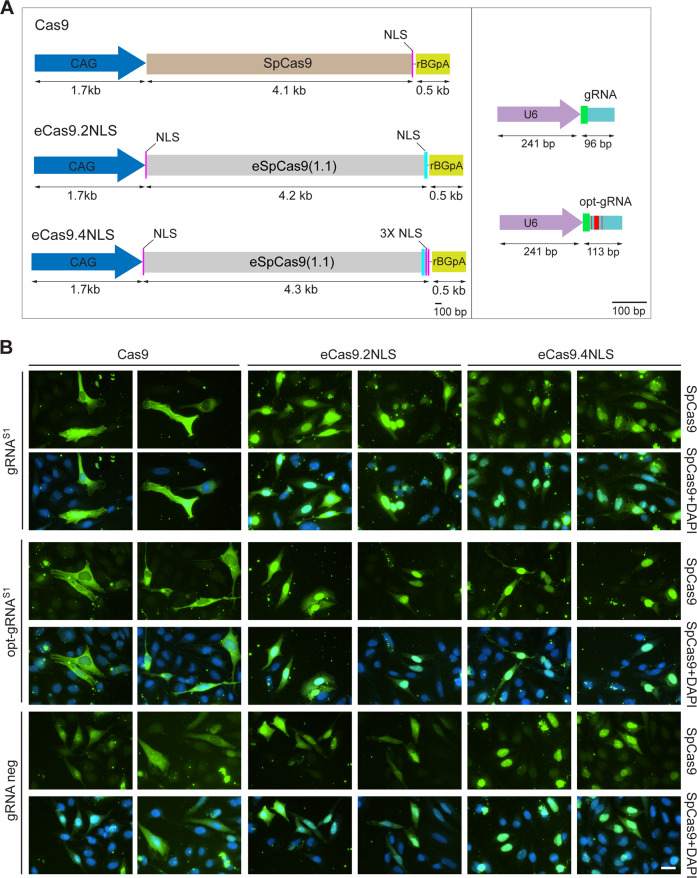
Schematics of the genome editing tools and assessment of cellular nuclease distribution. **a** Schematic representation of the constructs deployed to express the CRISPR-Cas9 components. The hybrid CAG promoter is composed of sequences from the cytomegalovirus (CMV) *immediate-early* enhancer, the first exon/intron of the chicken *β-actin* gene, and the splice acceptor of the rabbit *β-globin* gene. Magenta stripes, nuclear localization signal (NLS) from the SV40 large T antigen; cyan stripes, NLS from the nucleoplasmin of *Xenopus sp*.; rBGpA, rabbit *β-globin* polyadenylation signal; U6, RNA Pol-III promoter from the human *U6* gene; opt-gRNA, gRNA with an optimized tracrRNA; red stripes, nt differences between conventional gRNA and opt-gRNA scaffolds. **b** SpCas9 immunofluorescence microscopy on HeLa cells. HeLa cells transfected with the indicated combinations of constructs were stained for SpCas9. Two representative fluorescence microscopy images for each experimental condition were acquired at 5 days post transfection. The staining for SpCas9 proteins and DNA was performed with an antibody specific for the C-terminus of SpCas9 and DAPI, respectively. The white horizontal bar corresponds to 30 µm.

We started by transfecting HeLa cells with Cas9 or eCas9.2NLS plasmids. The Cas9 construct contains a human codon-optimized *SpCas9* open reading frame (ORF) linked to a SV40 large T antigen (SV40) NLS sequence [5], whereas the eCas9.2NLS construct contains the *eSpCas9(1.1)* ORF flanked by the SV40 and nucleoplasmin NLS sequences [17] (Fig. 1a). In this study, in which the performances of high-specificity eCas9.2NLS and eCas9.4NLS are compared, the regular prototypic Cas9 nuclease linked to a SV40 NLS served as a reference [5]. Thus, although each of these individual nucleases were assessed (i.e., Cas9, eCas9.2NLS and eCas9.4NLS), conclusions regarding the effect of extra NLSs are only drawn from comparing parental eCas9.2NLS with its eCas9.4NLS derivative (Fig. 1a).

Initial transfection experiments on HeLa cells followed by immunofluorescence microscopy analysis showed that substantial amounts of Cas9 and eCas9.2NLS proteins were located in the cytoplasm (Supplementary Fig. 1). Seeking to enhance the nuclear enrichment of high-specificity eCas9.2NLS, we generated eCas9.4NLS by adding two SV40 NLS coding motifs at the terminus of the *eCas9.2NLS* ORF (Fig. 1a). Moreover, to investigate whether different gRNA structures can impact the genome editing efficiencies when combined with high-specificity SpCas9 nucleases, we tested gRNAs with standard or optimized scaffolds (opt-gRNAs) (Fig. 1a). As aforementioned, the opt-gRNAs have a tracrRNA with an extended loop and a point mutation disrupting a cryptic RNA Pol-III pause signal [22, 31]. In side-by-side comparisons with conventional gRNAs, opt-gRNAs were shown to improve knockout efficiencies at *CCR5* and *CD4* when combined with regular SpCas9 [22]. Here, we probed the compatibility of opt-gRNAs with high-specificity eCas9.2NLS and the new eCas9.4NLS variant.

The expression of Cas9, eCas9.2NLS and eCas9.4NLS was assessed by transient transfection of HeLa cells. In this experiment, each of the nuclease expression constructs was cotransfected with plasmids encoding gRNA^S1^ or opt-gRNA^S1^ or, as negative controls, with plasmids lacking gRNA expression cassettes (i.e., gRNA neg). The gRNA^S1^ and opt-gRNA^S1^ molecules address the nucleases to the human *AAVS1* “safe harbor” locus at 19q13.42, and differ exclusively in that they have conventional and opt-gRNA scaffolds, respectively. Immunostaining for SpCas9 proteins followed by fluorescence microscopy analysis of transfected HeLa cells demonstrated a differential extent of nuclear localization between the different proteins with an increasing trend toward nuclear accumulation observed for those with two or four NLSs (Fig. 1b). This trend was similar independently of the type of gRNA used (Fig. 1b).

### Targeted *AAVS1* cleavage with optimized CRISPR-Cas9 components

**Fig. 2:**
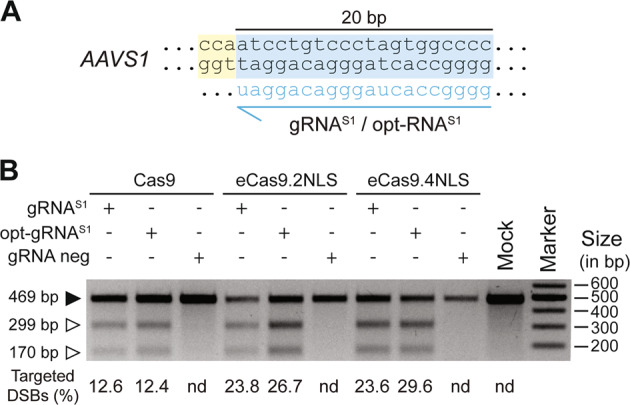
Detection of targeted DSB formation at *AAVS1* in HeLa cells. **a** Target site composed of gRNA-matching sequence (protospacer) and PAM (light blue and yellow boxes, respectively). This “safe harbor” *AAVS1* locus sequence locates within the first intron of the human *PPP1R12C* gene (19q13.42). **b** Detection of *AAVS1*-targeted DSB formation. Genomic DNA samples from Hela cells transfected with the indicated constructs were subjected to T7EI-based genotyping assays at 3 days post transfection. Cells transfected with plasmids lacking gRNA expression cassettes (gRNA neg) or mock-transfected provided for negative controls. Open and solid arrowheads indicate the positions of DNA species corresponding to digested and nondigested amplicons, respectively. Marker, GeneRuler DNA Ladder Mix; nd not detected.

Next, we sought to investigate the potential of combining eCas9.2NLS and eCas9.4NLS with opt-gRNAs for achieving efficient on-target DNA cleavage. To this end, we targeted the *AAVS1* locus owing to its common use as a “safe harbor” for achieving stable and homogeneous transgene expression in cell populations [32]. In these experiments, we exposed Hela cells to RGNs consisting of Cas9, eCas9.2NLS or eCas9.4NLS partnered with gRNA^S1^ or opt-gRNA^S1^ (Fig. 2a). As detected by T7 endonuclease I (T7EI)-based genotyping assays, eCas9.2NLS and eCas9.4NLS led to efficient NHEJ-derived indel formation at *AAVS1* when combined with opt-gRNA^S1^ (Fig. 2b). Importantly, the highest indel frequencies were achieved by combining eCas9.4NLS with opt-gRNA^S1^ (Fig. 2b).

### Assessing optimized CRISPR-Cas9 components in loss-of-function cellular systems

**Fig. 3:**
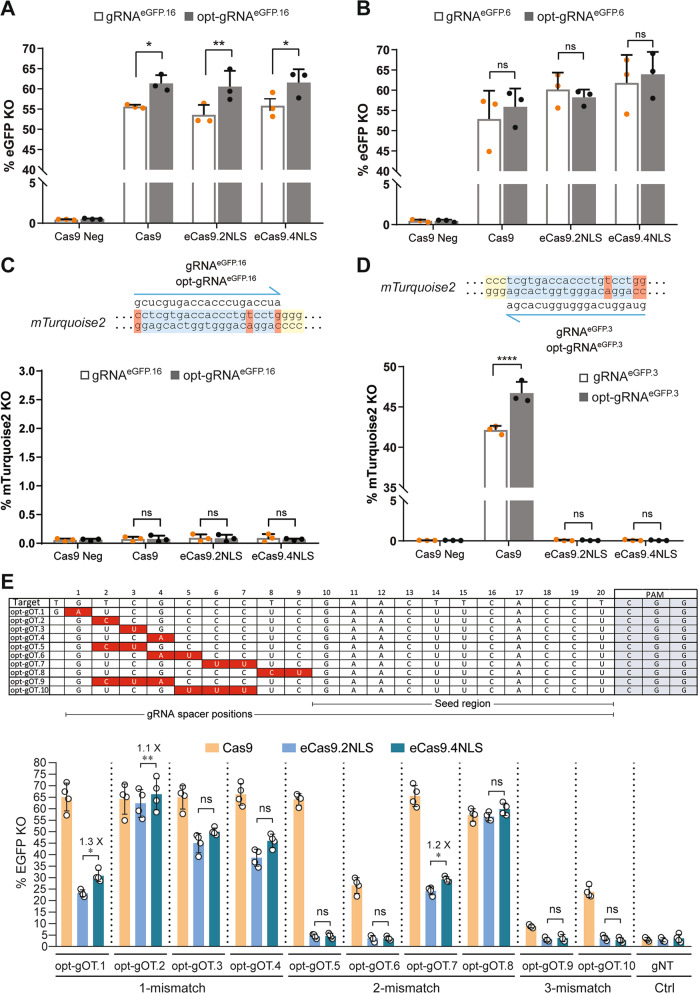
Evaluating the performance of optimized RGN complexes. **a**, **b** Testing the activity of optimized RGNs in reporter cells. Knockout levels in eGFP-positive H27 cells transfected with constructs expressing conventional or optimized RGNs. *eGFP* disruption frequencies were determined by flow cytometry of H27 cells transfected with the indicated constructs. Analysis was performed at 10 days post transfection. Bars and error bars represent mean and SD, respectively, from three independent experiments. Data significance was calculated with repeated measures one-way ANOVA followed by Tukey’s test for multiple comparisons: ns nonsignificant, *P* ≥ 0.05; *P < 0.05; **P < 0.01. **c**, **d** Testing the specificity of optimized RGNs in mTurquoise2-positive H11.7 cells. Upper panel, *mTurquoise2* target sites composed of gRNA-matching sequences (protospacers) and PAMs (light blue and yellow boxes, respectively). Red boxes highlight mismatching nucleotides between gRNAs and target sites. Lower panel, *mTurquoise2* disruption frequencies determined by flow cytometry of H11.7 transfected with the indicated constructs. Analysis was performed at 10 days post transfection. Bars and error bars correspond to mean and SD, respectively, from three independent experiments. Data significance was calculated with repeated measures one-way ANOVA followed by Tukey’s test for multiple comparisons: ns nonsignificant, *P* ≥ 0.05; ****P < 0.0001. **e** Testing the specificity of optimized RGNs in eGFP-positive H27 cells. Upper panel, sequences of opt-gRNAs opt-gOT.1 through opt-gOT.10 containing 1- 2- or 3-nt mismatches (red boxes) to a *eGFP* sequence (target). The canonical 20-nt long gRNA spacer positions and respective 10-nt long seed region, are delimited. Mismatches locating within the seed region are purportedly more detrimental to RGN activity than those present outside this region. PAM, protospacer adjacent motif. Lower panel, *eGFP* knockout frequencies determined by flow cytometry of H27 cells transfected with the indicated plasmids. A *eGFP* nontargeting gRNA (gNT) was used as negative control. Analysis was carried out at 10 days post transfection. Bars and error bars correspond to mean and SD, respectively from four independent experiments (except for opt-gOT.9 where the bars and error bars correspond to mean and SD from three independent experiments). Significance between the indicated datasets was calculated by two-tailed Student’s *t* tests: ns nonsignificant, *P* ≥ 0.05; *P < 0.05; **P < 0.01.

For a more sensitive assessment of RGN activities, we performed gene knockout experiments targeting *eGFP* or *mTurquoise2* reporter genes. We started by targeting two different sequences in *eGFP* by deploying opt-gRNA^eGFP.16^ and opt-gRNA^eGFP.6^ (Fig. 3a, b and Supplementary Table 1). Gene knockout levels mediated by opt-gRNA^eGFP.16^ and opt-gRNA^eGFP.6^ were compared with those achieved by gRNA^eGFP.16^ and gRNA^eGFP.6^ containing the conventional scaffold (Fig. 3a, b and Supplementary Table 1). The gRNA expression plasmids were cotransfected with Cas9, eCas9.2NLS or eCas9.4NLS constructs into H27 cells. The H27 cell line is a HeLa-derived clone containing a single-copy, constitutionally active, *eGFP* transcriptional unit [33]. NHEJ-mediated repair of RGN-induced DSBs introduces frame-shifting indels that lead to *eGFP* knockout whose frequencies can be quantified by flow cytometry [28]. Transfection experiments in H27 cells revealed that RGNs consisting of eCas9.2NLS:opt-gRNA^eGFP.16^ and eCas9.4NLS:opt-gRNA^eGFP.16^ complexes led to robust *eGFP* knockout (Fig. 3a and Supplementary Table 1). Furthermore, opt-gRNA^eGFP.16^ yielded higher knockout levels than those obtained by its nonoptimized gRNA^eGFP.16^ counterpart (Fig. 3a and Supplementary Table 1). Similar experiments deploying instead opt-gRNA^eGFP.6^ and gRNA^eGFP.6^ confirmed that the eCas9.2NLS and eCas9.4NLS are compatible with the use of gRNAs with optimized scaffolds (Fig. 3b and Supplementary Table 1).

Tru-gRNAs can increase the specificity of RGNs containing the conventional SpCas9 nuclease [20]. However, combining Tru-gRNAs with eCas9.2NLS often results in reduced on-target activity [17, 34–36]. To probe the effect of combining eCas9.4NLS with truncated opt-gRNAs on targeted DNA cleavage, we generated constructs expressing gRNA^eGFP.5^ and opt-gRNA^eGFP.5^ whose target site is 1-nt shorter than that of gRNA^eGFP.6^ and opt-gRNA^eGPF.6^ (Supplementary Fig. 2). After transfecting H27 cells with plasmids expressing gRNA^eGFP.5^ or opt-gRNA^eGFP.5^ each mixed with plasmids encoding Cas9, eCas9.2NLS or eCas9.4NLS, gene knockout frequencies were again determined by flow cytometry. Expression of eCas9.2NLS:gRNA^eGFP.5^ and eCas9.4NLS:gRNA^eGFP.5^ complexes led to *eGFP* knockout frequencies that where not higher than those obtained with Cas9:gRNA^eGFP.5^ (Supplementary Fig. 2 and Supplementary Table 2). Albeit not statistically significant, deploying opt-gRNA^eGFP.5^ yielded a slight increase in target DNA cleavage when compared to that achieved with gRNA^eGFP.5^, regardless of the SpCas9 partner selected (i.e., Cas9, eCas9.2NLS or eCas9.4NLS) (Supplementary Fig. 2 and Supplementary Table 2).

As RGNs consisting of opt-gRNAs and eCas9.2NLS or opt-gRNAs and eCas9.4NLS generate robust on-target DNA cleavage, we next sought to investigate whether these RGNs retain a high-specificity profile. For this purpose, an off-target RGN activity detection system based on H11.7 cells was set up. The H11.7 cell line is a HeLa-derived clone whose genome contains a single copy of a constitutionally active *mTurquoise2* transcriptional unit [37]. In these experiments, H11.7 cells were exposed to Cas9, eCas9.2NLS or eCas9.4NLS together with gRNA^eGFP.16^ or with opt-gRNA^eGFP.16^. Crucially, at the *mTurquoise2* allele, *eGFP*-specific gRNA^eGFP.16^ and opt-gRNA^eGFP.16^ have 2-nt mismatches at the PAM-proximal side and 1-nt mismatch at the most PAM-distal position (Fig. 3c). Thus, these and other polymorphisms distinguishing *eGFP* from *mTurquoise2* turn H11.7 cells, together with *eGFP*-specific gRNAs, into a reporter system for off-target RGN activities through the quantification of mTurquoise2-negative populations. Flow cytometric analysis of transfected H11.7 cells revealed that the 3-nt mismatches between *mTurquoise2* and the gRNA sequences in gRNA^eGFP.16^ and opt-gRNA^eGFP.16^ were enough to abolish DNA cutting activity, regardless of the nuclease variant applied (Fig. 3c and Supplementary Table 1). To further probe for specificity, we took advantage of the DNA sequence flanking the off-target site of gRNA^eGFP.16^ and opt-gRNA^eGFP.16^. This sequence allowed designing “inverted” gRNA^eGFP.3^ and opt-gRNA^eGFP.3^ whose DNA-matching sequence overlaps with that of gRNA^eGFP.16^ and opt-gRNA^eGFP.16^ (Fig. 3d). Therefore, in contrast to gRNA^eGFP.16^ and opt-gRNA^eGFP.16^, gRNA^eGFP.3^ and opt-gRNA^eGFP.3^ have 3-nt mismatches exclusively at the PAM-distal end of their off-target sequences (Fig. 3d). Since mismatches at the PAM-distal side are usually more tolerated than those at the PAM-proximal end [38, 39], deploying gRNA^eGFP.3^ and opt-gRNA^eGFP.3^ allowed us testing off-target RGN activities under more stringent conditions than those pertaining to gRNA^eGFP.16^ and opt-gRNA^eGFP.16^ (Fig. 3c). Exposing H11.7 cells to Cas9:gRNA^eGFP.3^ complexes readily yielded high frequencies of mTurquoise2-negative cells (Fig. 3d and Supplementary Table 1). Of note, the off-target activity of Cas9 in H11.7 cells was significantly higher when coupled to opt-gRNA^eGFP.3^ instead of gRNA^eGFP.3^ (Fig. 3d and Supplementary Table 1). These data suggest that opt-gRNAs can increase off-target activities if combined with the native Cas9 protein. Crucially, in contrast to Cas9, neither eCas9.2NLS nor eCas9.4NLS led to detectable *mTurquoise2* disruption when coupled to gRNA^eGFP.3^ or opt-gRNA^eGFP.3^ (Fig. 3d and Supplementary Table 1). To broaden the comparison of the specificity profiles of eCas9.2NLS versus eCas9.4NLS at different off-target sites, we generated a panel of constructs expressing opt-gRNAs (opt-gOT.1 through opt-gOT.10) with different nt mismatches to an *eGFP* target sequence (Fig. 3e, top panel). Flow cytometric analysis of *eGFP*-expressing H27 cells [33] exposed to complexes between Cas9, eCas9.2NLS or eCas9.4NLS and each of these opt-gRNAs established that eCas9.4NLS retains most of the high specificity of its parental eCas9.2NLS protein (Fig. 3e, bottom panel and Supplementary Table 3). Indeed, the cumulative data gathered in Fig. 3c–e show that amongst the 12 gRNAs with mismatches tested only three conferred statistically significant higher off-target activities to eCas9.4NLS when compared to eCas9.2NLS (i.e., opt-gOT.1, opt-gOT.2, and opt.gOT.7). Importantly, these off-target activity increments associated with opt-gOT.1, opt-gOT.2, and opt-gOT.7 were low, i.e., 1.3-fold, 1.1-fold, and 1.2-fold, respectively (Fig. 3e, bottom panel and Supplementary Table 3).

**Fig. 4:**
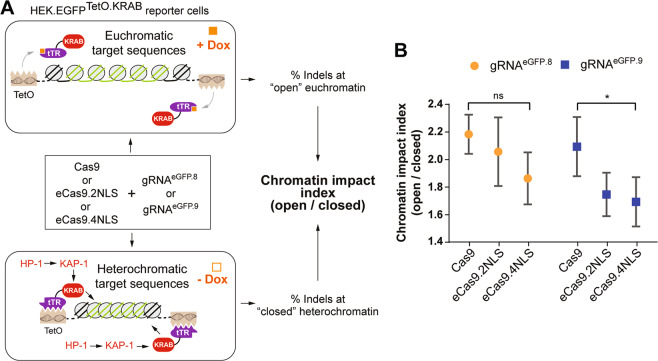
Probing the effect of chromatin conformation on the activity of Cas9 nucleases with different NLS content. **a** Comparing the chromatin impact indexes of conventional versus optimized SpCas9 nucleases. The HEK.EGFP^TetO.KRAB^ reporter cells, incubated in the presence or absence of Dox, were transfected with the indicated RGN components. HEK.EGFP^TetO.KRAB^ cells contain single-copy *eGFP* alleles flanked by *TetO* elements. In the absence of Dox (−Dox), tTR-KRAB fusion proteins bind to *TetO* elements and induce heterochromatin formation through the recruitment of, amongst other factors, KAP1 and HP1. In the presence of Dox, tTR-KRAB unbinds from *TetO* elements ultimately leading the *eGFP* target sequences to acquire an “open” euchromatic state. To determine indel frequencies, Dox was added to all cultures (i.e., preincubated or not with Dox) prior to eGFP-direct flow cytometry. The chromatin impact indexes correspond to the ratios between *eGFP* knockout levels at euchromatic (+Dox) versus heterochromatic (−Dox) target sites. **b** Chromatin impact indexes probed in HEK.EGFP^TetO.KRAB^ reporter cells. HEK.EGFP^TetO.KRAB^ cells were transfected with constructs coding for the indicated RGN elements. *eGFP* knockout frequencies at euchromatic (+Dox) and heterochromatic (−Dox) target sites are presented in Supplementary Fig. 3C. Error bars indicate mean ± SEM corresponding to three independent experiments. Data significance was calculated with repeated measures one-way ANOVA followed by Tukey’s test for multiple comparisons: ns nonsignificant, *P* ≥ 0.05; *P < 0.05.

The DNA binding and cleaving activities of RGNs are, to some extent, hindered at heterochromatic regions in eukaryotic cells [34, 40–44]. Hence, we next examined the degree with which different chromatin structures impact the performance of Cas9, eCas9.2NLS and eCas9.4NLS. To this end, we deployed a readout system based on tTR-KRAB-expressing HEK.EGFP^TetO.KRAB^ cells [34, 40, 45]. These cells contain single-copy *eGFP* alleles flanked by *TetO* elements [34, 40, 45]. In the absence of doxycycline (Dox), the binding of the tTR-KRAB fusion protein to its cognate *TetO* elements recruits endogenous KAP1-HP1-associated epigenetic remodeling complexes that trigger heterochromatin formation at *eGFP* sequences (Fig. 4a). In the presence of Dox, tTR-KRAB suffers a conformational change preventing it from binding to the *TetO* elements, ultimately leading *eGFP* sequences to acquire an “open” euchromatic state [34, 40, 45] (Fig. 4a).

Gene knockout experiments in HEK.EGFP^TetO.KRAB^ cells, incubated with or without Dox, were performed by deploying plasmids encoding gRNA^eGFP.8^ and gRNA^eGFP.9^ (Supplementary Fig. 3a). These gRNA expression plasmids were cotransfected with Cas9, eCas9.2NLS or eCas9.4NLS constructs. After confirming that the different plasmid mixtures yielded similar transfection efficiencies (Supplementary Fig. 3b and Supplementary Table 4), the cell populations were subcultured for 10 days, during which time the various RGNs were expected to induce DSBs at either euchromatic (+Dox) or heterochromatic (−Dox) isogenic target sites. Next, all cell cultures were exposed to Dox to allow for expression and quantification of *eGFP* knockout frequencies via flow cytometry (Supplementary Fig. 3c and Supplementary Table 5). Flow cytometric analysis confirmed the results of previous reports showing that the activity of RGNs can be hindered by KRAB-induced heterochromatin [34, 40, 45] (Supplementary Fig. 3c and Supplementary Table 5). Moreover, we detected a trend toward lower ratios between the frequencies of DSB formation at euchromatic versus heterochromatic target sites (i.e., chromatin impact indexes) when RGNs contained eCas9.2NLS or eCas9.4NLS instead of Cas9 (Fig. 4b and Supplementary Table 6). These data suggest that eCas9.2NLS and eCas9.4NLS are more capable of overcoming the compact heterochromatic barrier than Cas9.

### AdV-mediated delivery of SpCas9 nucleases and assessment of their subcellular localization

**Fig. 5:**
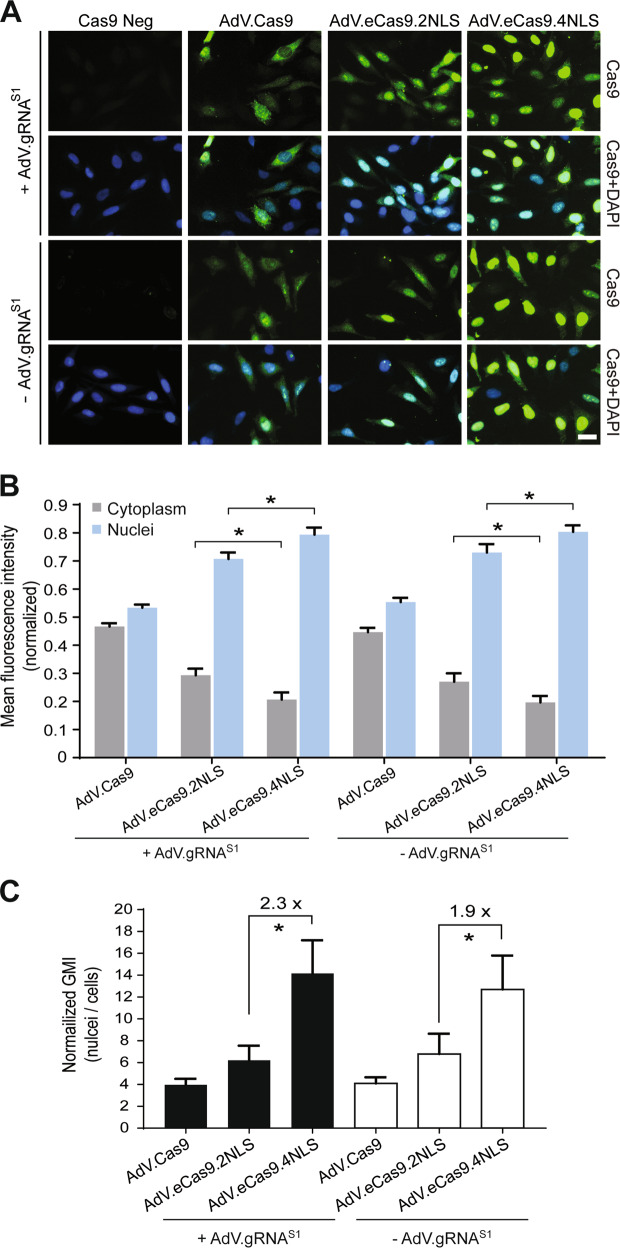
Quantification of SpCas9 intracellular distribution in AdV-transduced HeLa cells. **a** Representative immunofluorescence microscopy images of HeLa cells transduced with the indicated AdVs. The staining for SpCas9 nucleases and DNA was done at 5 days post transduction using an antibody specific for the C-terminus of SpCas9 and DAPI, respectively. The horizontal bar corresponds to 30 µm. **b** Mean fluorescence intensity (MFI) of SpCas9-specific signals in the nucleus versus cytoplasm of AdV-transduced cells. Values correspond to the mean of nuclear or cytoplasmic SpCas9-derived fluorescence intensities normalized, on a per-cell-basis, to the whole cellular fluorescence intensity. Bars and error bars indicate mean and SD, respectively, of three independent experiments. Significance between the indicated datasets was calculated by two-tailed Student’s *t* tests; *P < 0.05. **c** SpCas9 nu**c**lear enrichment. Values are calculated by normalizing the geometric mean intensity (GMI) in the nucleus versus the GMI in whole cell. Bars and error bars indicate mean and SD, respectively, of three independent experiments. Significance between the indicated datasets was calculated by two-tailed Student’s *t* tests; *P < 0.05. Data were acquired with Xcyto 10 using 20 ×  magnification. Automated cell segmentation was performed with XcytoView software.

The size of the eCas9.4NLS expression unit (i.e., ∼6.5 kb; Fig. 1a), exceeds the effective packaging capacity of commonly used viral vectors, such as lentiviral and adeno-associated viral vectors [27]. Hence, to achieve efficient delivery of transcriptional units encoding optimized Cas9 nucleases into a broad range of dividing and nondividing target cells, we investigated the suitability of AdVs as delivery vehicles [26–28]. Therefore, we generated CD46-targeted second-generation (i.e., *E1*- and *E2A*-deleted) AdV.Cas9, AdV.eCas9.2NLS and AdV.eCas9.4NLS. We started by transducing HeLa cells with equal doses of these SpCas9-encoding AdVs either alone or in combination with AdV.gRNA^S1^. The proportion between the multiplicity of infections (MOIs) of AdVs expressing SpCas9 nucleases and AdVs expressing gRNAs in this, and subsequent experiments, was 1:1. AdV.gRNA^S1^ is equally a CD46-targeted second-generation AdV that expresses a gRNA addressing SpCas9 nucleases to the human *AAVS1* locus [28]. Immunofluorescence microscopy analysis of SpCas9 expression showed a subcellular distribution reminiscent of that obtained after transfecting plasmids encoding the same RGNs (Figs. 1b and 5a). In particular, unbiased quantification of fluorescence intensities in SpCas9-stained cells revealed higher amounts of antigen in the nuclear compartment over the cytoplasmic compartment in cells exposed to AdV.eCas9.4NLS when compared with those present in these compartments in cells transduced with AdV.Cas9 or AdV.eCas9.2NLS (Fig. 5b, c, Supplementary Figs. 4 and 5a and Supplementary Table 7). This robust effect on the differential distribution of Cas9, eCas9.2NLS and eCas9.4NLS between the nucleus and cytoplasm of transduced cells, was similar in cell cultures incubated with or without AdV.gRNA^S1^ (Fig. 5b, c, Supplementary Figs. 4 and 5a and Supplementary Table 7). Importantly, enrichment in the nuclear compartmentalization of SpCas9 proteins, calculated as the ratio between geometric mean intensity (GMI) in the nucleus versus GMI in the whole cell, increased up to 14-fold by using AdV.eCas9.4NLS (Fig. 5c, Supplementary Fig. 5b and Supplementary Table 7). These data were confirmed by an independent dose-response experiment in which HeLa cells were transduced with different amounts of AdV.eCas9.4NLS mixed with or without AdV.gRNA^S1^ (Supplementary Fig. 6). Indeed, in cells stained positive for nuclease expression, the SpCas9 nuclear enrichment was independent of the MOI applied (Supplementary Fig. 6). These results suggest that the extent of nuclear translocation is an intrinsic property of the nuclease which is independent of its total concentration in the cell. Finally, this quantitative single cell-level fluorescence microscopy analysis in which isogenic constructs eCas9.2NLS and eCas9.4NLS were compared side-by-side, demonstrates a clear direct relationship between the number of NLS motifs and the nuclear localization of the respective proteins (Fig. 5b, c, Supplementary Figs. 4 and 5a).

### Quantification of targeted DNA cleavage induced by AdVs encoding SpCas9 nucleases

**Fig. 6:**
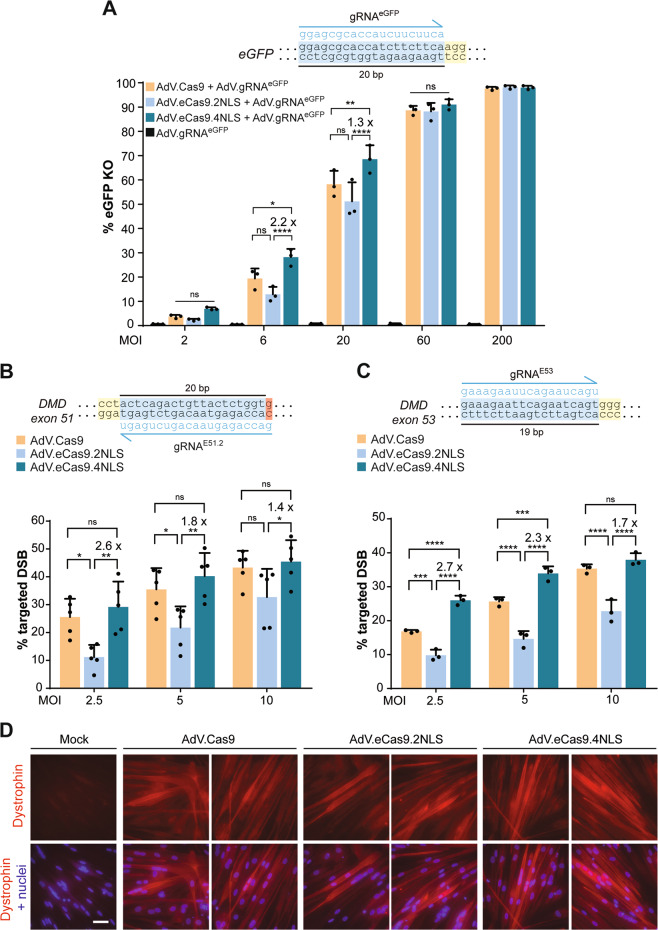
Investigating genome editing induced by AdVs encoding standard versus optimized SpCas9 nucleases. **a** Quantification of *eGFP* knockout frequencies in AdV-transduced H27 cells. Upper panel, *eGFP* target site composed of gRNA-matching sequence (protospacer) and PAM (light blue and yellow boxes, respectively). Lower panel, quantification of target gene knockout by eGFP-directed flow cytometry of H27 cells transduced with the indicated AdVs. The flow cytometry analysis was performed at 10 days post transduction. Bars and error bars indicate mean and SD, respectively, of three independent experiments. At least 10,000 events, each corresponding to a single viable cell, were measured per sample. **b** Quantification of targeted DSB formation in patient-derived DMD.∆48–50^gE51.2^ myoblasts. DMD.∆48–50^gE51.2^ myoblasts constitutively express gE51.2. Upper panel, target site of gE51.2 addressing the Cas9 nucleases to exon 51 of the *DMD* locus. The gRNA-matching sequence (protospacer) and PAM are highlighted by the light blue and yellow boxes, respectively. The 1-nt mismatch between gRNA and target site is highlighted in red. Lower panel, DMD.∆48–50^gE51.2^ myoblasts were transduced with the indicated AdVs at different multiplicities of infection (MOIs). Genomic DNA from DMD.∆48–50^gE51.2^ myoblasts transduced with the indicated AdVs were subjected to T7EI-based genotyping assays at 3 days post transfection. Negative controls are presented in Supplementary Fig. 10. **c** Quantification of targeted DSB formation in patient-derived DMD.∆45–52^gE53^ myoblasts. DMD.∆45–52^gE53^ myoblasts constitutively express gE53. Upper panel, target site of gE53 targeting SpCas9 nucleases to exon 53 of the *DMD* locus. The gRNA-matching sequence (protospacer) and PAM are highlighted by the light blue and yellow boxes, respectively. Lower panel, transduction experiments and targeted DNA cleavage analysis were performed as in the experiments done on DMD.∆48–50^gE51.2^ myoblasts. Negative controls are presented in Supplementary Fig. 11. Bars and error bars indicate mean and SD, respectively, of a minimum of three independent experiments. Significance was calculated with two-way ANOVA followed by Tukey’s test for multiple comparisons: ns non significant, *P* ≥ 0.05; *P < 0.05; **P < 0.01; ***P < 0.001; ****P < 0.0001. MOI multiplicity of infection. **d** Dystrophin immunofluorescence microscopy on *DMD*-edited myotubes. Immunostainings for dystrophin were carried out in myotubes differentiated from DMD.∆48–50^gE51.2^ myoblasts transduced with the indicated AdVs at an MOI of 10 IU/cell. Mock-transduced myoblasts provided for negative controls. The horizontal white bar corresponds to 50 µm.

Functional validation of AdV.eCas9.4NLS was initiated by transducing HeLa cells in combination with AdV.gRNA^S1^. T7EI-based genotyping assays revealed a dose-dependent increase in indel frequencies at *AAVS1* (Supplementary Fig. 7). To accurately quantify differences in targeted DNA cleaving activities induced by AdV.Cas9, AdV.eCas9.2NLS and AdV.eCas9.4NLS, we transduced eGFP-positive H27 cells at different doses together with AdV.gRNA^eGFP^ [28] and determined *eGFP* knockout frequencies by flow cytometry (Fig. 6a and Supplementary Table 8). AdV.gRNA^eGFP^ is a CD46-targeted second-generation AdV that expresses a canonical gRNA addressing SpCas9 nucleases to the *eGFP* reporter (Fig. 6a) [28]. At 10 days post transduction, flow cytometry and direct fluorescence microscopy revealed a clear dose-dependent increase in the fraction of eGFP-negative cells in all cultures exposed to AdV.gRNA^eGFP^ in combination with AdV.Cas9, AdV.eCas9.2NLS or AdV.eCas9.4NLS (Fig. 6a, Supplementary Fig. 8 and Supplementary Table 8). Crucially, at MOIs of 6 and 20 IU/cell, AdV.eCas9.4NLS mediated higher *eGFP* knockout levels when compared with those triggered by AdV.Cas9 or AdV.eCas9.2NLS (Fig. 6a and Supplementary Table 8).

Finally, we set out to investigate the potential of AdVs encoding eCas9.4NLS for genome editing in difficult-to-transfect cells. To this end, we deployed myoblasts isolated from patients with the muscle-wasting disorder Duchenne muscular dystrophy (DMD). In particular, DMD.48–50^gE51.2^ and DMD.45–52^gE53^ myoblasts whose dystrophin-encoding *DMD* gene lacks exons 48 through 50 and exons 45 through 52, respectively [46]. These deletions destroy the *DMD* reading frame, severally reducing dystrophin amounts in striated muscle cells (Supplementary Fig. 9). Furthermore, DMD.48–50^gE51.2^ and DMD.45–52^gE53^ myoblasts express gRNAs targeting SpCas9 nucleases to exon 51 and exon 53, respectively [46]. After AdV-mediated transfer of SpCas9 proteins into DMD.48–50^gE51.2^ and DMD.45–52^gE53^ myoblasts, the resulting RGNs can lead to the NHEJ-mediated restoration of the *DMD* reading frame (Supplementary Fig. 9). Of note, gE51.2 and gE53 are noncanonical gRNAs in that they have a 5′-end-mismatching G and a 19-nt spacer, respectively (Fig. 6b, c).

DMD.48–50^gE51.2^ myoblasts were transduced with AdV.Cas9, AdV.eCas9.2NLS, or AdV.eCas9.4NLS at MOIs of 2.5, 5, and 10 IU/cell. Possibly due to the mismatching G in gE51.2, AdV.Cas9 led to higher indel frequencies at *DMD* exon 51 than AdV.eCas9.2NLS as determined by T7EI-based genotyping assays (Fig. 6b and Supplementary Fig. 10). Indeed, as aforementioned, compared with the conventional Cas9, these gRNA modifications often reduce DNA cleaving activities when coupled to high-specificity SpCas9 variants, including eCas9.2NLS [16, 34–36]. Importantly, however, transduction of DMD.48–50^gE51.2^ myoblasts with AdV.eCas9.4NLS resulted in significantly higher indel frequencies at *DMD* exon 51 than those achieved by AdV.Cas9.2NLS (Fig. 6b and Supplementary Fig. 10).

The superior performance of AdV.eCas9.4NLS over AdV.eCas9.2NLS was confirmed in independent experiments targeting *DMD* exon 53 in DMD.48–50^gE53^ myoblasts (Fig. 6c and Supplementary Fig. 11). In addition, in DMD.48–50^gE53^ myoblasts exposed to the two lowest doses of AdVs, the DSB formation levels achieved by AdV.eCas9.4NLS were significantly higher than those induced by AdV.Cas9 as well (Fig. 6c and Supplementary Fig. 11). It is also noteworthy mentioning that fourfold higher doses of AdV.eCas9.2NLS were necessary to reach the targeted DSB formation frequencies achieved by AdV.eCas9.4NLS at both *DMD* target sites (Fig. 6b, c). In particular, transduction of human myoblasts with AdV.eCas9.4NLS and AdV.eCas9.2NLS at MOIs of 2.5 and 10 IU/cell, respectively, led to similar frequencies of targeted DSB formation (Fig. 6b, c). These data are relevant in that it opens the perspective for achieving efficient targeted DNA cleavage with substantially lower doses of AdV particles.

Finally, we complemented the *DMD* gene-editing analyses by performing dystrophin immunofluorescence microscopy on DMD.48–50^gE51.2^ myotubes differentiated from myoblasts transduced with AdVs encoding the different nucleases (Fig. 6d). The clear detection of dystrophin protein in *DMD-*edited myotubes established that combining AdV-mediated gene delivery with optimized SpCas9 nucleases constitute a promising approach for carrying out genome editing in difficult-to-transfect cells (Fig. 6d).

## Discussion

Delivering programmable nucleases into target cell nuclei remains a major challenge for broadening the applicability of RGN-assisted genome editing. Although linking large prokaryotic Cas9 proteins (~160 kDa) to NLS motifs is commonly used to drive their nuclear translocation in eukaryotic cells, so far, Cas9 proteins and their variants have been mostly linked to either one or two NLSs [5–7, 17, 47]. In addition, an unbiased quantification of nuclear translocation levels and a thorough assessment of the impact of NLS composition on RGN performance had not been done. To address this, we investigated the effects on nuclear localization and target DNA cleavage of SpCas9 nucleases containing distinct NLS compositions. In these experiments, we deployed a monopartite NLS peptide (PKKKRKV) identified in the SV40 large T antigen [48, 49] and a bipartite NLS (KRPAATKKAGQAKKKK) found in the *Xenopus* sp. protein nucleoplasmin [50]. These NLS motifs engage the classical NLS-mediated nuclear transport pathway. In brief, SV40 large T antigen and nucleoplasmin NLSs bind to the cellular importin-α that, after dimerization with importin-β, mediate the translocation of the attached cargo from the cytoplasm to the nucleus through nuclear pore complexes [24, 25]. In this study, quantitative and unbiased immunofluorescence microscopy assays demonstrated that the eCas9.4NLS nuclease accumulates 2.3-fold more in the nucleus than its parental eCas9.2NLS protein [17].

The experiments performed in HEK.EGFP^TetO.KRAB^ reporter cells [34, 40, 45] allowed us probing the performance of SpCas9 proteins with different NLS arrangements at target DNA subjected to alternative chromatin conformations. The resulting data suggest that RGNs with eCas9.2NLS or eCas9.4NLS engage with DNA embedded in KRAB-impinged heterochromatin better than Cas9. It will be valuable investigating whether this trend simply results from increased SpCas9 nuclease concentrations in target cell nuclei and/or from local changes in DNA-histone association conferred by the high cationic character of the SpCas9-linked NLS motifs.

We have also validated new combinations of RGN components by demonstrating that high-specificity eCas9.2NLS and eCas9.4NLS nucleases are compatible with opt-gRNAs. Importantly, after implementing quantitative off-target detection systems, we gathered data demonstrating that enhanced RGNs, consisting of opt-gRNAs coupled to eCas9.2NLS or eCas9.4NLS, retain DNA cleaving specificities that are higher than that of their Cas9 counterpart.

A potential limitation of constructs encoding SpCas9 nucleases endowed with various heterologous motifs (e.g., NLSs) and fairly large regulatory elements (e.g., CAG promoter) is that of their delivery into difficult-to-transfect cells. To overcome this hurdle, we generated AdVs encoding Cas9, eCas9.2NLS or eCas9.4NLS. Moreover, we took advantage of the AdV transduction mechanisms to introduce well-defined amounts of different SpCas9 proteins into human cells. We carried out these experiments in eGFP-positive HeLa cells and myoblasts isolated from patients with the X-linked muscle-wasting disorder DMD. The eGFP-positive HeLa cells served as a loss-of-function cellular system that allowed for determining gene knockout levels triggered by AdVs encoding Cas9, eCas9.2NLS or eCas9.4NLS. Importantly, exposing target cells to moderate doses of AdV.eCas9.4NLS led to higher *eGFP* knockout levels than those achieved by using the same doses of AdV.Cas9 or AdV.eCas9.2NLS. The DMD patient-derived myoblasts were instead used as a gain-of-function cellular system based on difficult-to-transfect cells. In this system, RGNs were used to trigger NHEJ-mediated repair of defective *DMD* reading frames. In these experiments, we found that at matched AdV doses, eCas9.4NLS outperformed eCas9.2NLS, thus allowing robust *DMD* reading frame correction. The improved performance of AdV.eCas9.4NLS is expected to be particularly advantageous in experimental settings that require minimizing the exposure of target cells to viral vector components and delivered gene-editing tools.

NLSs have been linked not only to RGNs but also to other types of programmable nucleases, such as zinc finger nucleases and transcription activator-like effector nucleases [51–55]. Of note, when delivering programmable nucleases directly as proteins, NLSs, due to their net positive charge, seem to operate as protein transduction domains hence aiding in cell entry [52, 54, 55]. Thus, these studies could not establish in a strict manner the contribution of NLSs to the nuclear translocation per se due to their simultaneous involvement in cell penetration of programmable nucleases [52, 54, 55]. In the present work, by using AdV-mediated expression of different NLS-linked SpCas9 nucleases we were able to strictly demonstrate that Cas9 engineering profits from including a higher number of NLS sequences than those present in currently used Cas9 nucleases. The impact that delivery methods or experimental settings might have on the activity of Cas9 nucleases endowed with different NLS arrangements and numbers is illustrated in recent studies [56, 57]. For instance, Torres-Ruiz et al. used plasmid transfections to introduce into human cells RGNs containing Cas9 nucleases with a single SV40 NLS at the C-terminus or with this NLS at the C-terminus and a nucleoplasmin NLS at the N-terminus [57]. These experiments did show an enhanced nuclear uptake and DNA cleaving activity by the 2NLS-containing protein in HEK293 cells and human mesenchymal stem cells [57]. Our side-by-side testing of a high-specificity Cas9 variant equally flanked by the SV40 and nucleoplasmin NLSs (i.e., eCas9.2NLS) and its derivative endowed with two extra SV40 NLSs (i.e., eCas9.4NLS) revealed that the latter construct presents superior nuclear uptake and targeted DNA cleaving activities while retaining for the most part the high specificity of its parental, eCas9.2NLS, protein.

Taken these data together, we conclude that designing SpCas9 proteins with extra NLSs is a straightforward and effective strategy to enhance the performance of RGNs and raise the possibility for further improvements via additional optimization of their NLS content. Finally, the testing and use of the resulting optimized gene-editing tools in genetic therapies will profit from their integration with viral vector systems such as the herein applied CD46-targeting AdV platform. This gene delivery platform should permit stoichiometric “all-in-one” delivery of optimized RGN components into therapeutically relevant cell types.

## Materials and methods

### Cells

The human cervix carcinoma HeLa cells (American Type Culture Collection) were cultured in Dulbecco’s modified Eagle’s medium containing 5% fetal bovine serum (FBS), at 37 °C in a 10% CO_2_ atmosphere. The origin of and culture conditions for the HeLa cell-derived clone H27 and H11.7, constitutively expressing eGFP and mTurquoise2, respectively, have been detailed elsewhere [33, 37]. The generation of and the culture conditions for the human embryonic kidney cell-derived cells HEK.EGFP^TetO.KRAB^ were also detailed elsewhere [40]. The *E1*- and *E2A*-complementing AdV packaging cell line PER.E2A was cultured as previously described [28, 58].

Human myoblasts DMD.Δ48–50^gE51.2^ and DMD.45–52^gE53^ were generated and cultured as described in detail elsewhere [46]. DMD.Δ48–50^gE51.2^ myoblasts harbor *DMD* intragenic deletions spanning exons 48 to 50 and express gRNA gE51.2. DMD.45–52^gE53^ myoblasts have *DMD* multiexon deletions spanning exon 45 to 52 and express gRNA gE53. The gE51.2 and gE53 expression units were delivered into the DMD myoblasts by lentiviral vectors encoding a BSD::EGFP marker. After blasticidin selection, the resulting DMD myoblast populations expressed the respective gRNAs [46]. The cells used in this study were mycoplasma free.

### Plasmids

The isogenic constructs AV62_pCAG.Cas9.rBGpA and AW01_pCAG.eSpCas9(1.1).rBGpA, encoding Cas9 and eCas9.2NLS, respectively, were previously described [40]. The former and latter construct harbor, respectively, codon-optimized ORFs coding for the *S. pyogenes* proteins SpCas9 [5], herein dubbed Cas9, and eSpCas9(1.1) [17], herein named eCas9.2NLS. The plasmid encoding eCas9.4NLS was constructed by inserting two NLSs from the SV40 large T antigen, downstream of the ORF coding for eCas9.2NLS. The annotated map and nt sequence of the eCas9.4NLS-encoding plasmid are presented in Supplementary Fig. 12. The annotated amino acid sequence of the eCas9.4NLS protein is depicted in Supplementary Fig. 13.

The gRNA acceptor plasmid with the opt-gRNA scaffold AY56_pUC.U6.opt-sgRNA.BveI-stuffer was made by ligating annealed oligonucleotides 5′- GGCCGCACCTGCTGACGTTTCAGAGCTATGCTGGAAACAGCATAGCAAGTTGAAATAAGGCTAGTCCGTTATCAACTTGAAAAAGTGGCACCGAGTCGGTGCTTTTTTTG-3′ and 5′- AATTCAAAAAAAGCACCGACTCGGTGCCACTTTTTCAAGTTGATAACGGACTAGCCTTATTTCAACTTGCTATGCTGTTTCCAGCATAGCTCTGAAACGTCAGCAGGTGC -3′ into the NotI/EcoRI-digested construct S7_pUC.U6.sgRNA.BveI-stuffer [40]. The gRNA expressing plasmids harboring standard or opt-gRNA scaffolds were made by ligating a pair of annealed oligonucleotides into the BveI-digested acceptor construct S7_pUC.U6.sgRNA.BveI-stuffer or AY56_pUC.U6.opt-sgRNA.BveI-stuffer, respectively. The oligonucleotide pairs used to generate gRNA expression plasmids addressing the Cas9 nuclease to the *eGFP.3*, *eGFP.5*, *eGFP.6*, *eGFP.16*, and *AAVS1* target sites are listed in Supplementary Table 9. The plasmids expressing opt-gRNAs containing different numbers and positions of nt mismatches to a *eGFP* target site (opt-gOT.1 through opt-gOT.10) were assembled by inserting a pair of annealed oligonucleotides into the BveI-treated acceptor construct AY56_pUC.U6.opt-sgRNA.BveI-stuffer. The oligonucleotide pairs used to generate this set of reagents are listed in Supplementary Table 10.

The constructs gRNA_GFP_T2 (Addgene #41820) and AV59_pg9.20, herein named gRNA^eGFP.8^ and gRNA^eGFP.9^, respectively, were previously described [34, 38]. The construct pgRNA (Addgene #41824), herein named gRNA^empty^, expresses no gRNA and was used as a negative control wherever indicated. The expression of all gRNAs used in this study are under the control of the human *U6* promoter whose preferred transcript initiating nt is a G.

The expression plasmid AM37_pCMV.DsRedEx2.1.bGHpA (DsRed in short) was used as a control for determining the transfection efficiencies in experiments performed in the reporter cells HEK.EGFP^TetO.KRAB^ [34] and H27 for determining chromatin impact indexes and specificity profiles of Cas9 nucleases, respectively.

To generate the AdV shuttle plasmid AY37_pAd.Shu.CAG.Cas9^eSp(1.1)^.rBGpA and AY38_pAd.Shu.CAG.Cas9^eSp(1.1)^.rBGpA.4NLS, the constructs AW01_pCAG.eSpCas9(1.1).rBGpA and eCas9.4NLS were digested with MssI (Thermo Fisher Scientific) and the resulting fragments containing the nuclease expression units were subsequently isolated and ligated into the ScaI restriction sites of AQ08_pAd.Shu.MCS. The map and nt sequence of AQ08_pAd.Shu.MCS plasmid are presented in Supplementary Fig. 14.

Next, the full-length *E1-* and *E2A-*deleted, i.e., second-generation, fiber-modified AdV molecular clones AY39_pAd.CAG.Cas9^eSp(1.1)^.rBGpA.ΔE2A.F^50^ and AY40_pAd.CAG.Cas9^eSp(1.1)^.rBGpA.4NLS.ΔE2A.F^50^ were assembled via homologous recombination (HR) after the transformation of *E. coli* cells BJ5183^pAdEasy-2.50^ [59] with the MssI-treated AY37_pAd.Shu.CAG.Cas9^eSp(1.1)^.rBGpA and AY38_pAd.Shu.CAG.Cas9^eSp(1.1)^.rBGpA.4NLS, respectively. The assembly of full-length AdV molecular clones in these bacteria has been detailed elsewhere [59]. Similarly, the full-length *E1-* and *E2A-*deleted fiber-modified AdV molecular clone AU59_pAd.CAG.Cas9.SV40pA.ΔE2A.F^50^ was assembled via HR after the transformation of *E. coli* cells BJ5183^pAdEasy-2.50^ [59] with the MssI-treated AdV shuttle plasmid AU51_pAd.shu.CAG.Cas9.SV40pA. The map and nt sequence of AU51_pAd.shu.CAG.Cas9.SV40pA are presented in Supplementary Fig. 15.

All plasmids were purified with LabNed Plasmid Maxiprep Kit (ImTec Diagnostics) according to the manufacturer’s recommendations.

### Production and characterization of AdVs

The constructs AU59_pAd.CAG.Cas9.SV40pA.ΔE2A.F^50^, AY39_pAd.CAG.Cas9^eSp(1.1)^.rBGpA.ΔE2A.F^50^, and AY40_pAd.CAG.Cas9^eSp(1.1)^.rBGpA.4NLS.ΔE2A.F^50^ were used for the production of the fiber-modified, *E1-* and *E2A-*deleted AdVs AdV.Cas9, AdV.eCas9.2NLS and AdV.eCas9.4NLS, respectively. The protocols used in the generation, purification, and titration of the resulting AdV stocks have been described in detail before [28, 60]. The titers of purified AdV.Cas9, AdV.eCas9.2NLS, and AdV.eCas9.4NLS stocks were 2.37 × 10^10^ IU/ml, 13.3 × 10^10^ IU/ml, and 3.29 × 10^10^ IU/ml, respectively. The generation and characterization of the fiber-modified, *E1-* and *E2A-*deleted, AdVs AdV^Δ2^U6.gRNA^S1^.F^50^ and AdV^Δ2^U6.gRNA^GFP^.F^50^, herein named AdV.gRNA^S1^ and AdV.gRNA^GFP^, respectively, has been described previously [28].

### Transfection experiments

Plasmid transfections for introducing RGN components into human cervix carcinoma cells were initiated by seeding cells in wells of 24-well plates (Greiner Bio-One). HeLa, H27, and H11.7 cells were seeded at a density of 3–4 × 10^4^ cells per well. 24 h after seeding, the cells were transfected with DNA mixtures consisting of 250 ng of plasmids encoding Cas9 protein and 250 ng of plasmids expressing gRNAs. These DNA mixtures were diluted in 50 μl of 150 mM NaCl (Merck), supplemented with 2.19 μl of a 1 mg/ml polyethylenimine (PEI) solution and directly subjected to vigorous vortexing for about 10 s. After a 15-min incubation at room temperature, the resulting polycation–DNA complexes were directly added to the culture medium of the test cells. After a 6-h incubation, the transfection mixtures were replaced with fresh culture medium. At 3 days post transfection, total cellular DNA from HeLa cells was isolated by using the DNeasy Blood & Tissue kit (Qiagen) following the manufacturer's recommendations. Cultures with transfected H27 and H11.7 cells were instead subcultured for 10 days, at which time point, reporter-negative cells were quantified by flow cytometry as described below.

Transfection experiments for comparing the specificity of Cas9, eCas9.2NLS and eCas9.4NLS in H27 cells were done following the scheme provided in Supplementary Table 11 using the PEI-based protocol described in the first paragraph. The expression plasmid AM37_pCMV.DsRedEx2.1.bGHpA [34] was included in each DNA mixture to determine transfection efficiencies at 3 days post transfection. The construct AM51_pgNT [45] provided for a *eGFP* nontargeting gRNA (gNT) control. The frequencies of *eGFP* knockout were determined by flow cytometry at 10 days post transfection and were then normalized for the initial transfection efficiencies.

Transfection experiments in HEK.EGFP^TetO.KRAB^ cells were carried out as detailed elsewhere [40]. In brief, after exposing HEK.EGFP^TetO.KRAB^ cells to regular growth medium or to medium supplemented with Dox (500 ng/ml) for 7–10 days, cells were seeded at a density of 1.5 × 10^5^ cells per well in 24-well plates. The next day, the cells were transfected according to the scheme presented in Supplementary Table 12 using the PEI-based protocol specified in the first paragraph. The expression plasmid AM37_pCMV.DsRedEx2.1.bGHpA was included in the transfection mixtures to evaluate transfection efficiencies by DsRed-direct flow cytometry at 3 days post transfection. Next, the transfected cell cultures were subcultured every 3–4 days for a period of 7 days. In order to activate target transgene expression, at 10 days post transfection HEK.EGFP^TetO.KRAB^ cells that had not been initially exposed to Dox were subcultured in medium supplemented with Dox (500 ng/ml) for an additional period of 7 days, after which reporter-directed flow cytometry was used to quantify the *eGFP* knockout frequencies.

### Transduction experiments

In all experiments that involved cotransductions for introducing RGN components into target cells, the proportion between the MOIs of AdVs encoding SpCas9 proteins and AdVs encoding gRNAs was 1:1. Transduction experiments in H27 cells were initiated by seeding the cells in wells of 24-well plates at a density of 4 × 10^4^ cells per well. The next day, the cells were incubated in 500 μl of medium containing AdV particles at the indicated MOIs. At 3 days post transduction inocula were discarded and the cells were subcultured for an additional 7 days. At 10 days post transduction, the presence of eGFP-negative cells was assessed by direct fluorescence microscopy and flow cytometry as described below.

Transduction experiments in HeLa cells were performed as in H27 cells, except that at 3 days post transduction, the cell cultures were transferred to Millicell EZ SLIDE glass slides (Merck) for performing Xcyto 10 measurements or were harvested for total cellular DNA isolation by using the DNeasy Blood & Tissue kit (Qiagen).

Gene-editing experiments in DMD.∆48–50^gE51.2^ and DMD.45–52^gE53^ myoblasts were essentially performed as described elsewhere [46]. In brief, DMD.∆48–50^gE51.2^ and DMD.45–52^gE53^ myoblasts were seeded at a density of 3 × 10^4^ cells per well in 24-well plates (Greiner Bio-One), precoated with a 0.1% (w/v) gelatin solution (Sigma-Aldrich). After 24 h, the cells were incubated in 500 µl of medium containing the different amounts of AdV particles. At 3 days post transduction genomic DNA was isolated for assessing the frequencies of DSB formation by T7EI-based genotyping assays (see below) and parallel DMD.∆48–50^gE51.2^ myoblast cultures were exposed to mitogen-poor conditions for triggering myogenic differentiation and myotube formation. After 4 days, the myoblasts were stained for dystrophin (see below).

### T7EI-based genotyping assays

The frequencies of indels resulting from targeted DSB formation at the *AAVS1* and *DMD* loci were assessed as follows. Genomic DNA extracted from transfected or transduced target cells was subjected to PCR for amplifying DNA segments spanning the RGN target sites located in *AAVS1* and *DMD* loci. Next, the resulting amplicons were treated with T7EI (New England Biolabs) in the buffer provided by the manufacturer. The primer sequences, the PCR reagents, the cycling parameters and the protocol for the T7EI-based genotyping assays have all been described before [46]. After densitometry of undigested and T7EI-digested amplicons, indel frequencies were calculated by applying the following formula: 100 × [1 − (1 − fraction cleaved)^1/2^] [61].

### Flow cytometry

The frequencies of eGFP-negative and mTurquoise2-negative cells in H27 and H11.7 cell cultures, respectively, were determined by using a BD LSR II flow cytometer (BD Biosciences). In addition, DsRed-directed flow cytometry was used for determining transfection efficiencies in HEK.EGFP^TetO.KRAB^ reporter cells. At least 10,000 viable single cells were analyzed per experimental condition. Data were analyzed with the aid of FlowJo 10.4 software (Tree Star). Mock-transduced cells served to establish the cutoff between reporter-positive and reporter-negative cell populations.

### Direct fluorescence microscopy

Targeted *eGFP* knockout in AdV-transduced H27 cell cultures was monitored by direct fluorescence microscopy. The H27 cell nuclei were stained by adding 10 μg/ml Hoechst 33342 (Molecular Probes) for 10 min. After washing twice with phosphate-buffered saline (PBS), pH 7.4, culture medium was added to the cell cultures. The eGFP- and Hoechst 33342-specific signals were detected by using an AF6000 LX system and LAS AF software version 2.7.4.10100 (both from Leica).

### Assessment of Cas9 nuclear expression with Xcyto 10

HeLa cells were transfected or transduced as indicated above. Three days later, HeLa cells were seeded in Millicell EZ SLIDE glass (8-well or 4-well, Merck) and were kept in regular growth medium. After overnight incubation at 37 °C in a 10% CO_2_ atmosphere, the HeLa cells were fixed with 4% (w/v) paraformaldehyde in PBS for 15 min at 4 °C. After removing the fixative by two washes with PBS, the cells were permeabilized by adding 0.25% (v/v) Triton X-100 (Sigma-Aldrich) in PBS for 10 min at room temperature. The detergent was removed by three 5-min washes with TBST consisting of 0.1 M Tris-HCl (pH 7.5), 0.15 M NaCl, and 0.05% Tween-20 (Merck). Next, a blocking step was performed by incubating the cells at room temperature for 10 min with TNB containing 0.1 M Tris-HCl (pH 7.5), 0.15 M NaCl, and 0.5% blocking reagent for nucleic acid hybridization and detection (Roche). A mouse monoclonal IgG1 antibody directed against the SpCas9 protein (ab191468; Abcam) was diluted 1:200 in TNB and was incubated with the cells for 1 h at room temperature. Next, the cells were rinsed by three 5-min washes with TBST. The cells were then incubated for 1 h at room temperature in the dark with an Alexa Fluor 488 anti-Mouse IgG (H + L; A11001 Invitrogen) diluted 1:500 in TNB. Excess of secondary antibody was removed by three 5-min washes with TBST. After washing once with PBS, the cells were stained with BlueMask-1 (Solutions 12 + 21, Chemometec) diluted 1:1000 in PBS for 30 min in the dark. After removing the Bluemask-1 dye via four washes with PBS, the chambers were disassembled, gently washed in distilled water, and let to air dry. Glass slides were embedded in 70 µl of Prolong Gold with DAPI (Thermo Fisher Scientific) and were sealed with a glass coverslip. Digital images were acquired by using Xcyto 10 Quantitative Cell Imager (Chemometec) with automated segmentation done by XcytoView 1.0.63.0 software based on the DAPI and BlueMask-1 staining. Finally, the data were analyzed with the aid of FlowJo 10.4 software (Tree Star).

### Immunofluorescence microscopy

HeLa cells were transfected as indicated in “Transfection experiments” section. Three days post transfection, HeLa cells were fixed with 4% (w/v) paraformaldehyde in PBS for 30 min at room temperature. Next, the HeLa cells were stained as described elsewhere [46], except for the following variations. A mouse monoclonal IgG1 antibody directed against the SpCas9 protein (ab191468; Abcam) was diluted 1:200 in 10 mM glycine in PBS (PBSG) with 5% FBS and was then added to the cells for overnight incubation at 4 °C. Subsequently, the cells were stained with an Alexa Fluor 488 anti-Mouse IgG (H + L; A11001 Invitrogen) diluted 1:500 in PBSG containing 5% FBS. Digital images were acquired by using an AF6000 LX system and LAS AF software version 2.7.4.10100 (both from Leica).

DMD.∆48–50^gE51.2^ myoblasts were transduced as described in “Transduction experiments” section. Three days post transduction, cell populations were exposed to mitogen-poor conditions for triggering myotube differentiation. The composition of the differentiation medium is specified elsewhere [46]. After 4 days, the resulting myotubes were fixed with 4% (w/v) paraformaldehyde in PBS for 10 min at 4 °C and were permeabilized by adding 0.5% (v/v) Triton X-100 in TBS (0.05 M Tris-HCl, pH 7.5 and 0.1 M NaCl) for 10 min at room temperature. After three 5-min washes with TBST, a blocking step was performed by incubating the cells for ∼2 h in TBS supplemented with 0.1% (v/v) Triton X-100, 2% (w/v) BSA (Sigma-Aldrich) and 0.1% azide (Merck) at room temperature. A rabbit polyclonal antibody specific for the C-terminus of dystrophin (ab15277; Abcam) was diluted 1:100 in TBS supplemented with 0.1% (v/v) Triton X-100, 2% (w/v) BSA and 0.1% azide. After removing the blocking solution, the cells were incubated overnight at 4 °C with the antibody. Next, the primary antibody was removed by three 5-min washes with TBST and the secondary antibody Alexa Fluor 568 anti-rabbit IgG (H + L; A11036 Invitrogen) was added to the cells diluted 1:500 in TBS supplemented with 0.1% (v/v) Triton X-100, 2% (w/v) BSA and 0.1% azide for 45 min at room temperature. After three 5-min washes with TBST, the cells were incubate with Hoechst 33342 (Molecular Probes) diluted 1:1000 in PBS for 10 min at room temperature. The DNA dye was subsequently removed by three washes with PBS. Digital images were acquired with AF6000 LX system and LAS AF software version 2.7.4.10100 (both from Leica).

### Statistical analyses

Data derived from a minimum of three independent biological replicates were analyzed by using GraphPad Prism 8.0.1 software. Statistical significance was calculated with the tests indicated in the various figure legends. *P* values lower than 0.05 were considered to be statistically significant. No statistical methods were used to predefine sample size and no data were excluded from the analyses. There was no use of randomization methods for sample allocation to experimental groups and the investigators were not blinded to group allocation. The variance, including SD and SEM values, resulting from statistically compared experimental groups were similar.

## Supplementary information

Supplementary Information

## Data Availability

All relevant results generated in this study are available within the paper and accompanying Supplementary Information.
