# Supplementary material for: Integrating gene delivery and gene-editing technologies by adenoviral vector transfer of optimized CRISPR-Cas9 components
Source: Gene Ther. 2020 Jan 3;27(5):209–25. doi: 10.1038/s41434-019-0119-y (PMC7253353; doi:10.1038/s41434-019-0119-y)
Supplement: Supplementary file 1 — Supplementary Information [file 41434_2019_119_MOESM1_ESM.docx]

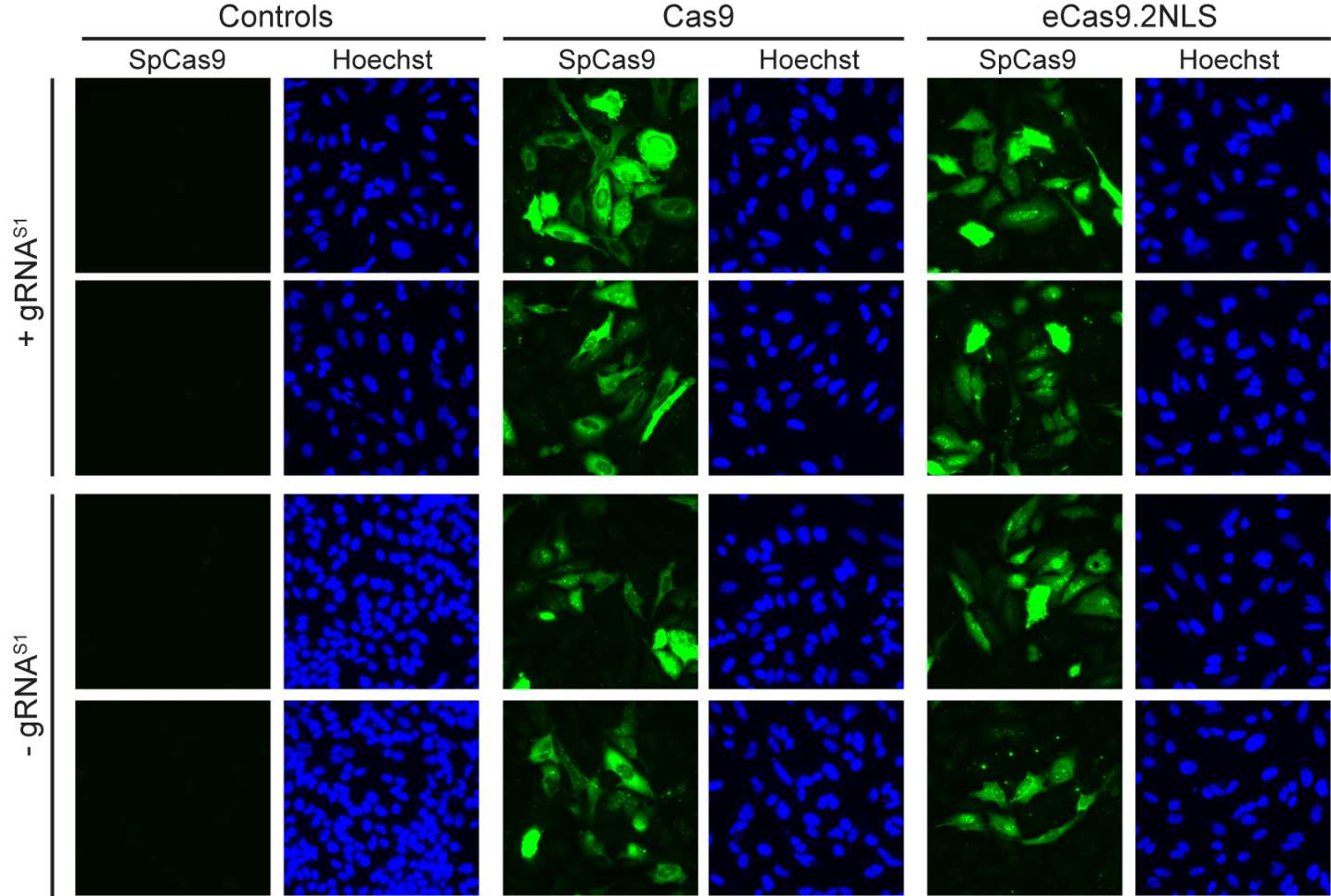


Supplementary Fig. 1 SpCas9 immunofluorescence microscopy on transfected HeLa cells.

HeLa cells were transfected with constructs expressing the indicated RGN components. Fluorescence microscopy images were acquired at 3 days post-transfection. The staining for SpCas9 proteins and DNA was performed with an antibody specific for the C-terminus of SpCas9 and Hoechst 33342, respectively.

**
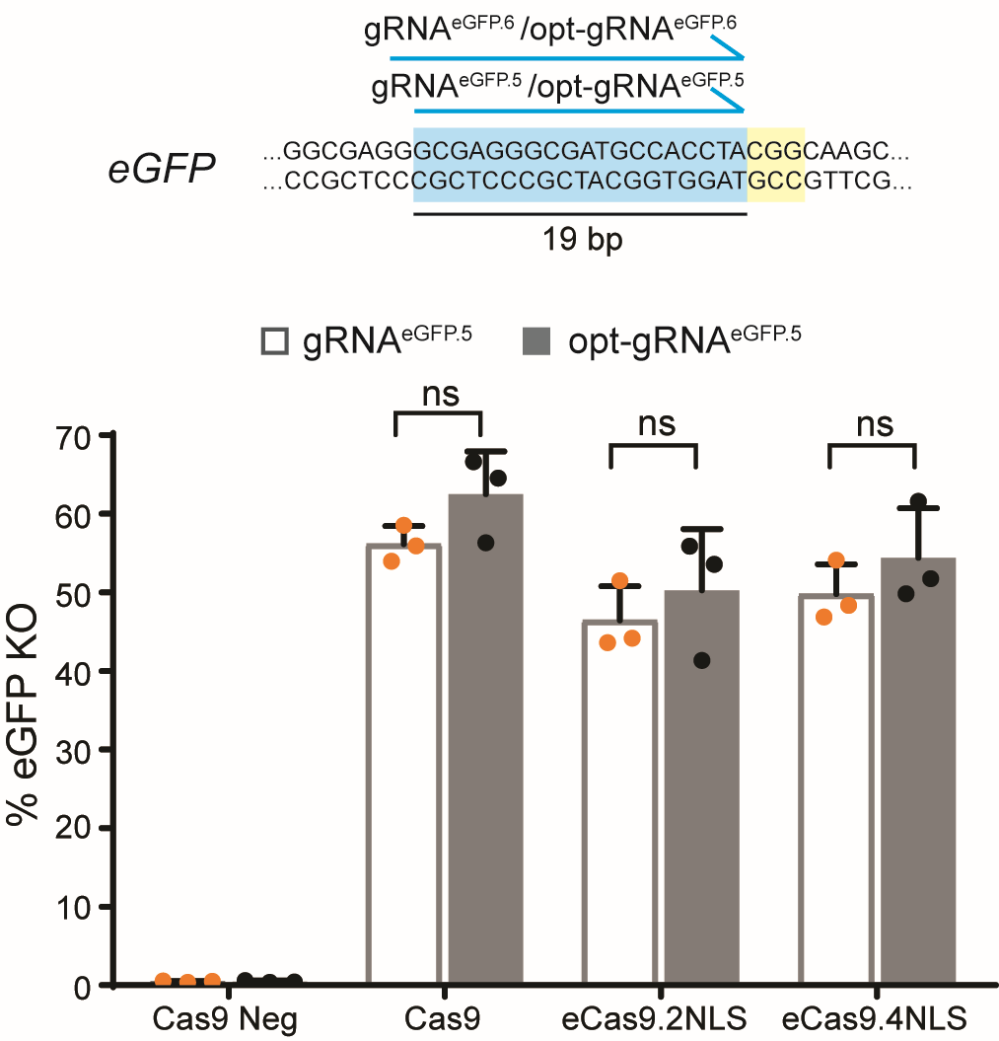
**

Supplementary Fig. 2 Target gene knockout frequencies in H27 cells exposed to the indicated RGN components.

Upper panel, schematic representation of the gRNA^eGFP.5^ and opt-gRNA^eGFP.5^ target site consisting of a 19-nt *eGFP* matching sequence (protospacer) and PAM (light blue and yellow boxes, respectively). gRNA^eGFP.5^ and opt-gRNA^eGFP.5^ are depicted in relation to gRNA^eGFP.6^ and opt-gRNA^eGFP.6^ used in the experiments presented in Fig. 3B. Lower panel, *eGFP* disruption frequencies determined by flow cytometry of H27 cells transfected with the indicated constructs at 10 days post-transfection. Bars and error bars indicate mean and SD, respectively, of three independent experiments. Data points are presented in Supplementary Table 2. At least 10,000 events, each corresponding to a single viable cell, were measured per sample. Significance was calculated with repeated measures one-way ANOVA followed by Tukey’s test for multiple comparisons: ns, non-significant.


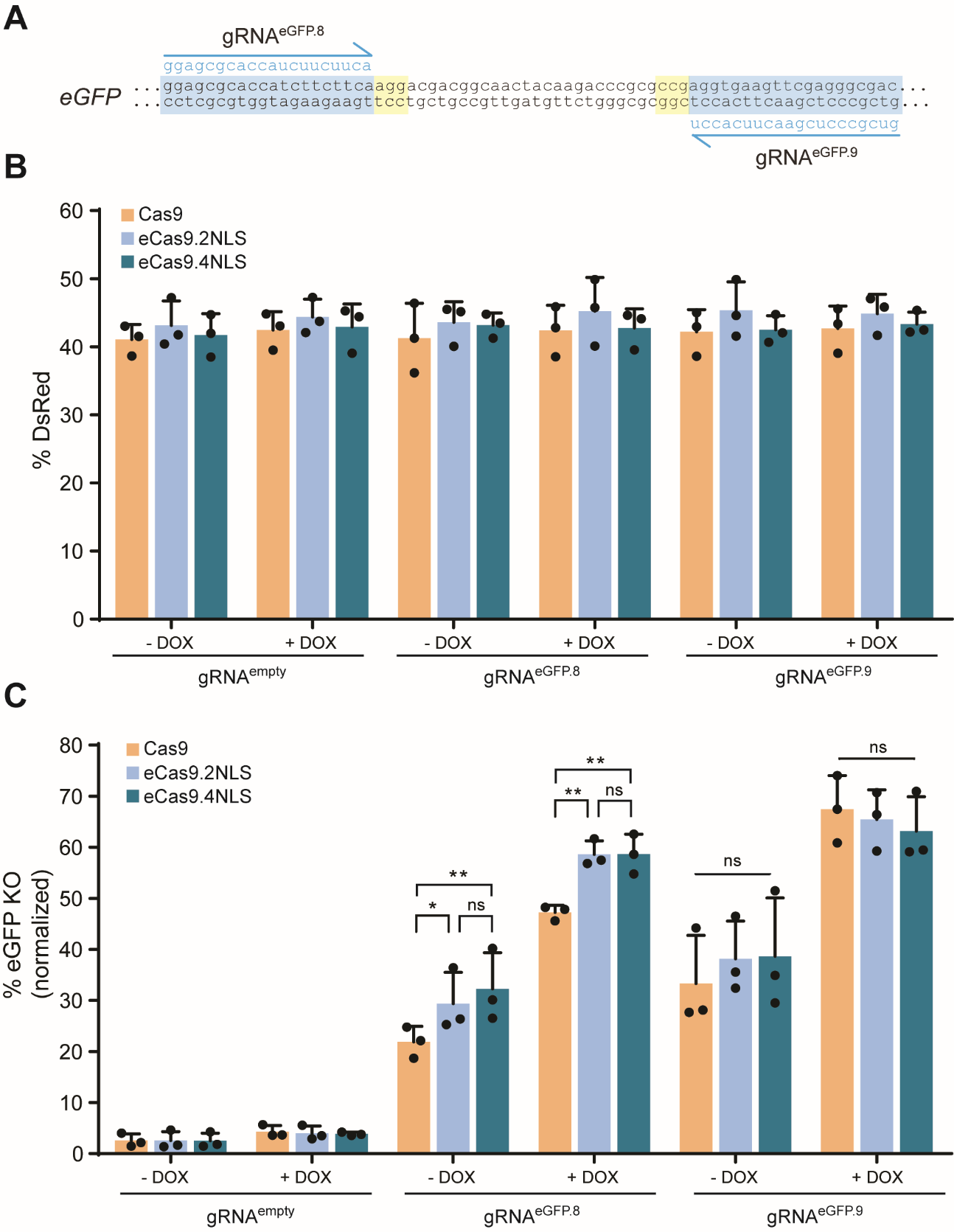


Supplementary Fig. 3 Investigating the impact of alternative chromatin structures on RGNs containing Cas9, eCas9.2NLS or eCas9.4NLS.

(a) Target site sequence of gRNA^eGFP.8^ and gRNA^eGFP.9^ in the *eGFP* locus. gRNA-matching sequences (protospacers) and PAMs are highlighted in light blue and yellow boxes, respectively. (b) Transfection efficiencies in HEK.EGFP^TetO.KRAB^ reporter cells exposed to conventional or optimized SpCas9 nucleases. The transfection efficiencies in HEK.EGFP^TetO.KRAB^ cells were evaluated by including as internal control a DsRed expression plasmid in the various DNA mixtures. The percentage of DsRed-positive HEK.EGFP^TetO.KRAB^ cells were determined by flow cytometry at 3 days post-transfection. Data points are presented in Supplementary Table 4. At least 10,000 events, each corresponding to a single viable cell, were measured per sample. Bars and error bars indicate mean and SD, respectively, of three independent experiments. Differences between experimental conditions were not statistically significative (P > 0.05) as assessed by two-way ANOVA followed by Tukey’s test for multiple comparisons. (c) Gene knockout frequencies at euchromatin versus heterochromatin. HEK.EGFP^TetO.KRAB^ cells, incubated in the presence or absence of Dox, were transfected with the indicated RGN components. Targeted DSB formation at euchromatic (+ Dox) and heterochromatic (- Dox) isogenic target sequences was quantified by eGFP-directed flow cytometry. Data points are presented in Supplementary Table 5. Bars and error bars indicate mean and SD, respectively, of three independent experiments. At least 10,000 events, each corresponding to a single viable cell, were measured per sample. Significance was calculated with two-way ANOVA followed by Tukey’s test for multiple comparisons. ns, non significant; **P* <0.05; ***P*<0.01; ****P*<0.001; *****P*<0.0001.





**Supplementary Fig. 4** Ratios of mean fluorescence intensities between SpCas9-specific signals in nuclei versus cytoplasm. Ratios of the mean fluorescence intensity (MFI) values in the nucleus versus cytoplasm of AdV-transduced cells calculated from the MFI values plotted in Fig. 5B. Significance between the indicated datasets was determined by two-tailed Student’s *t* tests.


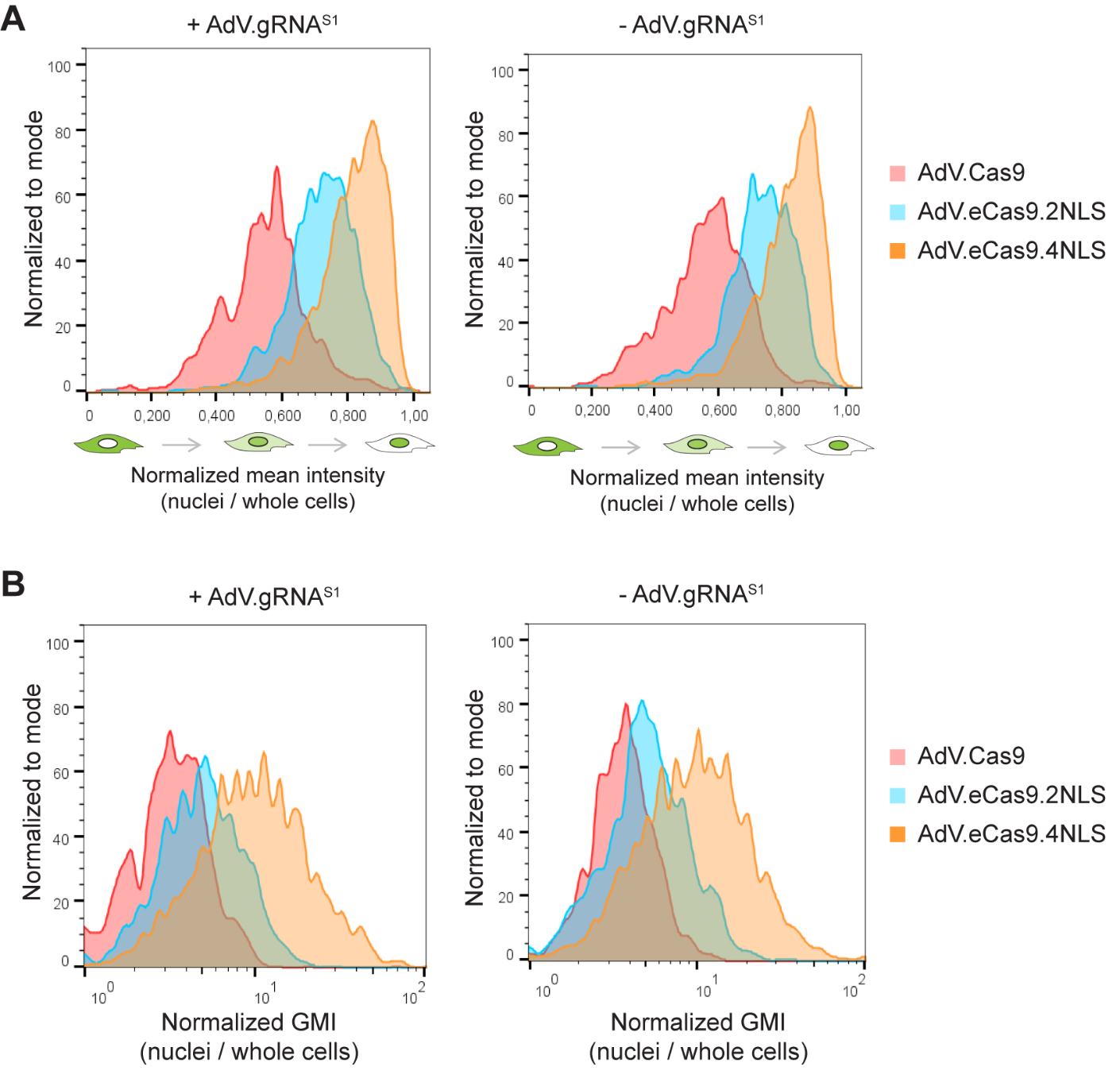


Supplementary Fig. 5 Cumulative histograms corresponding to the relative nuclear enrichment of SpCas9 nucleases in AdV-transduced cells.

(**a**) AdV-transduced HeLa cells were stained for SpCas9 nucleases. Histograms indicate the distribution of normalized fluorescent intensity of nuclei in cells positive for nuclease expression. All data points were acquired from three independent experiments. (**b**) Histograms indicate the distribution of normalized geometric mean intensity (GMI) of the nuclei in cell populations positive for Cas9 expression. The GMI values from the nuclei were normalized to the GMI values from the whole cells. The histograms are generated by plotting all data points generated from three independent experiment. Bar charts are presented in Fig. 5C. Data were acquired with Xcyto 10 using a 20× magnification. Automated cell segmentation was performed with XcytoView software.


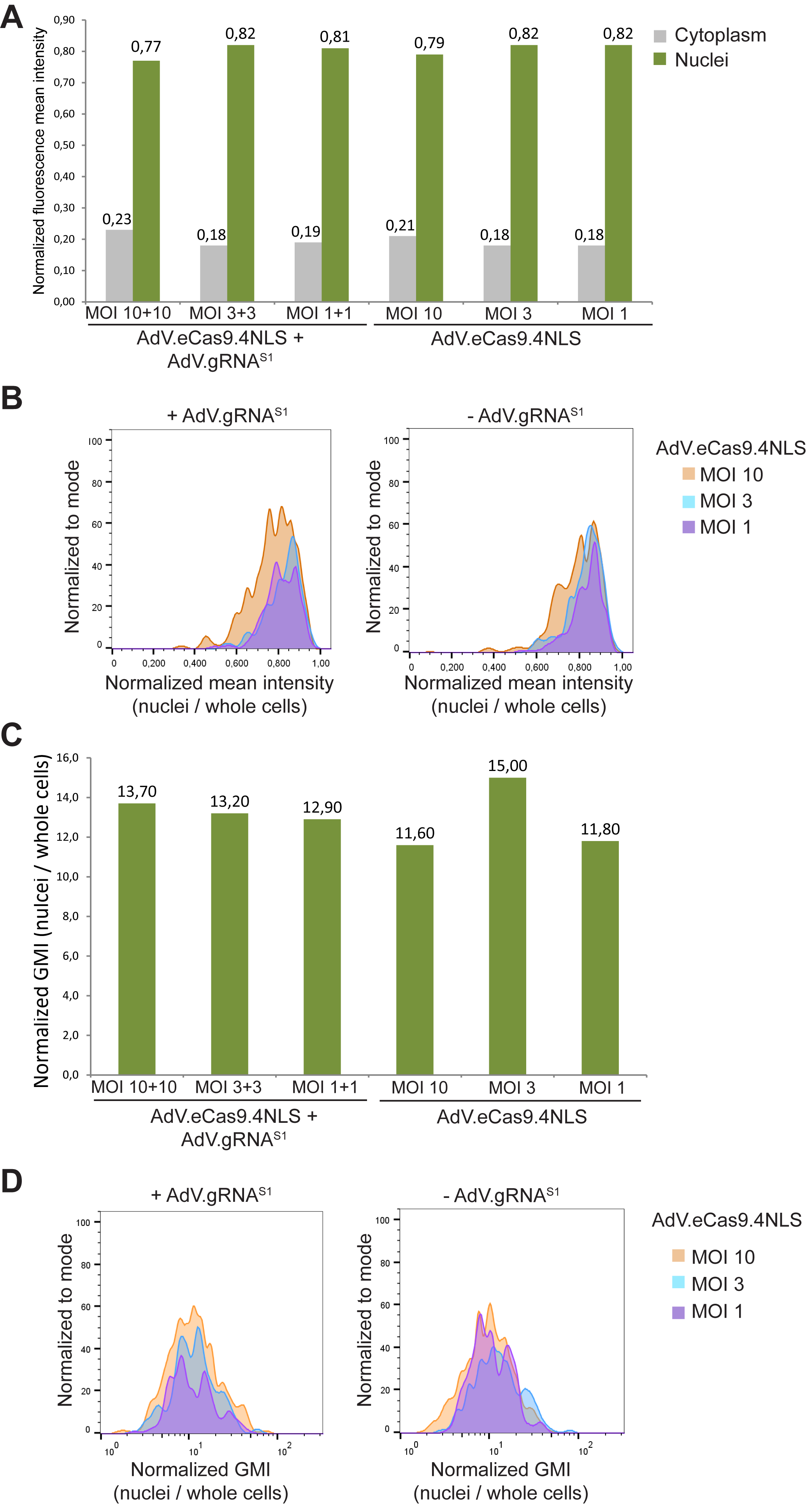


Supplementary Fig. 6 Quantification of the intracellular distribution of eCas9.4NLS in cells transduced with different amounts of AdV particles. (**a**) Fluorescence intensity in HeLa cells stained for eCas9.4NLS. Hela cells were transduced with AdV.eCas9.4NLS mixed with or without AdV.gRNA^S1^ at the indicated multiplicities of infection (MOIs). Values correspond to the mean of SpCas9-specific fluorescence intensities in the nuclear (green bars) and cytoplasmic (grey bars) compartments normalized, on a per-cell-basis, to the whole cellular fluorescence intensity. (**b**) Histograms indicating the distribution of normalized fluorescent intensities of the nuclei in cells staining positive for nuclease expression. (**c**) eCas9.4NLS enrichment in nuclei. Values are calculated by normalizing geometric mean intensity (GMI) in the nuclei versus GMI in the whole cells. (**d**) Histograms showing the distribution of normalized GMI of the nuclei in populations positive for SpCas9 expression. The GMI values from the nuclei were normalized to the GMI values from the whole cells. Data were acquired with Xcyto 10 using a 20× magnification.


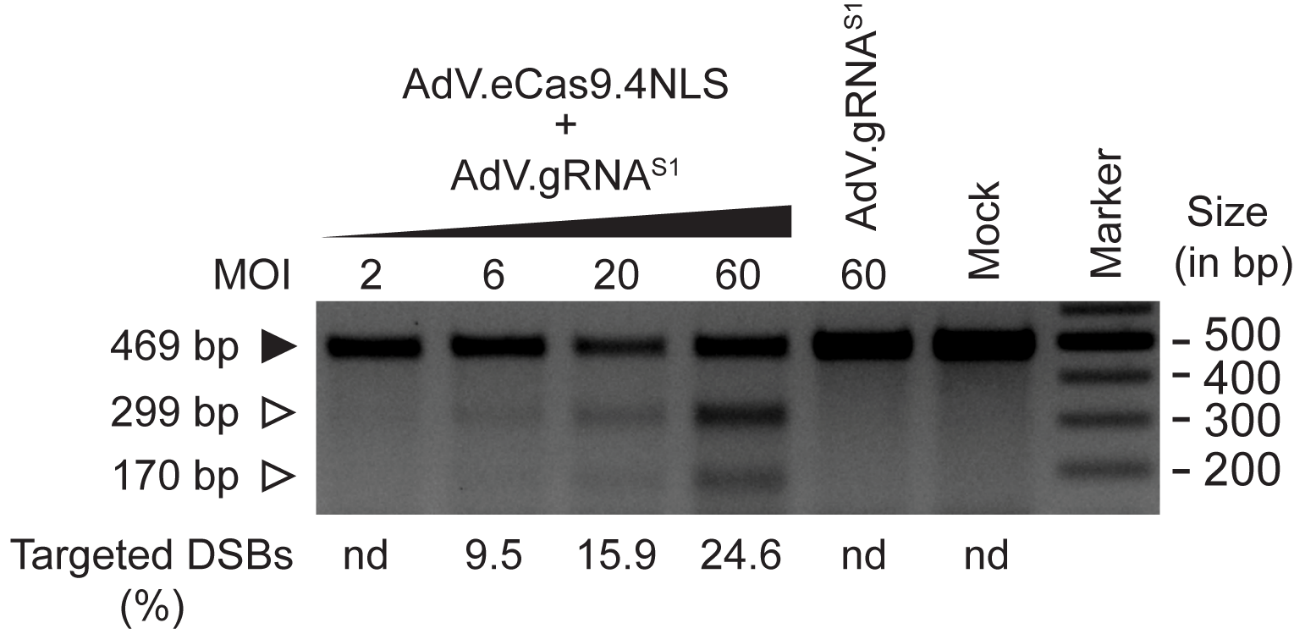


Supplementary Fig. 7 Indel detection at *AAVS1* in cells transduced with AdV.eCas9.4NLS.

Hela cells were transduced with a 1:1 ratio of the indicated MOIs of AdV.eCas9.4NLS and AdV.gRNA^S1^. Negative controls were provided by cells transduced with AdV.gRNA^S1^ alone at an MOI of 60 IU/cell or by mock-transduced cells. Indel frequencies were determined at 3 days post-transduction through T7EI-based genotyping assays. Open and solid arrowheads indicate the positions of DNA species corresponding to digested and undigested amplicons, respectively. Marker, GeneRuler DNA Ladder Mix; nd, not detected.

**
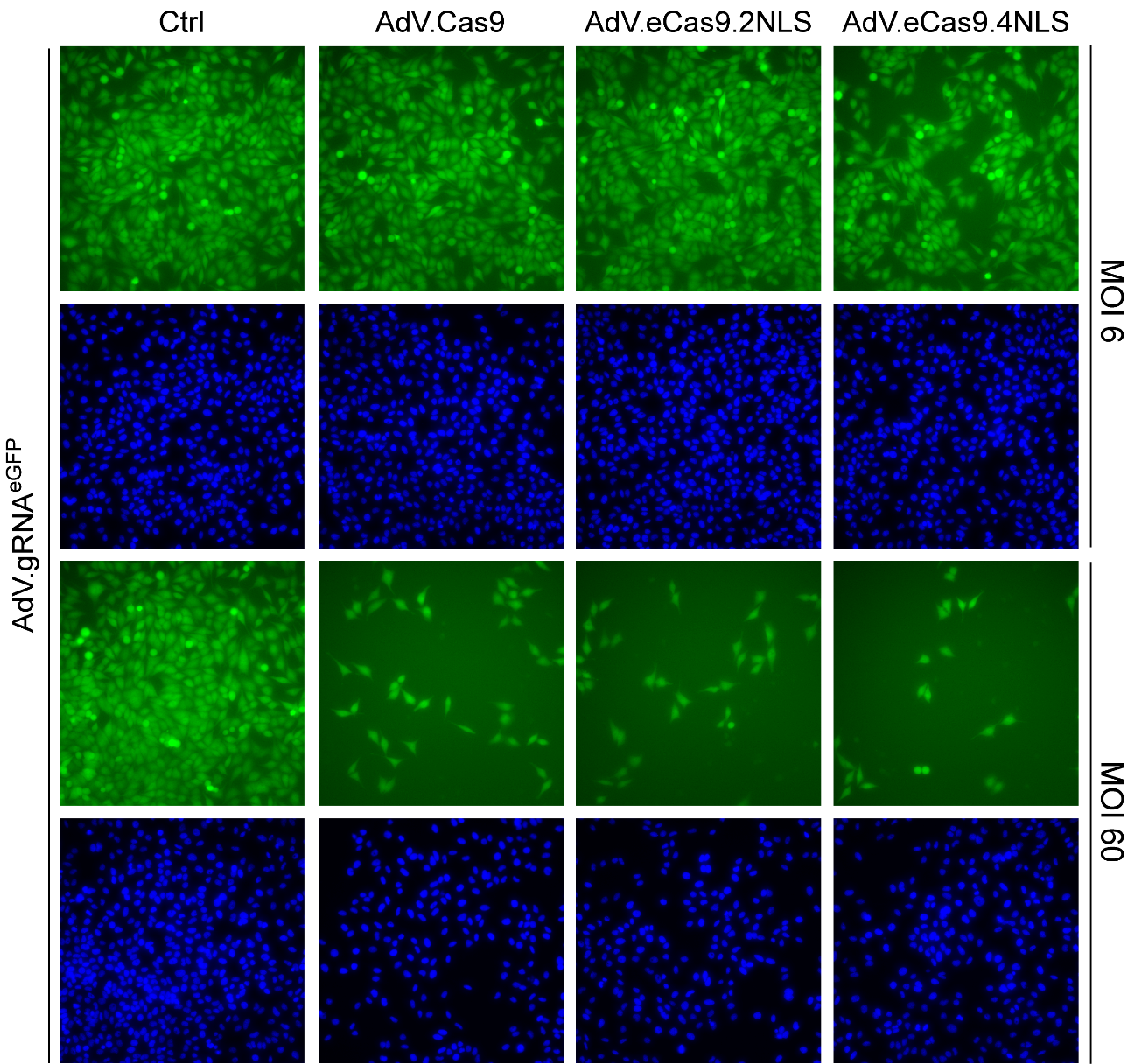
**

Supplementary Fig. 8 Direct fluorescence microscopy on H27 cells transduced with AdVs encoding RGN components**.**

eGFP-positive H27 cells were transduced with the indicated AdVs at a total MOI of 6 or 60 IU/cell. The ratios between SpCas9- and gRNA^eGFP^-encoding AdVs were 1:1. Fluorescence microcopy pictures were acquired at 10 days post-transduction. Negative controls consisted of H27 cells transduced with AdV.gRNA^eGFP^ alone at MOIs of 6 or 60 IU/cell. Cell nuclei and *eGFP*-expressing cells were identified via Hoechst 33342- and eGFP-directed fluorescence microscopy, respectively.

**
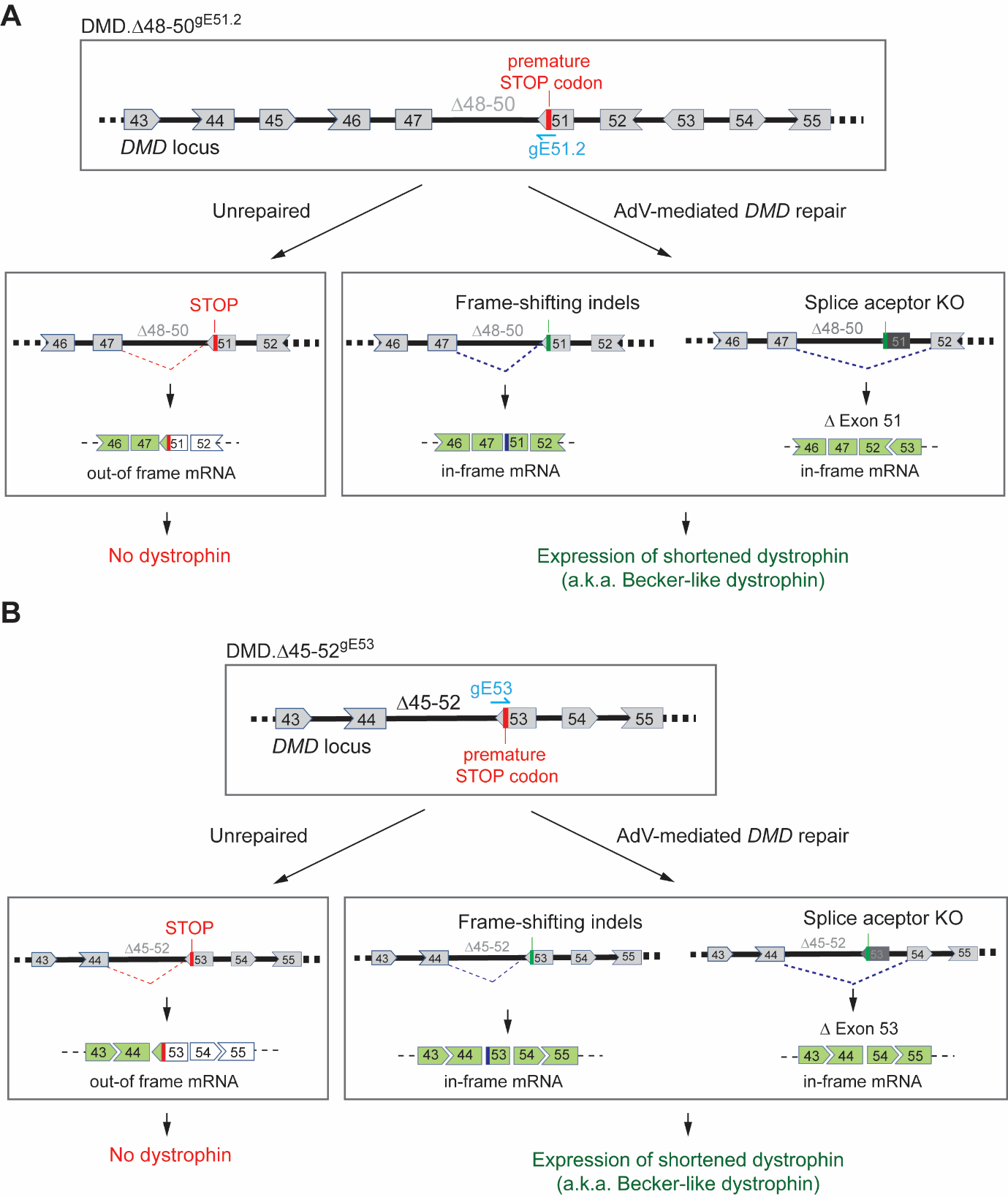
**

**Supplementary Fig. 9** NHEJ-based *DMD* editing strategies for rescuing dystrophin expression in human muscle progenitor cells.

(**a**) *DMD* repair in DMD.Δ48-50^gE51.2^ myoblasts. DMD.Δ48-50^gE51.2^ myoblasts have an intragenic deletion spanning exon 48 through exon 50 of the *DMD* gene. In unedited cells, splicing of exon 47 to exon 51 (dotted red line) leads to out-of-frame mRNA that abolishes dystrophin expression in differentiated muscle cells. Transduction of gE51.2-expressing DMD.Δ48-50^gE51.2^ myoblasts with SpCas9-encoding AdVs yields functional RGNs targeting a sequence located between the splice acceptor of exon 51 and the premature stop codon. NHEJ-derived indels can restore the *DMD* reading frame either via frame-shifting or exon-skipping after splice acceptor knockout. The restored *DMD* reading frame leads to the production of functional, albeit shorter, Becker-like dystrophin proteins. (**b**) *DMD* repair in DMD.Δ45-52^gE53^ myoblasts. DMD.Δ45-52^gE53^ myoblasts have an intragenic deletion spanning exon 45 through exon 52 of the *DMD* gene. In unedited cells, splicing of exon 44 to exon 53 (dotted red line) leads to out-of-frame mRNA that abolishes dystrophin expression in differentiated muscle cells. Transduction of gE53-expressing DMD.Δ45-52^gE53^ myoblasts with SpCas9-encoding AdVs assembles functional RGNs targeting a sequence positioned between the splice acceptor of exon 53 and the premature stop codon. NHEJ-derived indels can restore the *DMD* reading frame either via frame-shifting or exon-skipping after splice acceptor knockout. The repaired *DMD* reading frame induces synthesis of functional, albeit shorter, Becker-like dystrophin proteins.


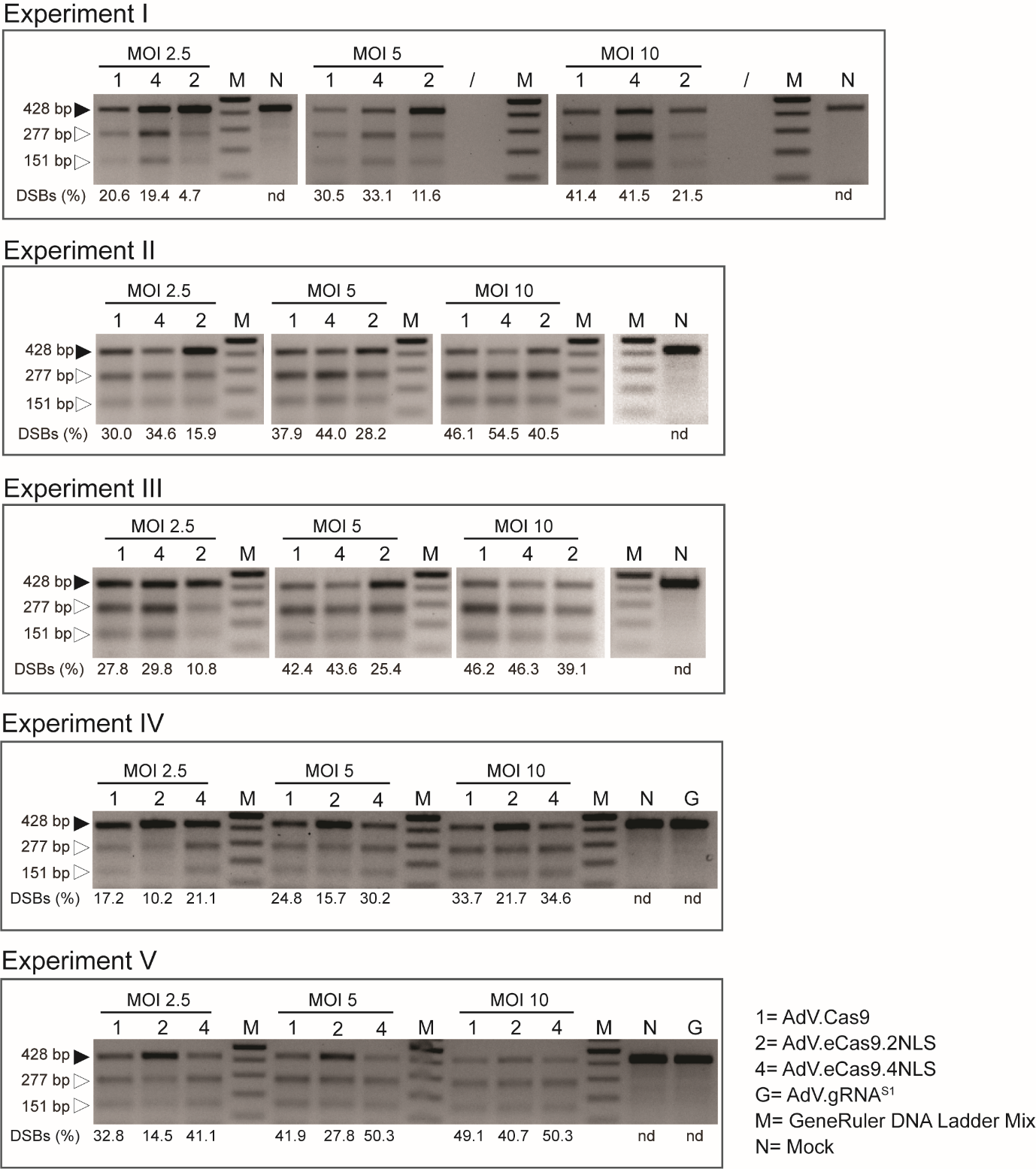


Supplementary Fig. 10 Indel detection at *DMD* in DMD.∆48-50^gE51.2^ myoblasts transduced with AdVs. DMD.∆48-50^gE51.2^ myoblasts were transduced with AdV.Cas9 (1), AdV.eCas9.2NLS (2) or AdV.eCas9.4NLS (4) at the indicated MOIs. Negative controls were provided by DMD.∆48-50^gE51.2^ myoblasts transduced with AdV.gRNA^S1^ alone (G) at an MOI of 10 IU/cell or by mock-transduced cells (N). Indel frequencies were determined at 3 days post-transduction by T7EI-based genotyping assays. Open and solid arrowheads indicate the positions of DNA fragments corresponding to cleaved and non-cleaved amplicons, respectively. The cumulative frequencies of targeted DSBs (indels) are plotted in Fig. 6B. M, GeneRuler DNA Ladder Mix; nd, not detected; MOI, multiplicity of infection; DSBs, targeted double-stranded DNA breaks.

**
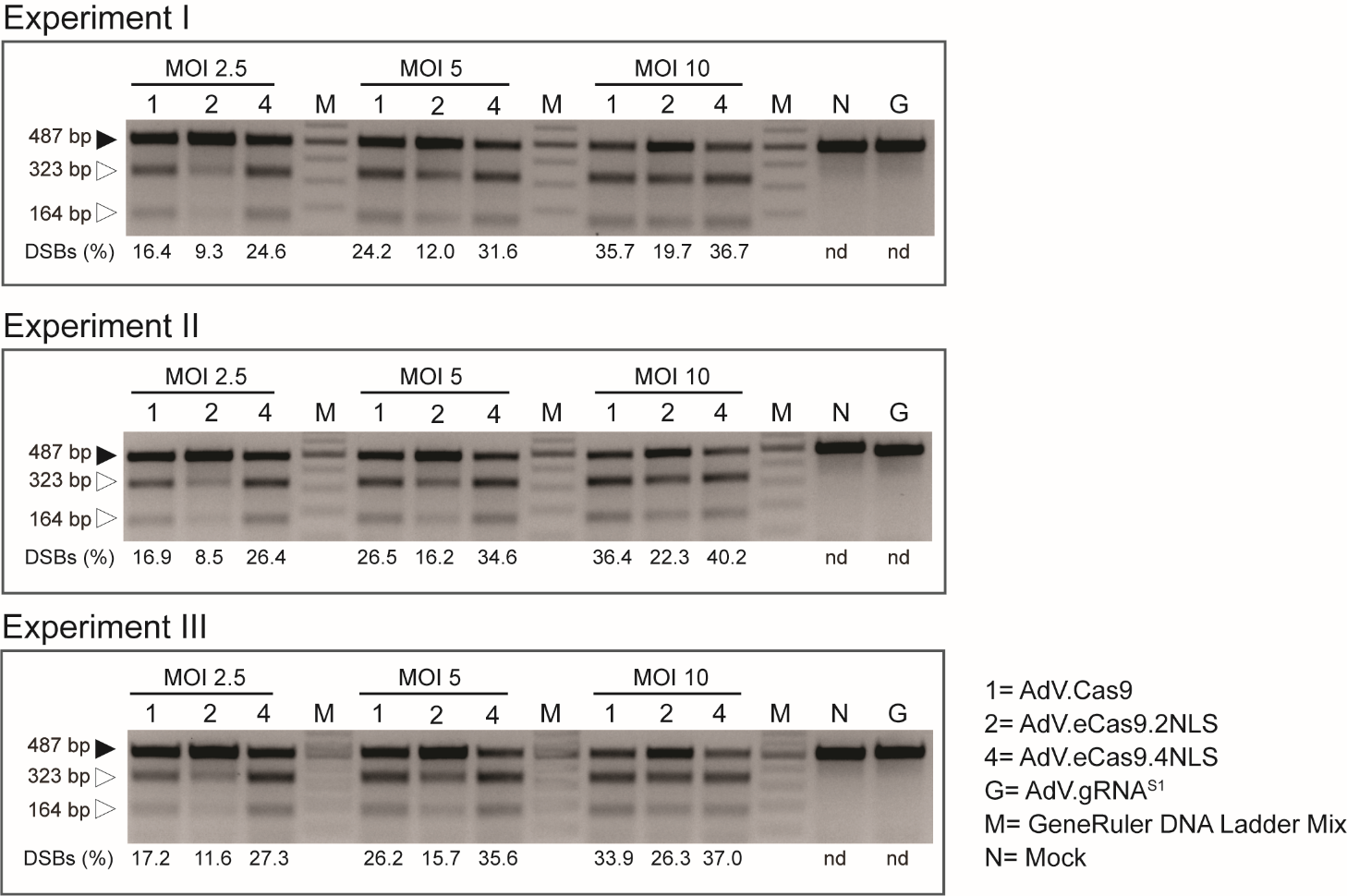
**

Supplementary Fig. 11 Indel detection at *DMD* in DMD.∆45-52^gE53^ myoblasts transduced with AdVs.

DMD.∆45-52^gE53^ myoblasts were transduced with AdV.Cas9 (1), AdV.eCas9.2NLS (2) or AdV.eCas9.4NLS (4) at the indicated MOIs. Negative controls were provided by DMD.∆45-52^gE53^ myoblasts transduced with AdV.gRNA^S1^ alone (G) at an MOI of 10 IU/cell or by mock-transduced cells (N). Indel percentages were determined at 3 days post-transduction by T7EI-based genotyping assays. Open and solid arrowheads indicate the positions of DNA fragments corresponding to cleaved and non-cleaved amplicons, respectively. The cumulative percentages of targeted DSBs (indels) are plotted in Fig. 6C. M, GeneRuler DNA Ladder Mix; nd, not detected; MOI, multiplicity of infection; DSBs, targeted double-stranded DNA breaks.


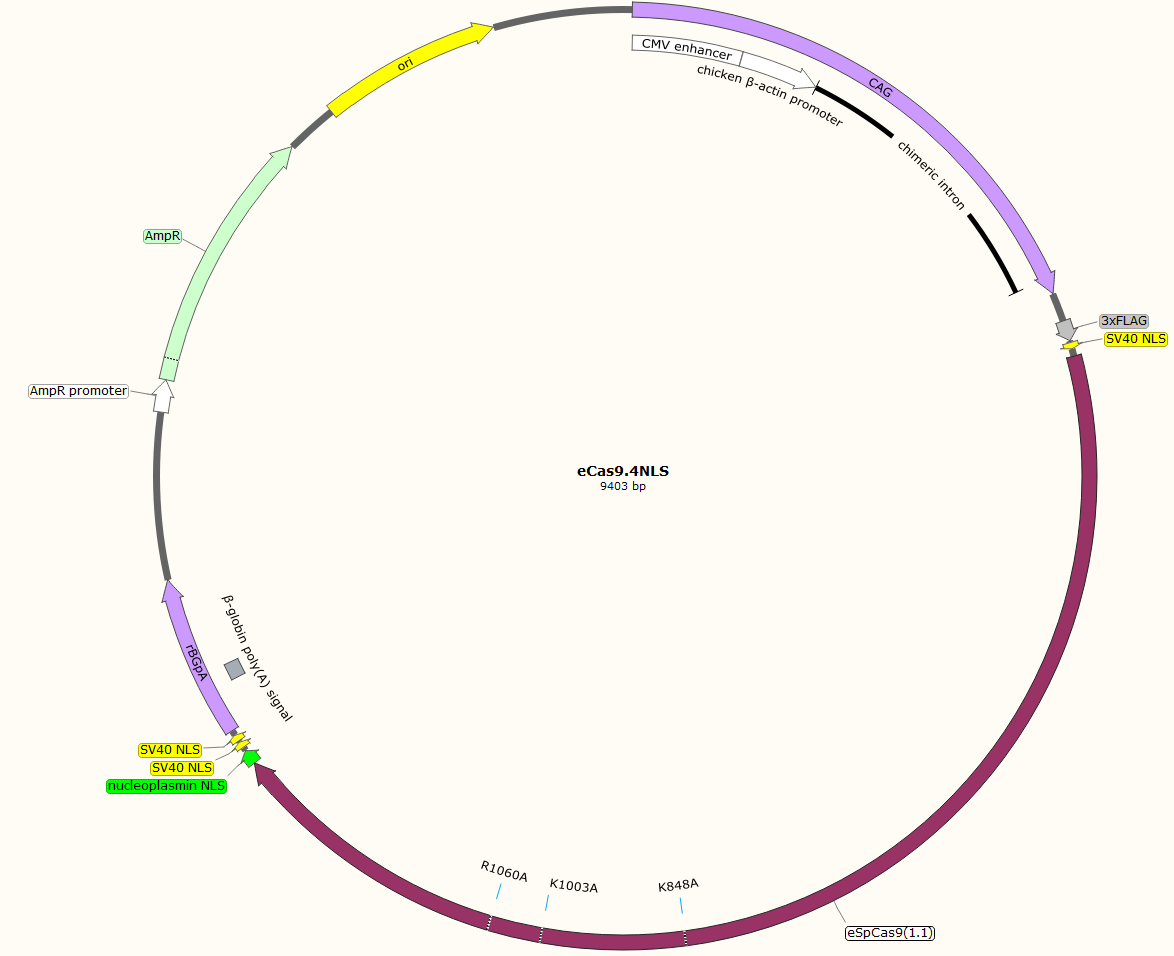


>eCas9.4NLS

GTTTAAACATTTAAATCTCGAGCCATGGATTCGACATTGATTATTGACTAGTTATTAATAGTAATCAATTACGGGGTCATTAGTTCATAGCCCATATATGGAGTTCCGCGTTACATAACTTACGGTAAATGGCCCGCCTGGCTGACCGCCCAACGACCCCCGCCCATTGACGTCAATAATGACGTATGTTCCCATAGTAACGCCAATAGGGACTTTCCATTGACGTCAATGGGTGGAGTATTTACGGTAAACTGCCCACTTGGCAGTACATCAAGTGTATCATATGCCAAGTACGCCCCCTATTGACGTCAATGACGGTAAATGGCCCGCCTGGCATTATGCCCAGTACATGACCTTATGGGACTTTCCTACTTGGCAGTACATCTACGTATTAGTCATCGCTATTACCATGGTCGAGGTGAGCCCCACGTTCTGCTTCACTCTCCCCATCTCCCCCCCCTCCCCACCCCCAATTTTGTATTTATTTATTTTTTAATTATTTTGTGCAGCGATGGGGGCGGGGGGGGGGGGGGCGCGCGCCAGGCGGGGCGGGGCGGGGCGAGGGGCGGGGCGGGGCGAGGCGGAGAGGTGCGGCGGCAGCCAATCAGAGCGGCGCGCTCCGAAAGTTTCCTTTTATGGCGAGGCGGCGGCGGCGGCGGCCCTATAAAAAGCGAAGCGCGCGGCGGGCGGGAGTCGCTGCGTTGCCTTCGCCCCGTGCCCCGCTCCGCGCCGCCTCGCGCCGCCCGCCCCGGCTCTGACTGACCGCGTTACTCCCACAGGTGAGCGGGCGGGACGGCCCTTCTCCTCCGGGCTGTAATTAGCGCTTGGTTTAATGACGGCTCGTTTCTTTTCTGTGGCTGCGTGAAAGCCTTAAAGGGCTCCGGGAGGGCCCTTTGTGCGGGGGGGAGCGGCTCGGGGGGTGCGTGCGTGTGTGTGTGCGTGGGGAGCGCCGCGTGCGGCCCGCGCTGCCCGGCGGCTGTGAGCGCTGCGGGCGCGGCGCGGGGCTTTGTGCGCTCCGCGTGTGCGCGAGGGGAGCGCGGCCGGGGGCGGTGCCCCGCGGTGCGGGGGGGCTGCGAGGGGAACAAAGGCTGCGTGCGGGGTGTGTGCGTGGGGGGGTGAGCAGGGGGTGTGGGCGCGGCGGTCGGGCTGTAACCCCCCCCTGCACCCCCCTCCCCGAGTTGCTGAGCACGGCCCGGCTTCGGGTGCGGGGCTCCGTGCGGGGCGTGGCGCGGGGCTCGCCGTGCCGGGCGGGGGGTGGCGGCAGGTGGGGGTGCCGGGCGGGGCGGGGCCGCCTCGGGCCGGGGAGGGCTCGGGGGAGGGGCGCGGCGGCCCCGGAGCGCCGGCGGCTGTCGAGGCGCGGCGAGCCGCAGCCATTGCCTTTTATGGTAATCGTGCGAGAGGGCGCAGGGACTTCCTTTGTCCCAAATCTGGCGGAGCCGAAATCTGGGAGGCGCCGCCGCACCCCCTCTAGCGGGCGCGGGCGAAGCGGTGCGGCGCCGGCAGGAAGGAAATGGGCGGGGAGGGCCTTCGTGCGTCGCCGCGCCGCCGTCCCCTTCTCCATCTCCAGCCTCGGGGCTGCCGCAGGGGGACGGCTGCCTTCGGGGGGGACGGGGCAGGGCGGGGTTCGGCTTCTGGCGTGTGACCGGCGGCTCTAGAGCCTCTGCTAACCATGTTCATGCCTTCTTCTTTTTCCTACAGCTCCTGGGCAACGTGCTGGTTGTTGTGCTGTCTCATCATTTTGGCAAAGAATTATCGCATGCCTGCAGAGCTCTAGAGTCTAATGTTTAATTACCTGGAGCACCTGCCTGAAATCACTTTTTTTCAGGTTGGACCGGTGCCACCATGGACTATAAGGACCACGACGGAGACTACAAGGATCATGATATTGATTACAAAGACGATGACGATAAGATGGCCCCAAAGAAGAAGCGGAAGGTCGGTATCCACGGAGTCCCAGCAGCCGACAAGAAGTACAGCATCGGCCTGGACATCGGCACCAACTCTGTGGGCTGGGCCGTGATCACCGACGAGTACAAGGTGCCCAGCAAGAAATTCAAGGTGCTGGGCAACACCGACCGGCACAGCATCAAGAAGAACCTGATCGGAGCCCTGCTGTTCGACAGCGGCGAAACAGCCGAGGCCACCCGGCTGAAGAGAACCGCCAGAAGAAGATACACCAGACGGAAGAACCGGATCTGCTATCTGCAAGAGATCTTCAGCAACGAGATGGCCAAGGTGGACGACAGCTTCTTCCACAGACTGGAAGAGTCCTTCCTGGTGGAAGAGGATAAGAAGCACGAGCGGCACCCCATCTTCGGCAACATCGTGGACGAGGTGGCCTACCACGAGAAGTACCCCACCATCTACCACCTGAGAAAGAAACTGGTGGACAGCACCGACAAGGCCGACCTGCGGCTGATCTATCTGGCCCTGGCCCACATGATCAAGTTCCGGGGCCACTTCCTGATCGAGGGCGACCTGAACCCCGACAACAGCGACGTGGACAAGCTGTTCATCCAGCTGGTGCAGACCTACAACCAGCTGTTCGAGGAAAACCCCATCAACGCCAGCGGCGTGGACGCCAAGGCCATCCTGTCTGCCAGACTGAGCAAGAGCAGACGGCTGGAAAATCTGATCGCCCAGCTGCCCGGCGAGAAGAAGAATGGCCTGTTCGGAAACCTGATTGCCCTGAGCCTGGGCCTGACCCCCAACTTCAAGAGCAACTTCGACCTGGCCGAGGATGCCAAACTGCAGCTGAGCAAGGACACCTACGACGACGACCTGGACAACCTGCTGGCCCAGATCGGCGACCAGTACGCCGACCTGTTTCTGGCCGCCAAGAACCTGTCCGACGCCATCCTGCTGAGCGACATCCTGAGAGTGAACACCGAGATCACCAAGGCCCCCCTGAGCGCCTCTATGATCAAGAGATACGACGAGCACCACCAGGACCTGACCCTGCTGAAAGCTCTCGTGCGGCAGCAGCTGCCTGAGAAGTACAAAGAGATTTTCTTCGACCAGAGCAAGAACGGCTACGCCGGCTACATTGACGGCGGAGCCAGCCAGGAAGAGTTCTACAAGTTCATCAAGCCCATCCTGGAAAAGATGGACGGCACCGAGGAACTGCTCGTGAAGCTGAACAGAGAGGACCTGCTGCGGAAGCAGCGGACCTTCGACAACGGCAGCATCCCCCACCAGATCCACCTGGGAGAGCTGCACGCCATTCTGCGGCGGCAGGAAGATTTTTACCCATTCCTGAAGGACAACCGGGAAAAGATCGAGAAGATCCTGACCTTCCGCATCCCCTACTACGTGGGCCCTCTGGCCAGGGGAAACAGCAGATTCGCCTGGATGACCAGAAAGAGCGAGGAAACCATCACCCCCTGGAACTTCGAGGAAGTGGTGGACAAGGGCGCTTCCGCCCAGAGCTTCATCGAGCGGATGACCAACTTCGATAAGAACCTGCCCAACGAGAAGGTGCTGCCCAAGCACAGCCTGCTGTACGAGTACTTCACCGTGTATAACGAGCTGACCAAAGTGAAATACGTGACCGAGGGAATGAGAAAGCCCGCCTTCCTGAGCGGCGAGCAGAAAAAGGCCATCGTGGACCTGCTGTTCAAGACCAACCGGAAAGTGACCGTGAAGCAGCTGAAAGAGGACTACTTCAAGAAAATCGAGTGCTTCGACTCCGTGGAAATCTCCGGCGTGGAAGATCGGTTCAACGCCTCCCTGGGCACATACCACGATCTGCTGAAAATTATCAAGGACAAGGACTTCCTGGACAATGAGGAAAACGAGGACATTCTGGAAGATATCGTGCTGACCCTGACACTGTTTGAGGACAGAGAGATGATCGAGGAACGGCTGAAAACCTATGCCCACCTGTTCGACGACAAAGTGATGAAGCAGCTGAAGCGGCGGAGATACACCGGCTGGGGCAGGCTGAGCCGGAAGCTGATCAACGGCATCCGGGACAAGCAGTCCGGCAAGACAATCCTGGATTTCCTGAAGTCCGACGGCTTCGCCAACAGAAACTTCATGCAGCTGATCCACGACGACAGCCTGACCTTTAAAGAGGACATCCAGAAAGCCCAGGTGTCCGGCCAGGGCGATAGCCTGCACGAGCACATTGCCAATCTGGCCGGCAGCCCCGCCATTAAGAAGGGCATCCTGCAGACAGTGAAGGTGGTGGACGAGCTCGTGAAAGTGATGGGCCGGCACAAGCCCGAGAACATCGTGATCGAAATGGCCAGAGAGAACCAGACCACCCAGAAGGGACAGAAGAACAGCCGCGAGAGAATGAAGCGGATCGAAGAGGGCATCAAAGAGCTGGGCAGCCAGATCCTGAAAGAACACCCCGTGGAAAACACCCAGCTGCAGAACGAGAAGCTGTACCTGTACTACCTGCAGAATGGGCGGGATATGTACGTGGACCAGGAACTGGACATCAACCGGCTGTCCGACTACGATGTGGACCATATCGTGCCTCAGAGCTTTCTGGCCGACGACTCCATCGACAACAAGGTGCTGACCAGAAGCGACAAGAACCGGGGCAAGAGCGACAACGTGCCCTCCGAAGAGGTCGTGAAGAAGATGAAGAACTACTGGCGGCAGCTGCTGAACGCCAAGCTGATTACCCAGAGAAAGTTCGACAATCTGACCAAGGCCGAGAGAGGCGGCCTGAGCGAACTGGATAAGGCCGGCTTCATCAAGAGACAGCTGGTGGAAACCCGGCAGATCACAAAGCACGTGGCACAGATCCTGGACTCCCGGATGAACACTAAGTACGACGAGAATGACAAGCTGATCCGGGAAGTGAAAGTGATCACCCTGAAGTCCAAGCTGGTGTCCGATTTCCGGAAGGATTTCCAGTTTTACAAAGTGCGCGAGATCAACAACTACCACCACGCCCACGACGCCTACCTGAACGCCGTCGTGGGAACCGCCCTGATCAAAAAGTACCCTGCGCTGGAAAGCGAGTTCGTGTACGGCGACTACAAGGTGTACGACGTGCGGAAGATGATCGCCAAGAGCGAGCAGGAAATCGGCAAGGCTACCGCCAAGTACTTCTTCTACAGCAACATCATGAACTTTTTCAAGACCGAGATTACCCTGGCCAACGGCGAGATCCGGAAGGCGCCTCTGATCGAGACAAACGGCGAAACCGGGGAGATCGTGTGGGATAAGGGCCGGGATTTTGCCACCGTGCGGAAAGTGCTGAGCATGCCCCAAGTGAATATCGTGAAAAAGACCGAGGTGCAGACAGGCGGCTTCAGCAAAGAGTCTATCCTGCCCAAGAGGAACAGCGATAAGCTGATCGCCAGAAAGAAGGACTGGGACCCTAAGAAGTACGGCGGCTTCGACAGCCCCACCGTGGCCTATTCTGTGCTGGTGGTGGCCAAAGTGGAAAAGGGCAAGTCCAAGAAACTGAAGAGTGTGAAAGAGCTGCTGGGGATCACCATCATGGAAAGAAGCAGCTTCGAGAAGAATCCCATCGACTTTCTGGAAGCCAAGGGCTACAAAGAAGTGAAAAAGGACCTGATCATCAAGCTGCCTAAGTACTCCCTGTTCGAGCTGGAAAACGGCCGGAAGAGAATGCTGGCCTCTGCCGGCGAACTGCAGAAGGGAAACGAACTGGCCCTGCCCTCCAAATATGTGAACTTCCTGTACCTGGCCAGCCACTATGAGAAGCTGAAGGGCTCCCCCGAGGATAATGAGCAGAAACAGCTGTTTGTGGAACAGCACAAGCACTACCTGGACGAGATCATCGAGCAGATCAGCGAGTTCTCCAAGAGAGTGATCCTGGCCGACGCTAATCTGGACAAAGTGCTGTCCGCCTACAACAAGCACCGGGATAAGCCCATCAGAGAGCAGGCCGAGAATATCATCCACCTGTTTACCCTGACCAATCTGGGAGCCCCTGCCGCCTTCAAGTACTTTGACACCACCATCGACCGGAAGAGGTACACCAGCACCAAAGAGGTGCTGGACGCCACCCTGATCCACCAGAGCATCACCGGCCTGTACGAGACACGGATCGACCTGTCTCAGCTGGGAGGCGACAAAAGGCCGGCGGCCACGAAAAAGGCCGGCCAGGCAAAAAAGAAAAAGGCTAGCGGCTCCCCCAAGAAAAAACGCAAGGTGGAAGATCCTAAGAAAAAGCGGAAAGTGTAAGAATTCGCGTGGAAATTCACTCCTCAGGTGCAGGCTGCCTATCAGAAGGTGGTGGCTGGTGTGGCCAATGCCCTGGCTCACAAATACCACTGAGATCTTTTTCCCTCTGCCAAAAATTATGGGGACATCATGAAGCCCCTTGAGCATCTGACTTCTGGCTAATAAAGGAAATTTATTTTCATTGCAATAGTGTGTTGGAATTTTTTGTGTCTCTCACTCGGAAGGACATATGGGAGGGCAAATCATTTAAAACATCAGAATGAGTATTTGGTTTAGAGTTTGGCAACATATGCCCATATGCTGGCTGCCATGAACAAAGGTTGGCTATAAAGAGGTCATCAGTATATGAAACAGCCCCCTGCTGTCCATTCCTTATTCCATAGAAAAGCCTTGACTTGAGGTTAGATTTTTTTTATATTTTGTTTTGTGTTATTTTTTTCTTTAACATCCCTAAAATTTTCCTTACATGTTTTACTAGCCAGATTTTTCCTCCTCTCCTGACTACTCCCAGTCATAGCTGTCCCTCTTCTCTTATGGAGATCCCTCGACCTGCAGCCCAAGCTGATCCCGGGATTTAAATGTTTAAACGAATTCACTGGCCGTCGTTTTACAACGTCGTGACTGGGAAAACCCTGGCGTTACCCAACTTAATCGCCTTGCAGCACATCCCCCTTTCGCCAGCTGGCGTAATAGCGAAGAGGCCCGCACCGATCGCCCTTCCCAACAGTTGCGCAGCCTGAATGGCGAATGGCGCCTGATGCGGTATTTTCTCCTTACGCATCTGTGCGGTATTTCACACCGCATATGGTGCACTCTCAGTACAATCTGCTCTGATGCCGCATAGTTAAGCCAGCCCCGACACCCGCCAACACCCGCTGACGCGCCCTGACGGGCTTGTCTGCTCCCGGCATCCGCTTACAGACAAGCTGTGACCGTCTCCGGGAGCTGCATGTGTCAGAGGTTTTCACCGTCATCACCGAAACGCGCGAGACGAAAGGGCCTCGTGATACGCCTATTTTTATAGGTTAATGTCATGATAATAATGGTTTCTTAGACGTCAGGTGGCACTTTTCGGGGAAATGTGCGCGGAACCCCTATTTGTTTATTTTTCTAAATACATTCAAATATGTATCCGCTCATGAGACAATAACCCTGATAAATGCTTCAATAATATTGAAAAAGGAAGAGTATGAGTATTCAACATTTCCGTGTCGCCCTTATTCCCTTTTTTGCGGCATTTTGCCTTCCTGTTTTTGCTCACCCAGAAACGCTGGTGAAAGTAAAAGATGCTGAAGATCAGTTGGGTGCACGAGTGGGTTACATCGAACTGGATCTCAACAGCGGTAAGATCCTTGAGAGTTTTCGCCCCGAAGAACGTTTTCCAATGATGAGCACTTTTAAAGTTCTGCTATGTGGCGCGGTATTATCCCGTATTGACGCCGGGCAAGAGCAACTCGGTCGCCGCATACACTATTCTCAGAATGACTTGGTTGAGTACTCACCAGTCACAGAAAAGCATCTTACGGATGGCATGACAGTAAGAGAATTATGCAGTGCTGCCATAACCATGAGTGATAACACTGCGGCCAACTTACTTCTGACAACGATCGGAGGACCGAAGGAGCTAACCGCTTTTTTGCACAACATGGGGGATCATGTAACTCGCCTTGATCGTTGGGAACCGGAGCTGAATGAAGCCATACCAAACGACGAGCGTGACACCACGATGCCTGTAGCAATGGCAACAACGTTGCGCAAACTATTAACTGGCGAACTACTTACTCTAGCTTCCCGGCAACAATTAATAGACTGGATGGAGGCGGATAAAGTTGCAGGACCACTTCTGCGCTCGGCCCTTCCGGCTGGCTGGTTTATTGCTGATAAATCTGGAGCCCGTGAGCGTGGGTCTCGCGGTATCATTGCAGCACTGGGGCCAGATGGTAAGCCCTCCCGTATCGTAGTTATCTACACGACGGGGAGTCAGGCAACTATGGATGAACGAAATAGACAGATCGCTGAGATAGGTGCCTCACTGATTAAGCATTGGTAACTGTCAGACCAAGTTTACTCATATATACTTTAGATTGATTTAAAACTTCATTTTTAATTTAAAAGGATCTAGGTGAAGATCCTTTTTGATAATCTCATGACCAAAATCCCTTAACGTGAGTTTTCGTTCCACTGAGCGTCAGACCCCGTAGAAAAGATCAAAGGATCTTCTTGAGATCCTTTTTTTCTGCGCGTAATCTGCTGCTTGCAAACAAAAAAACCACCGCTACCAGCGGTGGTTTGTTTGCCGGATCAAGAGCTACCAACTCTTTTTCCGAAGGTAACTGGCTTCAGCAGAGCGCAGATACCAAATACTGTCCTTCTAGTGTAGCCGTAGTTAGGCCACCACTTCAAGAACTCTGTAGCACCGCCTACATACCTCGCTCTGCTAATCCTGTTACCAGTGGCTGCTGCCAGTGGCGATAAGTCGTGTCTTACCGGGTTGGACTCAAGACGATAGTTACCGGATAAGGCGCAGCGGTCGGGCTGAACGGGGGGTTCGTGCACACAGCCCAGCTTGGAGCGAACGACCTACACCGAACTGAGATACCTACAGCGTGAGCTATGAGAAAGCGCCACGCTTCCCGAAGGGAGAAAGGCGGACAGGTATCCGGTAAGCGGCAGGGTCGGAACAGGAGAGCGCACGAGGGAGCTTCCAGGGGGAAACGCCTGGTATCTTTATAGTCCTGTCGGGTTTCGCCACCTCTGACTTGAGCGTCGATTTTTGTGATGCTCGTCAGGGGGGCGGAGCCTATGGAAAAACGCCAGCAACGCGGCCTTTTTACGGTTCCTGGCCTTTTGCTGGCCTTTTGCTCACATGTTCTTTCCTGCGTTATCCCCTGATTCTGTGGATAACCGTATTACCGCCTTTGAGTGAGCTGATACCGCTCGCCGCAGCCGAACGACCGAGCGCAGCGAGTCAGTGAGCGAGGAAGCGGAAGAGCGCCCAATACGCAAACCGCCTCTCCCCGCGCGTTGGCCGATTCATTAATGCAGCTGGCACGACAGGTTTCCCGACTGGAAAGCGGGCAGTGAGCGCAACGCAATTAATGTGAGTTAGCTCACTCATTAGGCACCCCAGGCTTTACACTTTATGCTTCCGGCTCGTATGTTGTGTGGAATTGTGAGCGGATAACAATTTCACACAGGAAACAGCTATGACCATGATTACGCCAAGCTT

Supplementary Fig. 12 Map and nucleotide sequence of the eCas9.4NLS plasmid.

CAG, CMV *immediate-early* enhancer/chicken *β-actin* promoter; 3× FLAG, tandem of three FLAG epitope tags; SV40 NLS, nuclear localization signal from the SV40 large T antigen; eSpCas9(1.1), Cas9 endonuclease from the type II CRISPR/Cas system from *Streptococcus pyogenes* with the mutations conferring enhanced specificity annotated; nucleoplasmin NLS, nuclear localization signal derived from the nucleoplasmin of *Xenopus sp.*; AmpR, *β-lactamase* gene conferring resistance to ampicillin; ori, prokaryotic origin of replication. The different features of the eCas9.4NLS ORF are color coded in the plasmid map and respective nucleotide sequence. The yellow codons specify adenine mutations at positions 848, 1003 and 1060 of the *S. pyogenes* Cas9 ORF conferring high-specificity to the nuclease.


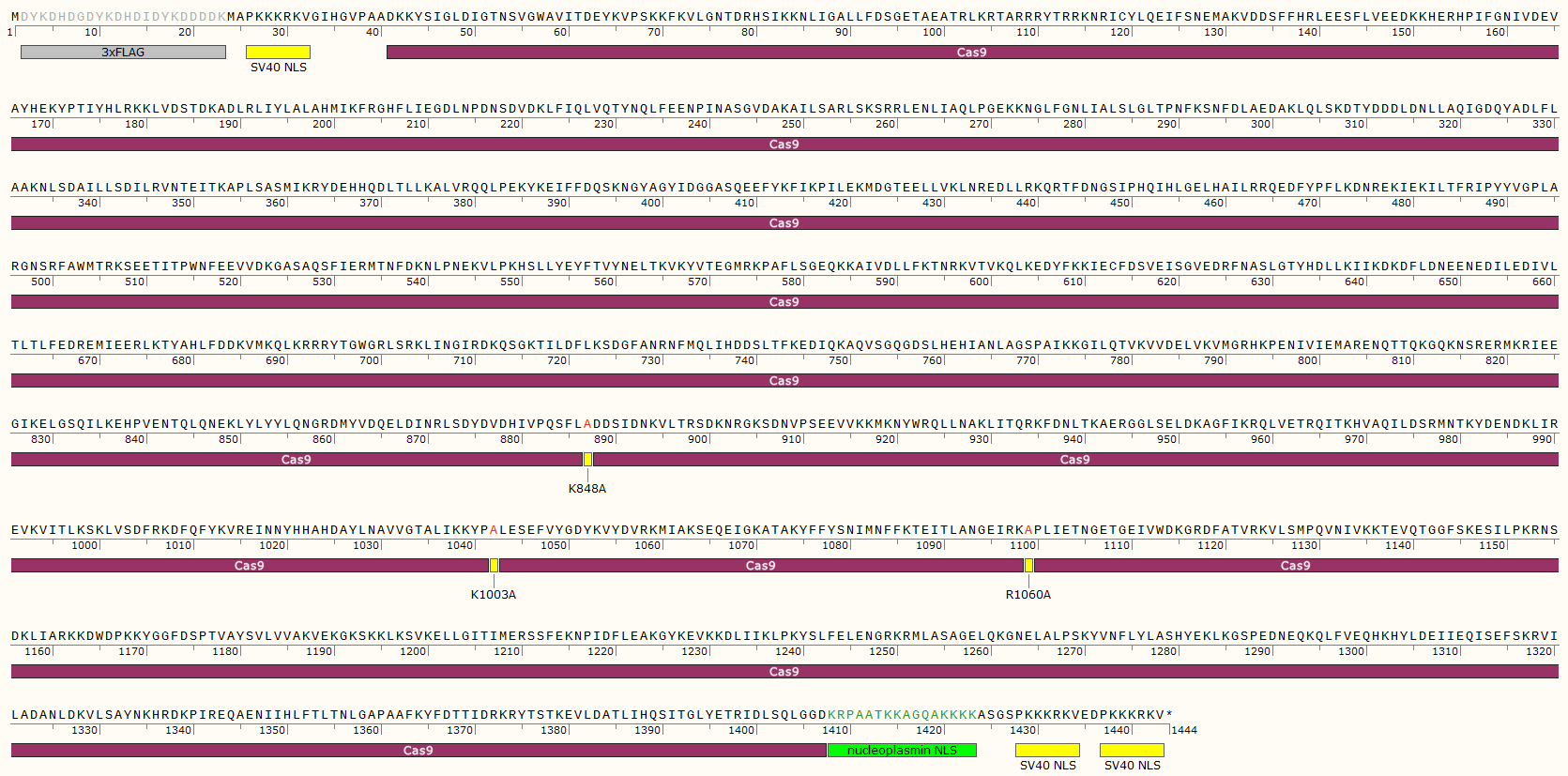


Supplementary Fig. 13 Amino acid sequence of the eCas9.4NLS nuclease.

3×FLAG, tandem of three FLAG epitope tags; SV40 NLS, nuclear localization signal from the SV40 large T antigen; nucleoplasmin NLS, nuclear localization signal from *Xenopus sp.* nucleoplasmin. The mutations K848A, K1003A and R1060A conferring enhanced specificity to eCas9.2NLS and its derivative eCas9.4NLS are indicated. These mutation positions correspond to the *S. pyogenes* Cas9 protein amino acid sequence.


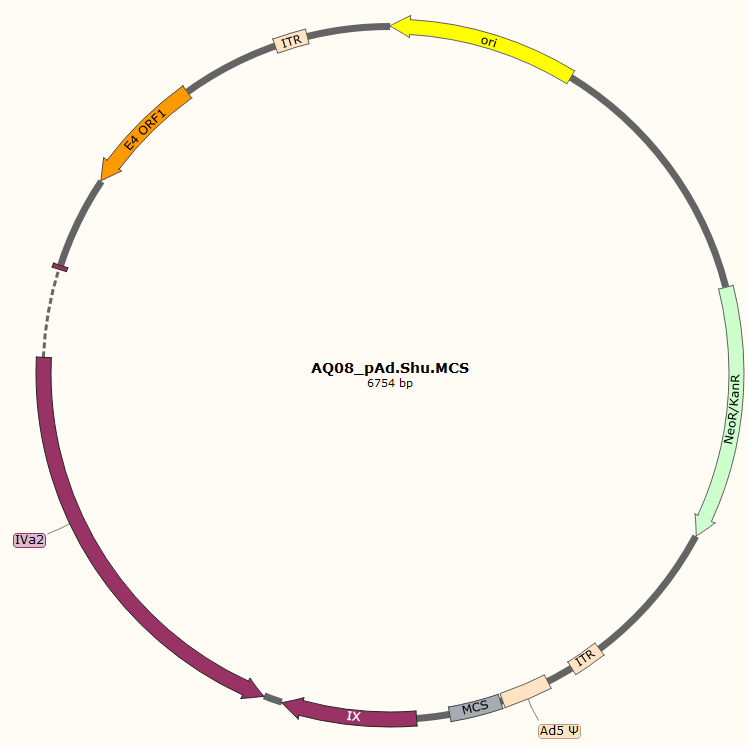


>AQ08_pAd.Shu.MCS

TTTCCATAGGCTCCGCCCCCCTGACGAGCATCACAAAAATCGACGCTCAAGTCAGAGGTGGCGAAACCCGACAGGACTATAAAGATACCAGGCGTTTCCCCCTGGAAGCTCCCTCGTGCGCTCTCCTGTTCCGACCCTGCCGCTTACCGGATACCTGTCCGCCTTTCTCCCTTCGGGAAGCGTGGCGCTTTCTCATAGCTCACGCTGTAGGTATCTCAGTTCGGTGTAGGTCGTTCGCTCCAAGCTGGGCTGTGTGCACGAACCCCCCGTTCAGCCCGACCGCTGCGCCTTATCCGGTAACTATCGTCTTGAGTCCAACCCGGTAAGACACGACTTATCGCCACTGGCAGCAGCCACTGGTAACAGGATTAGCAGAGCGAGGTATGTAGGCGGTGCTACAGAGTTCTTGAAGTGGTGGCCTAACTACGGCTACACTAGAAGGACAGTATTTGGTATCTGCGCTCTGCTGAAGCCAGTTACCTTCGGAAAAAGAGTTGGTAGCTCTTGATCCGGCAAACAAACCACCGCTGGTAGCGGTGGTTTTTTTGTTTGCAAGCAGCAGATTACGCGCAGAAAAAAAGGATCTCAAGAAGATCCTTTGATCTTTTCTACGGGGTCTGACGCTCAGTGGAACGAAAACTCACGTTAAGGGATTTTGGTCATGAGATTATCAAAAAGGATCTTCACCTAGATCCTTTTAAATTAAAAATGAAGTTTTAAATCAATCTAAAGTATATATGAGTAAACTTGGTCTGACAGTTACCAATGCTTAATCAGTGAGGCACCTATCTCAGCGATCTGTCTATTTCGTTCATCCATAGTTGCCTGACTCCCCGTCGTGTAGATAACTACGATACGGGAGGGCTTACCATCTGGCCCCAGTGCTGCAATGATACCGCGAGACCCACGCTCACCGGCTCCAGATTTATCAGCAATAAACCAGCCAGCCGGAAGGGCCGAGCGCAGAAGTGGTCCTGCAACTTTATCCGCCTCCATCCAGTCTATTAATTGTTGCCGGGAAGCTAGAGTAAGTAGTTCGCCAGTTAATAGTTTGCGCAACGTTGTTGCCATTGCTGCAGCCATGAGATTATCAAAAAGGATCTTCACCTAGATCCTTTTCACGTAGAAAGCCAGTCCGCAGAAACGGTGCTGACCCCGGATGAATGTCAGCTACTGGGCTATCTGGACAAGGGAAAACGCAAGCGCAAAGAGAAAGCAGGTAGCTTGCAGTGGGCTTACATGGCGATAGCTAGACTGGGCGGTTTTATGGACAGCAAGCGAACCGGAATTGCCAGCTGGGGCGCCCTCTGGTAAGGTTGGGAAGCCCTGCAAAGTAAACTGGATGGCTTTCTTGCCGCCAAGGATCTGATGGCGCAGGGGATCAAGCTCTGATCAAGAGACAGGATGAGGATCGTTTCGCATGATTGAACAAGATGGATTGCACGCAGGTTCTCCGGCCGCTTGGGTGGAGAGGCTATTCGGCTATGACTGGGCACAACAGACAATCGGCTGCTCTGATGCCGCCGTGTTCCGGCTGTCAGCGCAGGGGCGCCCGGTTCTTTTTGTCAAGACCGACCTGTCCGGTGCCCTGAATGAACTGCAAGACGAGGCAGCGCGGCTATCGTGGCTGGCCACGACGGGCGTTCCTTGCGCAGCTGTGCTCGACGTTGTCACTGAAGCGGGAAGGGACTGGCTGCTATTGGGCGAAGTGCCGGGGCAGGATCTCCTGTCATCTCACCTTGCTCCTGCCGAGAAAGTATCCATCATGGCTGATGCAATGCGGCGGCTGCATACGCTTGATCCGGCTACCTGCCCATTCGACCACCAAGCGAAACATCGCATCGAGCGAGCACGTACTCGGATGGAAGCCGGTCTTGTCGATCAGGATGATCTGGACGAAGAGCATCAGGGGCTCGCGCCAGCCGAACTGTTCGCCAGGCTCAAGGCGAGCATGCCCGACGGCGAGGATCTCGTCGTGACCCATGGCGATGCCTGCTTGCCGAATATCATGGTGGAAAATGGCCGCTTTTCTGGATTCATCGACTGTGGCCGGCTGGGTGTGGCGGACCGCTATCAGGACATAGCGTTGGCTACCCGTGATATTGCTGAAGAGCTTGGCGGCGAATGGGCTGACCGCTTCCTCGTGCTTTACGGTATCGCCGCTCCCGATTCGCAGCGCATCGCCTTCTATCGCCTTCTTGACGAGTTCTTCTGAATTTTGTTAAAATTTTTGTTAAATCAGCTCATTTTTTAACCAATAGGCCGAAATCGGCAAAATCCCTTATAAATCAAAAGAATAGACCGAGATAGGGTTGAGTGTTGTTCCAGTTTGGAACAAGAGTCCACTATTAAAGAACGTGGACTCCAACGTCAAAGGGCGAAAAACCGTCTATCAGGGCGATGGCCCACTACGTGAACCATCACCCTAATCAAGTTTTTTGGGGTCGAGGTGCCGTAAAGCACTAAATCGGAACCCTAAAGGGAGCCCCCGATTTAGAGCTTGACGGGGAAAGCCGGCGAACGTGGCGAGAAAGGAAGGGAAGAAAGCGAAAGGAGCGGGCGCTAGGGCGCTGGCAAGTGTAGCGGTCACGCTGCGCGTAACCACCACACCCGCCGCGCTTAATGCGCCGCTACAGGGCGCGTCCATTCGCCATTCAGGATCGAATTAATTCTTAATTAACATCATCAATAATATACCTTATTTTGGATTGAAGCCAATATGATAATGAGGGGGTGGAGTTTGTGACGTGGCGCGGGGCGTGGGAACGGGGCGGGTGACGTAGTAGTGTGGCGGAAGTGTGATGTTGCAAGTGTGGCGGAACACATGTAAGCGACGGATGTGGCAAAAGTGACGTTTTTGGTGTGCGCCGGTGTACACAGGAAGTGACAATTTTCGCGCGGTTTTAGGCGGATGTTGTAGTAAATTTGGGCGTAACCGAGTAAGATTTGGCCATTTTCGCGGGAAAACTGAATAAGAGGAAGTGAAATCTGAATAATTTTGTGTTACTCATAGCGCGTAATACTGGTACCGGTTGTTAACGTTAGCCGGCTACGTATACTCCGGAATATTAATAGGCCTAGGATGCATATGGCGGCCGCCTGCAGCTGGCGCCATCGATACGCGTACGTCGCGACCGCGGACATGTACAGAGCTCGAGAAGTACTAGTGGCCACGTGGGCCGTGCACCTTAAGCTTGTCGACTCGAAGATCTGGGCGTGGTTAAGGGTGGGAAAGAATATATAAGGTGGGGGTCTTATGTAGTTTTGTATCTGTTTTGCAGCAGCCGCCGCCGCCATGAGCACCAACTCGTTTGATGGAAGCATTGTGAGCTCATATTTGACAACGCGCATGCCCCCATGGGCCGGGGTGCGTCAGAATGTGATGGGCTCCAGCATTGATGGTCGCCCCGTCCTGCCCGCAAACTCTACTACCTTGACCTACGAGACCGTGTCTGGAACGCCGTTGGAGACTGCAGCCTCCGCCGCCGCTTCAGCCGCTGCAGCCACCGCCCGCGGGATTGTGACTGACTTTGCTTTCCTGAGCCCGCTTGCAAGCAGTGCAGCTTCCCGTTCATCCGCCCGCGATGACAAGTTGACGGCTCTTTTGGCACAATTGGATTCTTTGACCCGGGAACTTAATGTCGTTTCTCAGCAGCTGTTGGATCTGCGCCAGCAGGTTTCTGCCCTGAAGGCTTCCTCCCCTCCCAATGCGGTTTAAAACATAAATAAAAAACCAGACTCTGTTTGGATTTGGATCAAGCAAGTGTCTTGCTGTCTTTATTTAGGGGTTTTGCGCGCGCGGTAGGCCCGGGACCAGCGGTCTCGGTCGTTGAGGGTCCTGTGTATTTTTTCCAGGACGTGGTAAAGGTGACTCTGGATGTTCAGATACATGGGCATAAGCCCGTCTCTGGGGTGGAGGTAGCACCACTGCAGAGCTTCATGCTGCGGGGTGGTGTTGTAGATGATCCAGTCGTAGCAGGAGCGCTGGGCGTGGTGCCTAAAAATGTCTTTCAGTAGCAAGCTGATTGCCAGGGGCAGGCCCTTGGTGTAAGTGTTTACAAAGCGGTTAAGCTGGGATGGGTGCATACGTGGGGATATGAGATGCATCTTGGACTGTATTTTTAGGTTGGCTATGTTCCCAGCCATATCCCTCCGGGGATTCATGTTGTGCAGAACCACCAGCACAGTGTATCCGGTGCACTTGGGAAATTTGTCATGTAGCTTAGAAGGAAATGCGTGGAAGAACTTGGAGACGCCCTTGTGACCTCCAAGATTTTCCATGCATTCGTCCATAATGATGGCAATGGGCCCACGGGCGGCGGCCTGGGCGAAGATATTTCTGGGATCACTAACGTCATAGTTGTGTTCCAGGATGAGATCGTCATAGGCCATTTTTACAAAGCGCGGGCGGAGGGTGCCAGACTGCGGTATAATGGTTCCATCCGGCCCAGGGGCGTAGTTACCCTCACAGATTTGCATTTCCCACGCTTTGAGTTCAGATGGGGGGATCATGTCTACCTGCGGGGCGATGAAGAAAACGGTTTCCGGGGTAGGGGAGATCAGCTGGGAAGAAAGCAGGTTCCTGAGCAGCTGCGACTTACCGCAGCCGGTGGGCCCGTAAATCACACCTATTACCGGGTGCAACTGGTAGTTAAGAGAGCTGCAGCTGCCGTCATCCCTGAGCAGGGGGGCCACTTCGTTAAGCATGTCCCTGACTCGCATGTTTTCCCTGACCAAATCCGCCAGAAGGCGCTCGCCGCCCAGCGATAGCAGTTCTTGCAAGGAAGCAAAGTTTTTCAACGGTTTGAGACCGTCCGCCGTAGGCATGCTTTTGAGCGTTTGACCAAGCAGTTCCAGGCGGTCCCACAGCTCGGTCACCTGCTCTACGGCATCTCGATCCAGCATATCTCCTCGTTTCGCGGGTTGGGGCGGCTTTCGCTGTACGGCAGTAGTCGGTGCTCGTCCAGACGGGCCAGGGTCATGTCTTTCCACGGGCGCAGGGTCCTCGTCAGCGTAGTCTGGGTCACGGTGAAGGGGTGCGCTCCGGGCTGCGCGCTGGCCAGGGTGCGCTTGAGGCTGGTCCTGCTGGTGCTGAAGCGCTGCCGGTCTTCGCCCTGCGCGTCGGCCAGGTAGCATTTGACCATGGTGTCATAGTCCAGCCCCTCCGCGGCGTGGCCCTTGGCGCGCAGCTTGCCCTTGGAGGAGGCGCCGCACGAGGGGCAGTGCAGACTTTTGAGGGCGTAGAGCTTGGGCGCGAGAAATACCGATTCCGGGGAGTAGGCATCCGCGCCGCAGGCCCCGCAGACGGTCTCGCATTCCACGAGCCAGGTGAGCTCTGGCCGTTCGGGGTCAAAAACCAGGTTTCCCCCATGCTTTTTGATGCGTTTCTTACCTCTGGTTTCCATGAGCCGGTGTCCACGCTCGGTGACGAAAAGGCTGTCCGTGTCCCCGTATACAGACTTGAGAGGGAGTTTAAACGAATTCAATAGCTTGTTGCATGGGCGGCGATATAAAATGCAAGGTGCTGCTCAAAAAATCAGGCAAAGCCTCGCGCAAAAAAGAAAGCACATCGTAGTCATGCTCATGCAGATAAAGGCAGGTAAGCTCCGGAACCACCACAGAAAAAGACACCATTTTTCTCTCAAACATGTCTGCGGGTTTCTGCATAAACACAAAATAAAATAACAAAAAAACATTTAAACATTAGAAGCCTGTCTTACAACAGGAAAAACAACCCTTATAAGCATAAGACGGACTACGGCCATGCCGGCGTGACCGTAAAAAAACTGGTCACCGTGATTAAAAAGCACCACCGACAGCTCCTCGGTCATGTCCGGAGTCATAATGTAAGACTCGGTAAACACATCAGGTTGATTCACATCGGTCAGTGCTAAAAAGCGACCGAAATAGCCCGGGGGAATACATACCCGCAGGCGTAGAGACAACATTACAGCCCCCATAGGAGGTATAACAAAATTAATAGGAGAGAAAAACACATAAACACCTGAAAAACCCTCCTGCCTAGGCAAAATAGCACCCTCCCGCTCCAGAACAACATACAGCGCTTCCACAGCGGCAGCCATAACAGTCAGCCTTACCAGTAAAAAAGAAAACCTATTAAAAAAACACCACTCGACACGGCACCAGCTCAATCAGTCACAGTGTAAAAAAGGGCCAAGTGCAGAGCGAGTATATATAGGACTAAAAAATGACGTAACGGTTAAAGTCCACAAAAAACACCCAGAAAACCGCACGCGAACCTACGCCCAGAAACGAAAGCCAAAAAACCCACAACTTCCTCAAATCGTCACTTCCGTTTTCCCACGTTACGTCACTTCCCATTTTAAGAAAACTACAATTCCCAACACATACAAGTTACTCCGCCCTAAAACCTACGTCACCCGCCCCGTTCCCACGCCCCGCGCCACGTCACAAACTCCACCCCCTCATTATCATATTGGCTTCAATCCAAAATAAGGTATATTATTGATGATGTTAATTAACATGCATGGATCCATATGCGGTGTGAAATACCGCACAGATGCGTAAGGAGAAAATACCGCATCAGGCGCTCTTCCGCTTCCTCGCTCACTGACTCGCTGCGCTCGGTCGTTCGGCTGCGGCGAGCGGTATCAGCTCACTCAAAGGCGGTAATACGGTTATCCACAGAATCAGGGGATAACGCAGGAAAGAACATGTGAGCAAAAGGCCAGCAAAAGGCCAGGAACCGTAAAAAGGCCGCGTTGCTGGCGTT

Supplementary Fig. 14 Map and nucleotide sequence of the AdV shuttle plasmid AQ08_pAd.Shu.MCS.

ITR, human adenovirus serotype-5 inverted terminal repeat; Ad5 Ψ, human adenovirus serotype-5 packaging signal; MCS, multiple cloning site; dark magenta segments, human adenovirus serotype-5 sequences involved in the HR-mediated assembly of full-length, fiber 50-encoding, AdV molecular clones in BJ5183^pAdEasy-2.50^ cells; E4ORF1, *E4ORF1* from human adenovirus serotype-5; ori, prokaryotic origin of replication; NeoR/KanR, *aminoglycoside phosphotransferase* gene from Tn5 conferring resistance to neomycin and kanamycin.


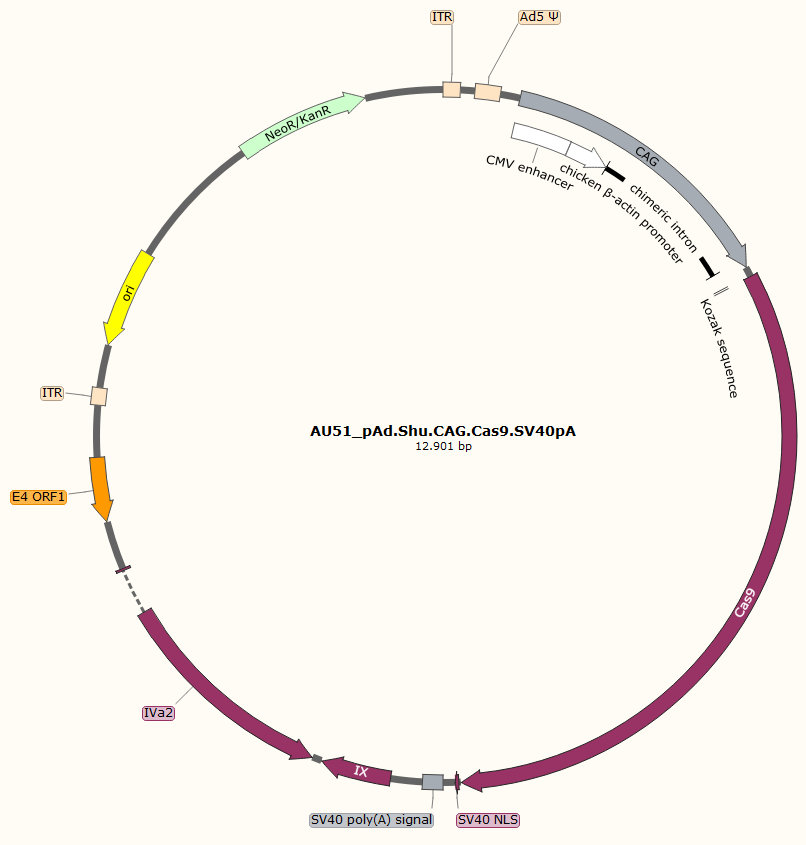


AU51_pAd.Shu.CAG.Cas9.SV40pA

CATCATCAATAATATACCTTATTTTGGATTGAAGCCAATATGATAATGAGGGGGTGGAGTTTGTGACGTGGCGCGGGGCGTGGGAACGGGGCGGGTGACGTAGTAGTGTGGCGGAAGTGTGATGTTGCAAGTGTGGCGGAACACATGTAAGCGACGGATGTGGCAAAAGTGACGTTTTTGGTGTGCGCCGGTGTACACAGGAAGTGACAATTTTCGCGCGGTTTTAGGCGGATGTTGTAGTAAATTTGGGCGTAACCGAGTAAGATTTGGCCATTTTCGCGGGAAAACTGAATAAGAGGAAGTGAAATCTGAATAATTTTGTGTTACTCATAGCGCGTAATACTGGTACCGGTTGTTAACGTTAGCCGGCTACGTATACTCCGGAATATTAATAGGCCTAGGATGCATATGGCGGCCGCCTGCAGCTGGCGCCATCGATACGCGTACTCGAGCCATGGATTCGACATTGATTATTGACTAGTTATTAATAGTAATCAATTACGGGGTCATTAGTTCATAGCCCATATATGGAGTTCCGCGTTACATAACTTACGGTAAATGGCCCGCCTGGCTGACCGCCCAACGACCCCCGCCCATTGACGTCAATAATGACGTATGTTCCCATAGTAACGCCAATAGGGACTTTCCATTGACGTCAATGGGTGGAGTATTTACGGTAAACTGCCCACTTGGCAGTACATCAAGTGTATCATATGCCAAGTACGCCCCCTATTGACGTCAATGACGGTAAATGGCCCGCCTGGCATTATGCCCAGTACATGACCTTATGGGACTTTCCTACTTGGCAGTACATCTACGTATTAGTCATCGCTATTACCATGGGTCGAGGTGAGCCCCACGTTCTGCTTCACTCTCCCCATCTCCCCCCCCTCCCCACCCCCAATTTTGTATTTATTTATTTTTTAATTATTTTGTGCAGCGATGGGGGCGGGGGGGGGGGGGGCGCGCGCCAGGCGGGGCGGGGCGGGGCGAGGGGCGGGGCGGGGCGAGGCGGAGAGGTGCGGCGGCAGCCAATCAGAGCGGCGCGCTCCGAAAGTTTCCTTTTATGGCGAGGCGGCGGCGGCGGCGGCCCTATAAAAAGCGAAGCGCGCGGCGGGCGGGAGTCGCTGCGTTGCCTTCGCCCCGTGCCCCGCTCCGCGCCGCCTCGCGCCGCCCGCCCCGGCTCTGACTGACCGCGTTACTCCCACAGGTGAGCGGGCGGGACGGCCCTTCTCCTCCGGGCTGTAATTAGCGCTTGGTTTAATGACGGCTCGTTTCTTTTCTGTGGCTGCGTGAAAGCCTTAAAGGGCTCCGGGAGGGCCCTTTGTGCGGGGGGGAGCGGCTCGGGGGGTGCGTGCGTGTGTGTGTGCGTGGGGAGCGCCGCGTGCGGCCCGCGCTGCCCGGCGGCTGTGAGCGCTGCGGGCGCGGCGCGGGGCTTTGTGCGCTCCGCGTGTGCGCGAGGGGAGCGCGGCCGGGGGCGGTGCCCCGCGGTGCGGGGGGGCTGCGAGGGGAACAAAGGCTGCGTGCGGGGTGTGTGCGTGGGGGGGTGAGCAGGGGGTGTGGGCGCGGCGGTCGGGCTGTAACCCCCCCCTGCACCCCCCTCCCCGAGTTGCTGAGCACGGCCCGGCTTCGGGTGCGGGGCTCCGTGCGGGGCGTGGCGCGGGGCTCGCCGTGCCGGGCGGGGGGTGGCGGCAGGTGGGGGTGCCGGGCGGGGCGGGGCCGCCTCGGGCCGGGGAGGGCTCGGGGGAGGGGCGCGGCGGCCCCGGAGCGCCGGCGGCTGTCGAGGCGCGGCGAGCCGCAGCCATTGCCTTTTATGGTAATCGTGCGAGAGGGCGCAGGGACTTCCTTTGTCCCAAATCTGGCGGAGCCGAAATCTGGGAGGCGCCGCCGCACCCCCTCTAGCGGGCGCGGGCGAAGCGGTGCGGCGCCGGCAGGAAGGAAATGGGCGGGGAGGGCCTTCGTGCGTCGCCGCGCCGCCGTCCCCTTCTCCATCTCCAGCCTCGGGGCTGCCGCAGGGGGACGGCTGCCTTCGGGGGGGACGGGGCAGGGCGGGGTTCGGCTTCTGGCGTGTGACCGGCGGCTCTAGAGCCTCTGCTAACCATGTTCATGCCTTCTTCTTTTTCCTACAGCTCCTGGGCAACGTGCTGGTTGTTGTGCTGTCTCATCATTTTGGCAAAGAATTATCGCATGCCTGCAGAGCTCTAGAGTCGTACGTCGCTAGAGGATCGAACCCTTGCCACCATGGACAAGAAGTACTCCATTGGGCTCGATATCGGCACAAACAGCGTCGGCTGGGCCGTCATTACGGACGAGTACAAGGTGCCGAGCAAAAAATTCAAAGTTCTGGGCAATACCGATCGCCACAGCATAAAGAAGAACCTCATTGGCGCCCTCCTGTTCGACTCCGGGGAGACGGCCGAAGCCACGCGGCTCAAAAGAACAGCACGGCGCAGATATACCCGCAGAAAGAATCGGATCTGCTACCTGCAGGAGATCTTTAGTAATGAGATGGCTAAGGTGGATGACTCTTTCTTCCATAGGCTGGAGGAGTCCTTTTTGGTGGAGGAGGATAAAAAGCACGAGCGCCACCCAATCTTTGGCAATATCGTGGACGAGGTGGCGTACCATGAAAAGTACCCAACCATATATCATCTGAGGAAGAAGCTTGTAGACAGTACTGATAAGGCTGACTTGCGGTTGATCTATCTCGCGCTGGCGCATATGATCAAATTTCGGGGACACTTCCTCATCGAGGGGGACCTGAACCCAGACAACAGCGATGTCGACAAACTCTTTATCCAACTGGTTCAGACTTACAATCAGCTTTTCGAAGAGAACCCGATCAACGCATCCGGAGTTGACGCCAAAGCAATCCTGAGCGCTAGGCTGTCCAAATCCCGGCGGCTCGAAAACCTCATCGCACAGCTCCCTGGGGAGAAGAAGAACGGCCTGTTTGGTAATCTTATCGCCCTGTCACTCGGGCTGACCCCCAACTTTAAATCTAACTTCGACCTGGCCGAAGATGCCAAGCTTCAACTGAGCAAAGACACCTACGATGATGATCTCGACAATCTGCTGGCCCAGATCGGCGACCAGTACGCAGACCTTTTTTTGGCGGCAAAGAACCTGTCAGACGCCATTCTGCTGAGTGATATTCTGCGAGTGAACACGGAGATCACCAAAGCTCCGCTGAGCGCTAGTATGATCAAGCGCTATGATGAGCACCACCAAGACTTGACTTTGCTGAAGGCCCTTGTCAGACAGCAACTGCCTGAGAAGTACAAGGAAATTTTCTTCGATCAGTCTAAAAATGGCTACGCCGGATACATTGACGGCGGAGCAAGCCAGGAGGAATTTTACAAATTTATTAAGCCCATCTTGGAAAAAATGGACGGCACCGAGGAGCTGCTGGTAAAGCTTAACAGAGAAGATCTGTTGCGCAAACAGCGCACTTTCGACAATGGAAGCATCCCCCACCAGATTCACCTGGGCGAACTGCACGCTATCCTCAGGCGGCAAGAGGATTTCTACCCCTTTTTGAAAGATAACAGGGAAAAGATTGAGAAAATCCTCACATTTCGGATACCCTACTATGTAGGCCCCCTCGCCCGGGGAAATTCCAGATTCGCGTGGATGACTCGCAAATCAGAAGAGACCATCACTCCCTGGAACTTCGAGGAAGTCGTGGATAAGGGGGCCTCTGCCCAGTCCTTCATCGAAAGGATGACTAACTTTGATAAAAATCTGCCTAACGAAAAGGTGCTTCCTAAACACTCTCTGCTGTACGAGTACTTCACAGTTTATAACGAGCTCACCAAGGTCAAATACGTCACAGAAGGGATGAGAAAGCCAGCATTCCTGTCTGGAGAGCAGAAGAAAGCTATCGTGGACCTCCTCTTCAAGACGAACCGGAAAGTTACCGTGAAACAGCTCAAAGAAGACTATTTCAAAAAGATTGAATGTTTCGACTCTGTTGAAATCAGCGGAGTGGAGGATCGCTTCAACGCATCCCTGGGAACGTATCACGATCTCCTGAAAATCATTAAAGACAAGGACTTCCTGGACAATGAGGAGAACGAGGACATTCTTGAGGACATTGTCCTCACCCTTACGTTGTTTGAAGATAGGGAGATGATTGAAGAACGCTTGAAAACTTACGCTCATCTCTTCGACGACAAAGTCATGAAACAGCTCAAGAGGCGCCGATATACAGGATGGGGGCGGCTGTCAAGAAAACTGATCAATGGGATCCGAGACAAGCAGAGTGGAAAGACAATCCTGGATTTTCTTAAGTCCGATGGATTTGCCAACCGGAACTTCATGCAGTTGATCCATGATGACTCTCTCACCTTTAAGGAGGACATCCAGAAAGCACAAGTTTCTGGCCAGGGGGACAGTCTTCACGAGCACATCGCTAATCTTGCAGGTAGCCCAGCTATCAAAAAGGGAATACTGCAGACCGTTAAGGTCGTGGATGAACTCGTCAAAGTAATGGGAAGGCATAAGCCCGAGAATATCGTTATCGAGATGGCCCGAGAGAACCAAACTACCCAGAAGGGACAGAAGAACAGTAGGGAAAGGATGAAGAGGATTGAAGAGGGTATAAAAGAACTGGGGTCCCAAATCCTTAAGGAACACCCAGTTGAAAACACCCAGCTTCAGAATGAGAAGCTCTACCTGTACTACCTGCAGAACGGCAGGGACATGTACGTGGATCAGGAACTGGACATCAATCGGCTCTCCGACTACGACGTGGATCATATCGTGCCCCAGTCTTTTCTCAAAGATGATTCTATTGATAATAAAGTGTTGACAAGATCCGATAAAAATAGAGGGAAGAGTGATAACGTCCCCTCAGAAGAAGTTGTCAAGAAAATGAAAAATTATTGGCGGCAGCTGCTGAACGCCAAACTGATCACACAACGGAAGTTCGATAATCTGACTAAGGCTGAACGAGGTGGCCTGTCTGAGTTGGATAAAGCCGGCTTCATCAAAAGGCAGCTTGTTGAGACACGCCAGATCACCAAGCACGTGGCCCAAATTCTCGATTCACGCATGAACACCAAGTACGATGAAAATGACAAACTGATTCGAGAGGTGAAAGTTATTACTCTGAAGTCTAAGCTGGTCTCAGATTTCAGAAAGGACTTTCAGTTTTATAAGGTGAGAGAGATCAACAATTACCACCATGCGCATGATGCCTACCTGAATGCAGTGGTAGGCACTGCACTTATCAAAAAATATCCCAAGCTTGAATCTGAATTTGTTTACGGAGACTATAAAGTGTACGATGTTAGGAAAATGATCGCAAAGTCTGAGCAGGAAATAGGCAAGGCCACCGCTAAGTACTTCTTTTACAGCAATATTATGAATTTTTTCAAGACCGAGATTACACTGGCCAATGGAGAGATTCGGAAGCGACCACTTATCGAAACAAACGGAGAAACAGGAGAAATCGTGTGGGACAAGGGTAGGGATTTCGCGACAGTCCGGAAGGTCCTGTCCATGCCGCAGGTGAACATCGTTAAAAAGACCGAAGTACAGACCGGAGGCTTCTCCAAGGAAAGTATCCTCCCGAAAAGGAACAGCGACAAGCTGATCGCACGCAAAAAAGATTGGGACCCCAAGAAATACGGCGGATTCGATTCTCCTACAGTCGCTTACAGTGTACTGGTTGTGGCCAAAGTGGAGAAAGGGAAGTCTAAAAAACTCAAAAGCGTCAAGGAACTGCTGGGCATCACAATCATGGAGCGATCAAGCTTCGAAAAAAACCCCATCGACTTTCTCGAGGCGAAAGGATATAAAGAGGTCAAAAAAGACCTCATCATTAAGCTTCCCAAGTACTCTCTCTTTGAGCTTGAAAACGGCCGGAAACGAATGCTCGCTAGTGCGGGCGAGCTGCAGAAAGGTAACGAGCTGGCACTGCCCTCTAAATACGTTAATTTCTTGTATCTGGCCAGCCACTATGAAAAGCTCAAAGGGTCTCCCGAAGATAATGAGCAGAAGCAGCTGTTCGTGGAACAACACAAACACTACCTTGATGAGATCATCGAGCAAATAAGCGAATTCTCCAAAAGAGTGATCCTCGCCGACGCTAACCTCGATAAGGTGCTTTCTGCTTACAATAAGCACAGGGATAAGCCCATCAGGGAGCAGGCAGAAAACATTATCCACTTGTTTACTCTGACCAACTTGGGCGCGCCTGCAGCCTTCAAGTACTTCGACACCACCATAGACAGAAAGCGGTACACCTCTACAAAGGAGGTCCTGGACGCCACACTGATTCATCAGTCAATTACGGGGCTCTATGAAACAAGAATCGACCTCTCTCAGCTCGGTGGAGACAGCAGGGCTGACCCCAAGAAGAAGAGGAAGGTGTGAAAGGGTTCGATCCCTACCGGCGACCGCGGACATGTACAGAGCTCGAGCGGCCGCTTCGAGCAGACATGATAAGATACATTGATGAGTTTGGACAAACCACAACTAGAATGCAGTGAAAAAAATGCTTTATTTGTGAAATTTGTGATGCTATTGCTTTATTTGTAACCATTATAAGCTGCAATAAACAAGTTAACAACAACAATTGCATTCATTTTATGTTTCAGGTTCAGGGGGAGGTGTGGGAGGTTTTTTAAAGCAAGTAAAACCTCTACAAATGTGGTAAAATCGATAAGGATCTGGGCGTGGTTAAGGGTGGGAAAGAATATATAAGGTGGGGGTCTTATGTAGTTTTGTATCTGTTTTGCAGCAGCCGCCGCCGCCATGAGCACCAACTCGTTTGATGGAAGCATTGTGAGCTCATATTTGACAACGCGCATGCCCCCATGGGCCGGGGTGCGTCAGAATGTGATGGGCTCCAGCATTGATGGTCGCCCCGTCCTGCCCGCAAACTCTACTACCTTGACCTACGAGACCGTGTCTGGAACGCCGTTGGAGACTGCAGCCTCCGCCGCCGCTTCAGCCGCTGCAGCCACCGCCCGCGGGATTGTGACTGACTTTGCTTTCCTGAGCCCGCTTGCAAGCAGTGCAGCTTCCCGTTCATCCGCCCGCGATGACAAGTTGACGGCTCTTTTGGCACAATTGGATTCTTTGACCCGGGAACTTAATGTCGTTTCTCAGCAGCTGTTGGATCTGCGCCAGCAGGTTTCTGCCCTGAAGGCTTCCTCCCCTCCCAATGCGGTTTAAAACATAAATAAAAAACCAGACTCTGTTTGGATTTGGATCAAGCAAGTGTCTTGCTGTCTTTATTTAGGGGTTTTGCGCGCGCGGTAGGCCCGGGACCAGCGGTCTCGGTCGTTGAGGGTCCTGTGTATTTTTTCCAGGACGTGGTAAAGGTGACTCTGGATGTTCAGATACATGGGCATAAGCCCGTCTCTGGGGTGGAGGTAGCACCACTGCAGAGCTTCATGCTGCGGGGTGGTGTTGTAGATGATCCAGTCGTAGCAGGAGCGCTGGGCGTGGTGCCTAAAAATGTCTTTCAGTAGCAAGCTGATTGCCAGGGGCAGGCCCTTGGTGTAAGTGTTTACAAAGCGGTTAAGCTGGGATGGGTGCATACGTGGGGATATGAGATGCATCTTGGACTGTATTTTTAGGTTGGCTATGTTCCCAGCCATATCCCTCCGGGGATTCATGTTGTGCAGAACCACCAGCACAGTGTATCCGGTGCACTTGGGAAATTTGTCATGTAGCTTAGAAGGAAATGCGTGGAAGAACTTGGAGACGCCCTTGTGACCTCCAAGATTTTCCATGCATTCGTCCATAATGATGGCAATGGGCCCACGGGCGGCGGCCTGGGCGAAGATATTTCTGGGATCACTAACGTCATAGTTGTGTTCCAGGATGAGATCGTCATAGGCCATTTTTACAAAGCGCGGGCGGAGGGTGCCAGACTGCGGTATAATGGTTCCATCCGGCCCAGGGGCGTAGTTACCCTCACAGATTTGCATTTCCCACGCTTTGAGTTCAGATGGGGGGATCATGTCTACCTGCGGGGCGATGAAGAAAACGGTTTCCGGGGTAGGGGAGATCAGCTGGGAAGAAAGCAGGTTCCTGAGCAGCTGCGACTTACCGCAGCCGGTGGGCCCGTAAATCACACCTATTACCGGGTGCAACTGGTAGTTAAGAGAGCTGCAGCTGCCGTCATCCCTGAGCAGGGGGGCCACTTCGTTAAGCATGTCCCTGACTCGCATGTTTTCCCTGACCAAATCCGCCAGAAGGCGCTCGCCGCCCAGCGATAGCAGTTCTTGCAAGGAAGCAAAGTTTTTCAACGGTTTGAGACCGTCCGCCGTAGGCATGCTTTTGAGCGTTTGACCAAGCAGTTCCAGGCGGTCCCACAGCTCGGTCACCTGCTCTACGGCATCTCGATCCAGCATATCTCCTCGTTTCGCGGGTTGGGGCGGCTTTCGCTGTACGGCAGTAGTCGGTGCTCGTCCAGACGGGCCAGGGTCATGTCTTTCCACGGGCGCAGGGTCCTCGTCAGCGTAGTCTGGGTCACGGTGAAGGGGTGCGCTCCGGGCTGCGCGCTGGCCAGGGTGCGCTTGAGGCTGGTCCTGCTGGTGCTGAAGCGCTGCCGGTCTTCGCCCTGCGCGTCGGCCAGGTAGCATTTGACCATGGTGTCATAGTCCAGCCCCTCCGCGGCGTGGCCCTTGGCGCGCAGCTTGCCCTTGGAGGAGGCGCCGCACGAGGGGCAGTGCAGACTTTTGAGGGCGTAGAGCTTGGGCGCGAGAAATACCGATTCCGGGGAGTAGGCATCCGCGCCGCAGGCCCCGCAGACGGTCTCGCATTCCACGAGCCAGGTGAGCTCTGGCCGTTCGGGGTCAAAAACCAGGTTTCCCCCATGCTTTTTGATGCGTTTCTTACCTCTGGTTTCCATGAGCCGGTGTCCACGCTCGGTGACGAAAAGGCTGTCCGTGTCCCCGTATACAGACTTGAGAGGGAGTTTAAACGAATTCAATAGCTTGTTGCATGGGCGGCGATATAAAATGCAAGGTGCTGCTCAAAAAATCAGGCAAAGCCTCGCGCAAAAAAGAAAGCACATCGTAGTCATGCTCATGCAGATAAAGGCAGGTAAGCTCCGGAACCACCACAGAAAAAGACACCATTTTTCTCTCAAACATGTCTGCGGGTTTCTGCATAAACACAAAATAAAATAACAAAAAAACATTTAAACATTAGAAGCCTGTCTTACAACAGGAAAAACAACCCTTATAAGCATAAGACGGACTACGGCCATGCCGGCGTGACCGTAAAAAAACTGGTCACCGTGATTAAAAAGCACCACCGACAGCTCCTCGGTCATGTCCGGAGTCATAATGTAAGACTCGGTAAACACATCAGGTTGATTCACATCGGTCAGTGCTAAAAAGCGACCGAAATAGCCCGGGGGAATACATACCCGCAGGCGTAGAGACAACATTACAGCCCCCATAGGAGGTATAACAAAATTAATAGGAGAGAAAAACACATAAACACCTGAAAAACCCTCCTGCCTAGGCAAAATAGCACCCTCCCGCTCCAGAACAACATACAGCGCTTCCACAGCGGCAGCCATAACAGTCAGCCTTACCAGTAAAAAAGAAAACCTATTAAAAAAACACCACTCGACACGGCACCAGCTCAATCAGTCACAGTGTAAAAAAGGGCCAAGTGCAGAGCGAGTATATATAGGACTAAAAAATGACGTAACGGTTAAAGTCCACAAAAAACACCCAGAAAACCGCACGCGAACCTACGCCCAGAAACGAAAGCCAAAAAACCCACAACTTCCTCAAATCGTCACTTCCGTTTTCCCACGTTACGTCACTTCCCATTTTAAGAAAACTACAATTCCCAACACATACAAGTTACTCCGCCCTAAAACCTACGTCACCCGCCCCGTTCCCACGCCCCGCGCCACGTCACAAACTCCACCCCCTCATTATCATATTGGCTTCAATCCAAAATAAGGTATATTATTGATGATGTTAATTAACATGCATGGATCCATATGCGGTGTGAAATACCGCACAGATGCGTAAGGAGAAAATACCGCATCAGGCGCTCTTCCGCTTCCTCGCTCACTGACTCGCTGCGCTCGGTCGTTCGGCTGCGGCGAGCGGTATCAGCTCACTCAAAGGCGGTAATACGGTTATCCACAGAATCAGGGGATAACGCAGGAAAGAACATGTGAGCAAAAGGCCAGCAAAAGGCCAGGAACCGTAAAAAGGCCGCGTTGCTGGCGTTTTTCCATAGGCTCCGCCCCCCTGACGAGCATCACAAAAATCGACGCTCAAGTCAGAGGTGGCGAAACCCGACAGGACTATAAAGATACCAGGCGTTTCCCCCTGGAAGCTCCCTCGTGCGCTCTCCTGTTCCGACCCTGCCGCTTACCGGATACCTGTCCGCCTTTCTCCCTTCGGGAAGCGTGGCGCTTTCTCATAGCTCACGCTGTAGGTATCTCAGTTCGGTGTAGGTCGTTCGCTCCAAGCTGGGCTGTGTGCACGAACCCCCCGTTCAGCCCGACCGCTGCGCCTTATCCGGTAACTATCGTCTTGAGTCCAACCCGGTAAGACACGACTTATCGCCACTGGCAGCAGCCACTGGTAACAGGATTAGCAGAGCGAGGTATGTAGGCGGTGCTACAGAGTTCTTGAAGTGGTGGCCTAACTACGGCTACACTAGAAGGACAGTATTTGGTATCTGCGCTCTGCTGAAGCCAGTTACCTTCGGAAAAAGAGTTGGTAGCTCTTGATCCGGCAAACAAACCACCGCTGGTAGCGGTGGTTTTTTTGTTTGCAAGCAGCAGATTACGCGCAGAAAAAAAGGATCTCAAGAAGATCCTTTGATCTTTTCTACGGGGTCTGACGCTCAGTGGAACGAAAACTCACGTTAAGGGATTTTGGTCATGAGATTATCAAAAAGGATCTTCACCTAGATCCTTTTAAATTAAAAATGAAGTTTTAAATCAATCTAAAGTATATATGAGTAAACTTGGTCTGACAGTTACCAATGCTTAATCAGTGAGGCACCTATCTCAGCGATCTGTCTATTTCGTTCATCCATAGTTGCCTGACTCCCCGTCGTGTAGATAACTACGATACGGGAGGGCTTACCATCTGGCCCCAGTGCTGCAATGATACCGCGAGACCCACGCTCACCGGCTCCAGATTTATCAGCAATAAACCAGCCAGCCGGAAGGGCCGAGCGCAGAAGTGGTCCTGCAACTTTATCCGCCTCCATCCAGTCTATTAATTGTTGCCGGGAAGCTAGAGTAAGTAGTTCGCCAGTTAATAGTTTGCGCAACGTTGTTGCCATTGCTGCAGCCATGAGATTATCAAAAAGGATCTTCACCTAGATCCTTTTCACGTAGAAAGCCAGTCCGCAGAAACGGTGCTGACCCCGGATGAATGTCAGCTACTGGGCTATCTGGACAAGGGAAAACGCAAGCGCAAAGAGAAAGCAGGTAGCTTGCAGTGGGCTTACATGGCGATAGCTAGACTGGGCGGTTTTATGGACAGCAAGCGAACCGGAATTGCCAGCTGGGGCGCCCTCTGGTAAGGTTGGGAAGCCCTGCAAAGTAAACTGGATGGCTTTCTTGCCGCCAAGGATCTGATGGCGCAGGGGATCAAGCTCTGATCAAGAGACAGGATGAGGATCGTTTCGCATGATTGAACAAGATGGATTGCACGCAGGTTCTCCGGCCGCTTGGGTGGAGAGGCTATTCGGCTATGACTGGGCACAACAGACAATCGGCTGCTCTGATGCCGCCGTGTTCCGGCTGTCAGCGCAGGGGCGCCCGGTTCTTTTTGTCAAGACCGACCTGTCCGGTGCCCTGAATGAACTGCAAGACGAGGCAGCGCGGCTATCGTGGCTGGCCACGACGGGCGTTCCTTGCGCAGCTGTGCTCGACGTTGTCACTGAAGCGGGAAGGGACTGGCTGCTATTGGGCGAAGTGCCGGGGCAGGATCTCCTGTCATCTCACCTTGCTCCTGCCGAGAAAGTATCCATCATGGCTGATGCAATGCGGCGGCTGCATACGCTTGATCCGGCTACCTGCCCATTCGACCACCAAGCGAAACATCGCATCGAGCGAGCACGTACTCGGATGGAAGCCGGTCTTGTCGATCAGGATGATCTGGACGAAGAGCATCAGGGGCTCGCGCCAGCCGAACTGTTCGCCAGGCTCAAGGCGAGCATGCCCGACGGCGAGGATCTCGTCGTGACCCATGGCGATGCCTGCTTGCCGAATATCATGGTGGAAAATGGCCGCTTTTCTGGATTCATCGACTGTGGCCGGCTGGGTGTGGCGGACCGCTATCAGGACATAGCGTTGGCTACCCGTGATATTGCTGAAGAGCTTGGCGGCGAATGGGCTGACCGCTTCCTCGTGCTTTACGGTATCGCCGCTCCCGATTCGCAGCGCATCGCCTTCTATCGCCTTCTTGACGAGTTCTTCTGAATTTTGTTAAAATTTTTGTTAAATCAGCTCATTTTTTAACCAATAGGCCGAAATCGGCAAAATCCCTTATAAATCAAAAGAATAGACCGAGATAGGGTTGAGTGTTGTTCCAGTTTGGAACAAGAGTCCACTATTAAAGAACGTGGACTCCAACGTCAAAGGGCGAAAAACCGTCTATCAGGGCGATGGCCCACTACGTGAACCATCACCCTAATCAAGTTTTTTGGGGTCGAGGTGCCGTAAAGCACTAAATCGGAACCCTAAAGGGAGCCCCCGATTTAGAGCTTGACGGGGAAAGCCGGCGAACGTGGCGAGAAAGGAAGGGAAGAAAGCGAAAGGAGCGGGCGCTAGGGCGCTGGCAAGTGTAGCGGTCACGCTGCGCGTAACCACCACACCCGCCGCGCTTAATGCGCCGCTACAGGGCGCGTCCATTCGCCATTCAGGATCGAATTAATTCTTAATTAA

Supplementary Fig. 15 Map and nucleotide sequence of AU51_ pAd.shu.CAG.Cas9.SV40pA.

ITR, human adenovirus serotype-5 inverted terminal repeat; Ad5Ψ, human adenovirus serotype-5 packaging signal; CAG, CMV *immediate-early* enhancer/chicken *β-actin* promoter; Cas9, Cas9 endonuclease from the type II CRISPR-Cas system from *Streptococcus pyogenes;* SV40 NLS, nuclear localization signal from SV40 large T antigen; SV40 poly(A) signal, SV40 polyadenylation signal; IX and IVa2 segments, human adenovirus serotype-5 sequences involved in the HR-mediated assembly of full-length, fiber 50-encoding, AdV molecular clones in BJ5183^pAdEasy-2.50^ cells; E4ORF1, *E4ORF1* from human adenovirus serotype-5; ori, prokaryotic origin of replication; NeoR/KanR, *aminoglycoside phosphotransferase* gene from Tn5 conferring resistance to neomycin and kanamycin.

**Supplementary Table 1. Data points of the experiments presented in Fig. 3**

| **A** | Cas9 Neg +  gRNA^eGFP.16^ | Cas9 Neg + opt-gRNA^eGFP.16^ | Cas9 +  gRNA^eGFP.16^ | Cas9 + opt-gRNA^eGFP.16^ | eCas9.2NLS +  gRNA^eGFP.16^ | eCas9.2NLS + opt-gRNA^eGFP.16^ | eCas9.4NLS +  gRNA^eGFP.16^ | eCas9.4NLS + opt-gRNA^eGFP.16^ |
| --- | --- | --- | --- | --- | --- | --- | --- | --- |
| Experiment I | 0,47 | 0,60 | 56,12 | 63,61 | 56,43 | 64,90 | 59,11 | 63,39 |
| Experiment II | 0,39 | 0,49 | 55,25 | 60,74 | 52,18 | 59,42 | 55,16 | 63,54 |
| Experiment III | 0,48 | 0,47 | 55,49 | 59,68 | 52,08 | 57,39 | 53,16 | 57,77 |

| **B** | Cas9 Neg +  gRNA^eGFP.6^ | Cas9 Neg + opt-gRNA^eGFP.6^ | Cas9 +  gRNA^eGFP.6^ | Cas9 + opt-gRNA^eGFP.6^ | eCas9.2NLS +  gRNA^eGFP.6^ | eCas9.2NLS + opt-gRNA^eGFP.6^ | eCas9.4NLS +  gRNA^eGFP.6^ | eCas9.4NLS + opt-gRNA^eGFP.6^ |
| --- | --- | --- | --- | --- | --- | --- | --- | --- |
| Experiment I | 0,48 | 0,54 | 57,26 | 57,70 | 63,79 | 59,03 | 67,46 | 69,07 |
| Experiment II | 0,29 | 0,28 | 56,56 | 59,24 | 61,11 | 59,63 | 63,89 | 64,67 |
| Experiment III | 0,61 | 0,53 | 44,85 | 50,79 | 55,61 | 56,05 | 54,09 | 58,01 |

| **C** | Cas9 Neg +  gRNA^eGFP.16^ | Cas9 Neg + opt-gRNA^eGFP.16^ | Cas9 +  gRNA^eGFP.16^ | Cas9 + opt-gRNA^eGFP.16^ | eCas9.2NLS +  gRNA^eGFP.16^ | eCas9.2NLS + opt-gRNA^eGFP.16^ | eCas9.4NLS +  gRNA^eGFP.16^ | eCas9.4NLS + opt-gRNA^eGFP.16^ |
| --- | --- | --- | --- | --- | --- | --- | --- | --- |
| Experiment I | 0,08 | 0,07 | 0,08 | 0,06 | 0,08 | 0,08 | 0,09 | 0,08 |
| Experiment II | 0,06 | 0,07 | 0,11 | 0,14 | 0,16 | 0,15 | 0,16 | 0,07 |
| Experiment III | 0,03 | 0,04 | 0,03 | 0,03 | 0,04 | 0,03 | 0,02 | 0,05 |

| **D** | Cas9 Neg +  gRNA^eGFP.3^ | Cas9 Neg + opt-gRNA^eGFP.3^ | Cas9 +  gRNA^eGFP.3^ | Cas9 + opt-gRNA^eGFP.3^ | eCas9.2NLS +  gRNA^eGFP.3^ | eCas9.2NLS + opt-gRNA^eGFP.3^ | eCas9.4NLS +  gRNA^eGFP.3^ | eCas9.4NLS + opt-gRNA^eGFP.3^ |
| --- | --- | --- | --- | --- | --- | --- | --- | --- |
| Experiment I | 0,06 | 0,06 | 41,57 | 46,06 | 0,03 | 0,05 | 0,05 | 0,04 |
| Experiment II | 0,08 | 0,08 | 42,25 | 45,79 | 0,17 | 0,12 | 0,17 | 0,11 |
| Experiment III | 0,07 | 0,05 | 42,6 | 48,31 | 0,05 | 0,05 | 0,07 | 0,05 |

A) Values correspond to the percentage of *eGFP* knockout in the experiments presented in Fig. 3A.

B) Values correspond to the percentage of *eGFP* knockout in the experiments presented in Fig. 3B.

C) Values correspond to the percentage of *mTurquoise2* knockout in the experiments presented in Fig. 3C.

D) Values correspond to the percentage of *mTurquoise2* knockout in the experiments presented in Fig. 3D.

**Supplementary Table 2. Data points of the experiments presented in Supplementary Fig. 2**

|  | Cas9 Neg +  gRNA^eGFP.5^ | Cas9 Neg + opt-gRNA^eGFP.5^ | Cas9 +  gRNA^eGFP.5^ | Cas9 + opt-gRNA^eGFP.5^ | eCas9.2NLS +  gRNA^eGFP.5^ | eCas9.2NLS + opt-gRNA^eGFP.5^ | eCas9.4NLS +  gRNA^eGFP.5^ | eCas9.4NLS + opt-gRNA^eGFP.5^ |
| --- | --- | --- | --- | --- | --- | --- | --- | --- |
| Experiment I | 0,54 | 0,59 | 53,97 | 66,63 | 51,46 | 55,86 | 54,09 | 61,59 |
| Experiment II | 0,37 | 0,41 | 58,55 | 64,53 | 43,61 | 53,55 | 48,34 | 51,72 |
| Experiment III | 0,48 | 0,37 | 55,9 | 56,31 | 44,18 | 41,35 | 46,87 | 49,83 |

Values correspond to the percentage of *eGFP* knockout in the experiments presented in Supplementary Fig. 2.

**Supplementary Table 3. Data points of the experiments shown in Fig. 3e**

|  | | eGFP knockout levels corrected for initial transfection efficiency | | | |
| --- | --- | --- | --- | --- | --- |
|  |  | Exp. 1 | Exp. 2 | Exp. 3 | Exp. 4 |
| Cas9 | opt-gOT.1 | 67,44% | 57,14% | 71,48% | 64,31% |
|  | opt-gOT.2 | 65,16% | 54,93% | 66,17% | 70,39% |
|  | opt-gOT.3 | 65,70% | 57,72% | 69,57% | 66,77% |
|  | opt-gOT.4 | 60,98% | 64,19% | 71,84% | 68,00% |
|  | opt-gOT.5 | 65,05% | 60,42% | 66,30% | 64,43% |
|  | opt-gOT.6 | 29,89% | 21,87% | 26,59% | 27,95% |
|  | opt-gOT.7 | 67,45% | 60,60% | 71,01% | 63,09% |
|  | opt-gOT.8 | 60,04% | 53,52% | 57,91% | 57,46% |
|  | opt-gOT.9 | 8,03% | 8,87% | 9,62% | 8,76% |
|  | opt-gOT.10 | 27,17% | 21,88% | 22,42% | 23,68% |
|  | gNT | 2,25% | 3,02% | 3,52% | 4,05% |
| eCas9.2NLS | opt-gOT.1 | 23,55% | 21,63% | 22,69% | 25,04% |
|  | opt-gOT.2 | 59,07% | 55,60% | 68,77% | 65,92% |
|  | opt-gOT.3 | 45,02% | 39,13% | 48,43% | 47,48% |
|  | opt-gOT.4 | 41,28% | 36,76% | 42,16% | 34,42% |
|  | opt-gOT.5 | 3,34% | 5,33% | 5,26% | 4,15% |
|  | opt-gOT.6 | 1,88% | 4,19% | 4,85% | 3,41% |
|  | opt-gOT.7 | 25,58% | 21,79% | 25,22% | 24,35% |
|  | opt-gOT.8 | 55,24% | 54,96% | 57,19% | 58,77% |
|  | opt-gOT.9 | 2,92% | 4,24% | 2,31% | 2,86% |
|  | opt-gOT.10 | 2,54% | 3,56% | 4,98% | 3,82% |
|  | gNT | 2,26% | 3,37% | 4,01% | 2,20% |
| eCas9.4NLS | opt-gOT.1 | 28,54% | 30,12% | 34,33% | 30,06% |
|  | opt-gOT.2 | 63,54% | 58,47% | 74,20% | 68,83% |
|  | opt-gOT.3 | 49,75% | 48,59% | 52,23% | 49,39% |
|  | opt-gOT.4 | 42,07% | 45,85% | 46,96% | 49,09% |
|  | opt-gOT.5 | 3,35% | 5,84% | 4,10% | 5,12% |
|  | opt-gOT.6 | 2,71% | 4,67% | 3,57% | 3,37% |
|  | opt-gOT.7 | 28,30% | 30,69% | 29,54% | 28,40% |
|  | opt-gOT.8 | 57,14% | 60,92% | 62,97% | 58,05% |
|  | opt-gOT.9 | 2,26% | 5,38% | - | 3,40% |
|  | opt-gOT.10 | 1,72% | 4,10% | 2,59% | 2,86% |
|  | gNT | 1,88% | 5,83% | 3,85% | 2,72% |

**Supplementary Table 4. Data points of the experiments presented in Supplementary Fig. 3b**

| **gRNA^empty^** | Cas9 | | | eCas9.2NLS | | | eCas9.4NLS | | |
| --- | --- | --- | --- | --- | --- | --- | --- | --- | --- |
|  | Exp I | Exp II | Exp III | Exp I | Exp II | Exp III | Exp I | Exp II | Exp III |
| - DOX | 43,02 | 38,65 | 41,52 | 47,21 | 40,4 | 41,76 | 44,71 | 38,5 | 41,98 |
| + DOX | 44,83 | 39,51 | 43,05 | 47,26 | 42,1 | 43,72 | 45,29 | 39,05 | 44,4 |

| **gRNA^eGFP.8^** | Cas9 | | | eCas9.2NLS | | | eCas9.4NLS | | |
| --- | --- | --- | --- | --- | --- | --- | --- | --- | --- |
|  | Exp I | Exp II | Exp III | Exp I | Exp II | Exp III | Exp I | Exp II | Exp III |
| - DOX | 46,41 | 36,18 | 41,26 | 45,51 | 40,06 | 45,17 | 43,49 | 41,28 | 44,79 |
| + DOX | 45,87 | 38,54 | 42,8 | 49,89 | 40,09 | 45,76 | 44,12 | 39,53 | 44,64 |

| **gRNA^eGFP.9^** | Cas9 | | | eCas9.2NLS | | | eCas9.4NLS | | |
| --- | --- | --- | --- | --- | --- | --- | --- | --- | --- |
|  | Exp I | Exp II | Exp III | Exp I | Exp II | Exp III | Exp I | Exp II | Exp III |
| - DOX | 45,02 | 38,6 | 42,96 | 49,87 | 41,56 | 44,61 | 44,76 | 42,05 | 40,66 |
| + DOX | 43,37 | 39,06 | 45,58 | 47,08 | 41,69 | 45,84 | 45,34 | 42,45 | 42,15 |

Values correspond to the percentage of DsRed positive cells at 3 days post-transfection in HEK.EGFP^TetO.KRAB^ cells. Data are plotted in Supplementary Fig. 3b.

**Supplementary Table 5. Data points of the experiments presented in Supplementary Fig. 3c**

| **E**  **gRNA^empty^** | Cas9 | | | eCas9.2NLS | | | eCas9.4NLS | | |
| --- | --- | --- | --- | --- | --- | --- | --- | --- | --- |
|  | Exp I | Exp II | Exp III | Exp I | Exp II | Exp III | Exp I | Exp II | Exp III |
| - DOX | 4,01 | 2,15 | 1,48 | 4,61 | 1,67 | 1,39 | 4,26 | 1,84 | 1,47 |
| + DOX | 5,68 | 3,66 | 3,64 | 5,57 | 3,52 | 2,90 | 4,24 | 3,75 | 3,56 |

| **E**  **gRNA^eGFP.8^** | Cas9 | | | eCas9.2NLS | | | eCas9.4NLS | | |
| --- | --- | --- | --- | --- | --- | --- | --- | --- | --- |
|  | Exp I | Exp II | Exp III | Exp I | Exp II | Exp III | Exp I | Exp II | Exp III |
| - DOX | 22,14 | 24,79 | 18,69 | 25,29 | 36,43 | 26,39 | 30,1 | 40,18 | 26,53 |
| + DOX | 47,85 | 48,24 | 45,59 | 57,45 | 56,81 | 61,64 | 54,75 | 62,58 | 58,62 |

| **E**  **gRNA^eGFP.9^** | Cas9 | | | eCas9.2NLS | | | eCas9.4NLS | | |
| --- | --- | --- | --- | --- | --- | --- | --- | --- | --- |
|  | Exp I | Exp II | Exp III | Exp I | Exp II | Exp III | Exp I | Exp II | Exp III |
| - DOX | 27,69 | 44,19 | 28,12 | 35,54 | 46,51 | 32,41 | 34,93 | 51,46 | 29,51 |
| + DOX | 60,84 | 74,03 | 67,44 | 59,27 | 70,69 | 66,39 | 59,45 | 70,92 | 59,11 |

Values correspond to the percentage of *eGFP* knockout normalized for transfection efficiencies. Data are plotted in Supplementary Fig. 3c.

**Supplementary Table 6. Data points of the experiments presented in Fig. 4b**

|  | gRNA^eGFP.8^ | | | gRNA^eGFP.9^ | | |
| --- | --- | --- | --- | --- | --- | --- |
|  | Cas9 | eCas9.2NLS | eCas9.4NLS | Cas9 | eCas9.2NLS | eCas9.4NLS |
| Exp I | 2,16 | 2,27 | 1,82 | 2,20 | 1,67 | 1,70 |
| Exp II | 1,95 | 1,56 | 1,56 | 1,68 | 1,52 | 1,38 |
| Exp III | 2,44 | 2,34 | 2,21 | 2,40 | 2,05 | 2,00 |

Values represent the ratios between *eGFP* knockout levels at euchromatic (+ Dox) versus heterochromatic

(- Dox) target sequences presented in Fig. 4b.

**Supplementary Table 7.** **Data points of the experiments presented in Fig. 5**

| **A** | Cytoplasm | | | Nuclei | | |
| --- | --- | --- | --- | --- | --- | --- |
|  | Exp I | Exp II | Exp III | Exp I | Exp II | Exp III |
| AdV.Cas9 +AdV.gRNA^S1^ | 0,46 | 0,46 | 0,48 | 0,54 | 0,54 | 0,52 |
| AdV.eCas9.2NLS +AdV.gRNA^S1^ | 0,28 | 0,28 | 0,32 | 0,72 | 0,72 | 0,68 |
| AdV.eCas9.4NLS +AdV.gRNA^S1^ | 0,18 | 0,21 | 0,23 | 0,82 | 0,79 | 0,77 |
| AdV.Cas9 | 0,43 | 0,45 | 0,46 | 0,57 | 0,55 | 0,54 |
| AdV.eCas9.2NLS | 0,24 | 0,27 | 0,3 | 0,76 | 0,73 | 0,7 |
| AdV.eCas9.4NLS | 0,17 | 0,21 | 0,21 | 0,83 | 0,79 | 0,79 |

| **B** | AdV.Cas9 +AdV.gRNA^S1^ | AdV.eCas9.2NLS +AdV.gRNA^S1^ | AdV.eCas9.4NLS +AdV.gRNA^S1^ | AdV.Cas9 | AdV.eCas9.2NLS | AdV.eCas9.4NLS |
| --- | --- | --- | --- | --- | --- | --- |
| Experiment I | 4,45 | 7,45 | 17,4 | 4,33 | 8,80 | 16,2 |
| Experiment II | 3,38 | 4,81 | 11,4 | 3,47 | 5,15 | 10,3 |
| Experiment III | 4,08 | 6,4 | 13,7 | 4,51 | 6,43 | 11,6 |

A) Values correspond to the mean of nuclear or cytoplasmic Cas9-derived fluorescence intensity normalized, on a per-cell-basis, to the cellular fluorescence intensity. Data are plotted in Fig. 5b.

B) Values are calculated by normalizing geometric mean intensity (GMI) in the nucleus versus GMI in the cells. Data are potted in Fig. 5c.

**Supplementary Table 8. Data points of the experiments presented in Fig. 6a**

|  | AdV.gRNA^eGFP^ | | | AdV.Cas9 +AdV.gRNA^eGFP^ | | | AdV.eCas9.2NLS +AdV.gRNA^eGFP^ | | | AdV.eCas9.4NLS +AdV.gRNA^eGFP^ | | |
| --- | --- | --- | --- | --- | --- | --- | --- | --- | --- | --- | --- | --- |
| MOI | Exp I | Exp II | Exp III | Exp I | Exp II | Exp III | Exp I | Exp II | Exp III | Exp I | Exp II | Exp III |
| 2 | 0,73 | 0,65 | 0,66 | 3,89 | 4,29 | 2,93 | 2,62 | 2,69 | 1,90 | 6,73 | 7,61 | 6,53 |
| 6 | 0,54 | 0,51 | 0,60 | 22,25 | 21,32 | 14,74 | 12,19 | 16,26 | 10,24 | 28,86 | 31,24 | 24,51 |
| 20 | 0,89 | 0,63 | 0,89 | 57,24 | 64,17 | 53,31 | 47,09 | 60,21 | 46,27 | 68,56 | 74,30 | 62,79 |
| 60 | 0,99 | 0,35 | 0,65 | 88,99 | 90,32 | 86,62 | 89,26 | 91,09 | 84,38 | 91,03 | 93,22 | 88,86 |
| 200 | 0,70 | 0,37 | 0,50 | 97,87 | 98,23 | 96,91 | 98,4 | 98,79 | 97,42 | 98,02 | 98,82 | 97,27 |

Values correspond to the percentage of eGFP knockout in the experiments presented in Fig. 6a.

Supplementary Table 9. **Oligonucleotide pairs to generate gRNA expression constructs**

| Target site | Oligonucleotide code | Oligonucleotide sequence |
| --- | --- | --- |
| eGFP.3 | #163  #164 | 5’-ACCGTAGGTCAGGGTGGTCACGA-3’  5’-AAACTCGTGACCACCCTGACCTA-3’ |
| eGFP.5 | #171  #172 | 5’-ACCGCGAGGGCGATGCCACCTA-3’  5’-AAACTAGGTGGCATCGCCCTCG-3’ |
| eGFP.6 | #173  #174 | 5’-ACCGGCGAGGGCGATGCCACCTA-3’  5’-AAACTAGGTGGCATCGCCCTCGC-3’ |
| eGFP.16 | #203  #204 | 5’-ACCGCTCGTGACCACCCTGACCTA-3’  5’-AAACTAGGTCAGGGTGGTCACGAG-3’ |
| AAVS1-T2 | #97  #98 | 5’-ACCGGGGCCACTAGGGACAGGAT-3’  5’-AAACATCCTGTCCCTAGTGGCCC-3’ |

Supplementary Table 10. **Oligonucleotide pairs to generate constructs expressing opt-gRNAs**

**with different numbers and positions of mismatches to a *eGFP* target sequence**

| Plasmid name | gRNA name | Oligonucleotide codes | Oligonucleotide sequences (5’🡪3’) |
| --- | --- | --- | --- |
| AE71_pU6.opt-gRNA.OT.1 | opt-gOT.1 | #421  #422 | ACCGATCGCCCTCGAACTTCACCT  AAACAGGTGAAGTTCGAGGGCGAT |
| AE72_pU6.opt-gRNA.OT.2 | opt-gOT.2 | #423  #424 | ACCGCCGCCCTCGAACTTCACCT  AAACAGGTGAAGTTCGAGGGCGG |
| AE73_p.U6.opt-gRNA.OT.3 | opt-gOT.3 | #425  #426 | ACCGTTGCCCTCGAACTTCACCT  AAACAGGTGAAGTTCGAGGGCAA |
| AE74_pU6.opt-gRNA.OT.4 | opt-gOT.4 | #427  #428 | ACCGTCACCCTCGAACTTCACCT  AAACAGGTGAAGTTCGAGGGTGA |
| AN65_pU6.opt-gRNA.OT.5 | opt-gOT.5 | #447  #448 | ACCGCTGCCCTCGAACTTCACCT  AAACAGGTGAAGTTCGAGGGCAG |
| AN66_pU6.opt-gRNA.OT.6 | opt-gOT.6 | #449  #450 | ACCGTCATCCTCGAACTTCACCT  AAACAGGTGAAGTTCGAGGATGA |
| AN67_pU6.opt-gRNA.OT.7 | opt-gOT.7 | #451  #452 | ACCGTCGCTTTCGAACTTCACCT  AAACAGGTGAAGTTCGAAAGCGA |
| AN68_pU6.opt-eGFP.OT.8 | opt-gOT.8 | #453  #454 | ACCGTCGCCCCTGAACTTCACCT  AAACAGGTGAAGTTCAGGGGCGA |
| AN69_pU6.opt-gRNA.OT.9 | opt-gOT.9 | #455  #456 | ACCGCTACCCTCGAACTTCACCT  AAACAGGTGAAGTTCGAGGGTAG |
| AN70_pU6.opt-gRNA.OT.10 | opt-gOT.10 | #457  #458 | ACCGTCGTTTTCGAACTTCACCT  AAACAGGTGAAGTTCGAAAACGA |

**Supplementary Table 11. Transfection scheme for investigating the off-target activities of Cas9, eCas9.2NLS and** eCas9.4NLS when coupled to mismatch-containing opt-gRNA

| H27 cells | 3 ×10^4^ cells per well of 24-well plates | | | | | |
| --- | --- | --- | --- | --- | --- | --- |
|  | PEI： 3.29 µl per well | | | | | |
| Encoded products | Cas9 | eCas9.2NLS | eCas9.4NLS | opt-gRNAs  (Supplementary Table 10) | DsRed | Total  (ng) |
| Construct length  (bp) | 9215 | 9360 | 9403 | 3057 | 4712 |  |
| DNA per well  (ng) | 271,5 |  |  | 89,8 | 138,8 | 500 |
|  |  | 271,5 |  | 89,8 | 138,8 | 500 |
|  |  |  | 271,5 | 89,8 | 138,8 | 500 |

**Supplementary Table 12. Transfection scheme for investigating activity of Cas9, eCas9.2NLS and** eCas9.4NLS in HEK.EGFP^TetO.KRAB^ cells exposed and not exposed to doxycycline

|  | 1.5×10^5^  HEK.EGFP^TetO.KRAB^ cells per well of 24-well plates  PEI: 3.29 µl per well | | | | | | | |
| --- | --- | --- | --- | --- | --- | --- | --- | --- |
| Construct | Cas9 | eCas9.2NLS | eCas9.4NLS | gRNA^empty^ | gRNA^eGFP.8^ | gRNA^eGFP.9^ | DsRed | Total (ng) |
| Length (bp) | 9215 | 9360 | 9403 | 3915 | 3974 | 3046 | 4712 |  |
| DNA per well (ng) | 392.2 |  |  | 164.0 |  |  | 193.8 | 750 |
|  | 390.4 |  |  |  | 165.8 |  | 193.8 | 750 |
|  | 419.6 |  |  |  |  | 136.6 | 193.8 | 750 |
|  |  | 392.2 |  | 164.0 |  |  | 193.8 | 750 |
|  |  | 390.4 |  |  | 165.8 |  | 193.8 | 750 |
|  |  | 419.6 |  |  |  | 136.6 | 193.8 | 750 |
|  |  |  | 392.2 | 164.0 |  |  | 193.8 | 750 |
|  |  |  | 390.4 |  | 165.8 |  | 193.8 | 750 |
|  |  |  | 419.6 |  |  | 136.6 | 193.8 | 750 |
